# An effective strategy for assembling the sex-limited chromosome

**DOI:** 10.1093/gigascience/giae015

**Published:** 2024-04-16

**Authors:** Xiao-Bo Wang, Hong-Wei Lu, Qing-You Liu, A-Lun Li, Hong-Ling Zhou, Yong Zhang, Tian-Qi Zhu, Jue Ruan

**Affiliations:** Shenzhen Branch, Guangdong Laboratory for Lingnan Modern Agriculture, Genome Analysis Laboratory of the Ministry of Agriculture and Rural Affairs, Agricultural Genomics Institute at Shenzhen, Chinese Academy of Agricultural Sciences, Shenzhen, Guangdong 518120, China; The Shennong Laboratory/Institute of Crop Molecular Breeding, Henan Academy of Agricultural Sciences, Zhengzhou 450002, China; Shenzhen Branch, Guangdong Laboratory for Lingnan Modern Agriculture, Genome Analysis Laboratory of the Ministry of Agriculture and Rural Affairs, Agricultural Genomics Institute at Shenzhen, Chinese Academy of Agricultural Sciences, Shenzhen, Guangdong 518120, China; Guangdong Provincial Key Laboratory of Animal Molecular Design and Precise Breeding, School of Life Science and Engineering, Foshan University, Foshan 528225, China; Shenzhen Branch, Guangdong Laboratory for Lingnan Modern Agriculture, Genome Analysis Laboratory of the Ministry of Agriculture and Rural Affairs, Agricultural Genomics Institute at Shenzhen, Chinese Academy of Agricultural Sciences, Shenzhen, Guangdong 518120, China; Shenzhen Branch, Guangdong Laboratory for Lingnan Modern Agriculture, Genome Analysis Laboratory of the Ministry of Agriculture and Rural Affairs, Agricultural Genomics Institute at Shenzhen, Chinese Academy of Agricultural Sciences, Shenzhen, Guangdong 518120, China; Key Laboratory of Zoological Systematics and Evolution & State Key Laboratory of Integrated Management of Pest Insects and Rodents, Institute of Zoology, Chinese Academy of Sciences, Beijing 100101, China; National Center for Mathematics and Interdisciplinary Sciences, Academy of Mathematics and Systems Science, Chinese Academy of Sciences, Beijing 100190, China; Key Laboratory of Random Complex Structures and Data Science, Academy of Mathematics and Systems Science, Chinese Academy of Sciences, Beijing 100190, China; Shenzhen Branch, Guangdong Laboratory for Lingnan Modern Agriculture, Genome Analysis Laboratory of the Ministry of Agriculture and Rural Affairs, Agricultural Genomics Institute at Shenzhen, Chinese Academy of Agricultural Sciences, Shenzhen, Guangdong 518120, China

**Keywords:** sex-limited chromosome, assembly method, long-read sorting

## Abstract

**Background:**

Most currently available reference genomes lack the sequence map of sex-limited (such as Y and W) chromosomes, which results in incomplete assemblies that hinder further research on sex chromosomes. Recent advancements in long-read sequencing and population sequencing have provided the opportunity to assemble sex-limited chromosomes without the traditional complicated experimental efforts.

**Findings:**

We introduce the first computational method, Sorting long Reads of Y or other sex-limited chromosome (SRY), which achieves improved assembly results compared to flow sorting. Specifically, SRY outperforms in the heterochromatic region and demonstrates comparable performance in other regions. Furthermore, SRY enhances the capabilities of the hybrid assembly software, resulting in improved continuity and accuracy.

**Conclusions:**

Our method enables true complete genome assembly and facilitates downstream research of sex-limited chromosomes.

## Introduction

Traditionally, genomes of homogametic individuals (XX females or ZZ males) have been preferred for genome sequencing projects, because the haploid nature of both sex chromosomes (X and Y or Z and W) in heterogametic species reduces sequencing depth, which can lead to decreased assembly contiguity and length [1]. Although XY or ZW chromosomes have significantly diverged from their ancestral autosomes [2, 3], they still exhibit homology, which can pose challenges for genome assembly. Homologous regions, such as the pseudoautosomal regions (PARs), can result in fragmented contigs similar to large repeats. Plenty of repetitive sequences in a sex-limited (Y or W) chromosome further increase the assembly difficulties.

**Figure 1:**
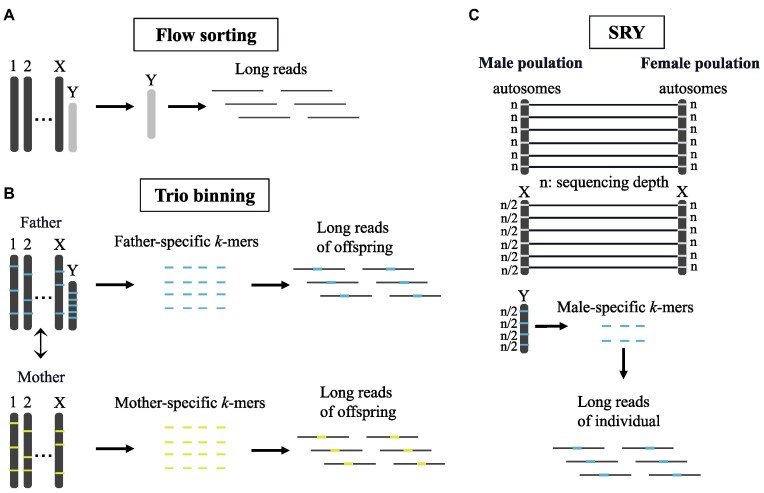
Overview of 3 methods for sorting long reads. (A) Flow sorting is an experimental method for separating the Y chromosome. (B) Trio binning compares *k*-mers from short reads of parent genomes and identifies father-specific and mother-specific *k*-mers, respectively. These specific *k*-mers are used to bite long reads for each parent. (C) SRY rules out *k*-mers presenting in both male and female populations and retains *k*-mers that only occurred in the male population with a half-sequencing depth. SRY utilizes these male-specific *k*-mers to separate long reads of the Y chromosome.

Currently, there are 2 main experimental approaches aimed at solving the problem. The first approach, known as the BAC-based method, has been applied in deciphering the Y chromosomes of several mammals, including human [3], chimpanzee [4], rhesus macaque [5], and mouse [6]. However, it is time-consuming, labor-intensive, and expensive. The second approach is chromosome flow sorting, which relies on chromosome size and GC content (Fig. 1A) and offers high automation and throughput [1]. However, it requires cells to be in metaphase, where chromosomes are in a condensed state that can be easily physically separated [7]. Additionally, it may mistakenly sort other chromosomes or debris with similar sizes or GC contents to the sex-specific chromosome, which can introduce bias during the amplification stage [1, 8].

Akagi et al. [9] used the F_1_ population data of the persimmon to identify male-specific markers and utilized these markers to partition and assemble short reads. However, they did not provide software for the algorithm or consider the effect of population heterogeneity on the identification of male-specific markers. YGS [10] compares the male assembly results with length *k* subsequences (*k*-mers) of female short reads to obtain Y contigs. The Sex-detector [11] uses pedigree data to identify sex-specific genes in RNA sequencing assemblies. However, all 3 methods lack the ability to sort long reads to reduce the assembling difficulty.

Thanks to the longer read lengths and higher sequencing accuracy, long reads have a higher potential to be identified to their original chromosome by pure computing methods. Recently, trio binning was developed to sort long reads *in silico* [12] using specific markers (Fig. 1B). It compares *k*-mers of short reads from parental genomes and identifies *k*-mers that are unique to each parent. These *k*-mers are then used to separate long reads of the offspring and conduct haplotype assemblies, separately. Theoretically, Y- (or W-) specific markers [13] can be selected and used for sorting long reads from the sex-limited chromosome. Compared to whole genome shotgun (WGS) assembly, trio binning assembly covers more genomic regions of the Y chromosome with better contiguity (Supplementary Table S4). It indicates that the computational method is promising, although trio binning cannot efficiently address the problem of assembling sex-limited chromosome based on its scheme to select specific markers. The Telomere-to-Telomere (T2T) consortium has used various third-generation sequencing technologies to complete the assembly of the Y chromosome [13], but the process requires substantial manual adjustments. It is worth noting that recent marker-based graph phasing algorithms in long reads, such as hifiasm [14] trio and Verkko [15] trio mode, have emerged as alternative approaches to enhance the accuracy and efficiency of phasing. Specially, Verkko is a successor of the manual efforts taken in T2T-Y. Additionally, there are alternative approaches for phasing genomes in plants. For instance, Serra Mari et al. [16] proposed a polyploid potato phasing method that utilizes many siblings of the child for genotyping and achieved significant results.

To further improve the quality of sex-specific chromosome assembly and reduce the need for manual curation, we try to find new solutions from population datasets. In whole genome sequencing, the sequencing depth between sex chromosomes and autosomes is different. For example, in a XY male, the sequencing depth of the X or Y chromosome is half that of autosomes. Therefore, X/Y-specific markers can be separated by different sequencing depth. Moreover, the X-specific markers also exist in females, so they can be removed from X/Y-specific markers to obtain Y-specific markers. By incorporating population data into our analysis, we anticipate a reduction in the impact of sequencing coverage and allelic genotype variations when identifying Y chromosome–specific markers in a comparative analysis involving only 2 individuals of different genders.

## Results

### Overview of SRY

To reach the goal of sorting long reads of sex-limited chromosome, we developed an *in silico* sorting method called Sorting long Reads of Y or other sex-limited chromosome (SRY) (Fig. 1C). The process of SRY involves identifying sex-specific markers by comparing male and female populations and subsequently sorting long sequences according to the specific markers. SRY first selects *k*-mers with half of the sequencing depth in male populations. Subsequently, SRY filters out X-linked *k-*mers and *k*-mers originating from heterozygous sites found in female populations, thereby enabling the identification of male-specific *k-*mers (MSK). Due to the impact of population structure and sequencing errors, the operation of SRY is in fact a sampling process, which unavoidably involves *k*-mers from X chromosome and autosomes. Therefore, SRY calculates the MSK density of long reads and excludes those with lower marker density. These separated long reads are subsequently delivered to the assemblers to perform genome assembly. Moreover, MSK can be used to select Y chromosome contigs from a whole genome assembly from a male individual [17–19].

### Evaluating SRY with theoretical models and simulated data

**Figure 2:**
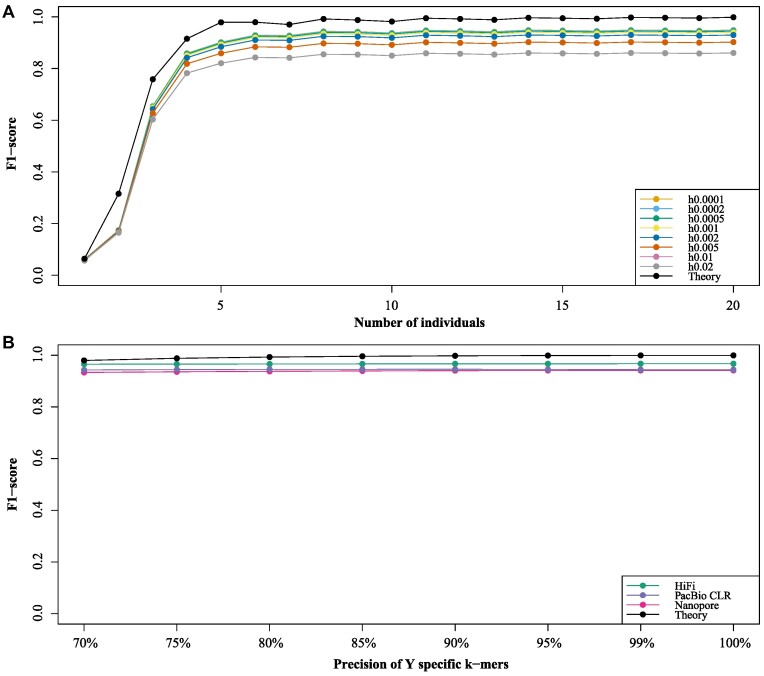
Theoretical model and performance of SRY on simulated data. (A) The F1-score of SRY on identifying male-specific *k*-mers with different individual number (per sex) and population heterogeneity, respectively. For simplicity, we did not consider the similarity distribution between the Y chromosome and other chromosomes in the calculation of the theoretical value. (B) The F1-score of SRY on sorting HiFi, PacBio CLR, and Nanopore long reads. The combination of length distribution and specific *k*-mer distribution can cause true-positive rate (TPR) differences between theoretical and simulated data as well as TPR differences within simulated data.

There are 2 primary factors contributing to false positives in the identification of specific *k*-mers by SRY: coverage and population heterogeneity. Accordingly, we constructed theoretical models of the false positive and true positive to assess MSK identified by SRY (see the Methods section for details). In addition, we used the mason_simulator [20] software to simulate the data under different heterogeneity and different number of individuals (5× for each individual) for the evaluation of SRY. Consistent with the theoretical results, the results based on simulated data show that an increase in population heterogeneity decreases the F1-score of SRY, while an increase in the number of individuals augments the F1-score of SRY (Fig. 2A). Notably, when the number of individuals in both male and female populations is fewer than 7, the increase in the number of individuals has a significant effect on improving the F1-score of SRY (Fig. 2A). However, once the number exceeds 7, the increase in the number of individuals has little effect (Fig. 2A).

Furthermore, we provide a theoretical model for the process of sorting long reads of Y chromosome (see Methods section for details). The central issue addressed by this model is determining the probability that at least M markers are retained in the corresponding error-prone long reads within a genomic region consisting of N specific markers. In addition to theoretical values, we used badread [21] software to simulate the human T2T genome with 50× each of HiFi, Nanopore, and PacBio CLR reads and set a range of MSK precision level to assess the performance of SRY on sorting long reads. Remarkably, even when the precision of MSK decreases to 70%, we found that the F1-score of SRY remains above 90% (Fig. 2B). This can be attributed to the fact that the genome size of autosomes and X chromosome is approximately 3 Gb, resulting in a low density of nonspecific *k*-mers (non-MSK) derived from these chromosomes (1 *k*-mer/kb). The filter criterion of SRY is ∼7 *k*-mers per kilobase, making it easy to exclude these non–Y chromosome sequences.

### Comparison with the experimental method on real data

**Figure 3:**
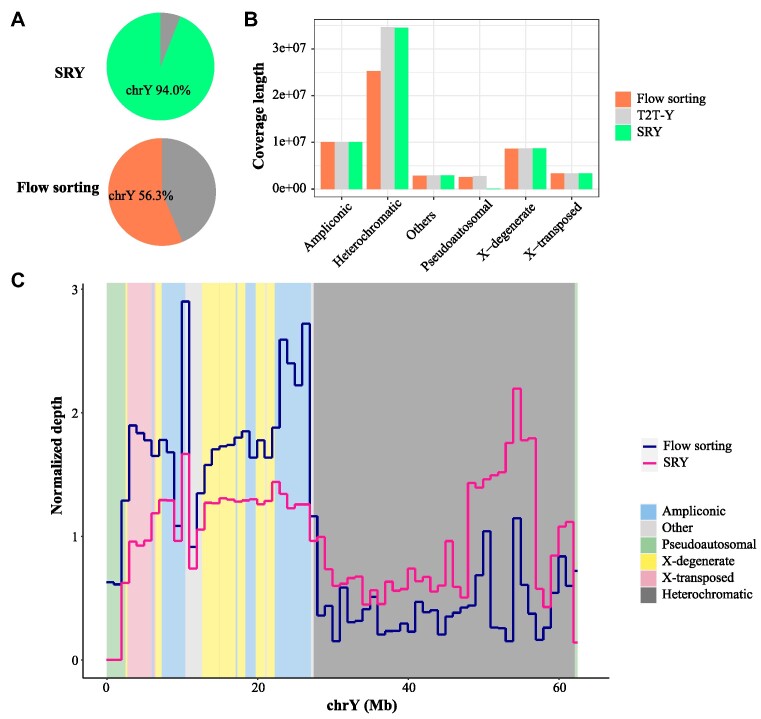
Comparison of sorting results between SRY and flow sorting. (A) Alignment distribution of sorted reads for SRY and flow sorting. The alignment on autosomes and the X chromosome is colored in gray. (B) The coverage of sorting reads by the 2 methods on discrete regions of the Y chromosome. SRY aims to separate male-specific long reads, so the coverage is lower on the pseudoautosomal region where recombination events occur frequently between X and Y chromosomes. (C) The normalized depth of long reads separated by the 2 methods. The colored rectangles represent discrete regions on the T2T-Y chromosome.

We collected datasets including short and long reads of a Chinese individual HX1 [22, 23] and resequencing short reads of a Han Chinese population [24] to identify MSK (Supplementary Tables S1 and S2). SRY obtained about 10 million MSK as well as sorted 3.7 G (∼46×) PacBio CLR and (∼13×) ONT long reads of the Y chromosome (Supplementary Table S3). We further collected Nanopore long reads (∼2.3 G, number of reads is 305,284) of an African human Y (HG02982) separated by flow sorting [8] and used minimap2 [25] to align the sorted long reads from the 2 methods to the human T2T genome, separately. The results show that 94.0% of the sorted reads from SRY are mapped on the T2T-Y chromosome, which is significantly higher than that of flow sorting (Fig. 3A). The human Y chromosome consists of several distinct regions (ampliconic, X-degenerate, X-transposed, pseudoautosomal, heterochromatic, others) [3]. We compared the performance of the 2 methods in these regions and found that SRY demonstrates comparable performance to flow sorting in the ampliconic, X-degenerate, X-transposed, and others (Fig. 3B). However, SRY outperforms in terms of coverage and depth specifically in the heterochromatic region (Fig. 3B, C and Supplementary Table S4). Notably, the event of X–Y recombination is frequent on the pseudoautosomal region (PAR, PAR1: 1–2.8 Mb, PAR2: 56.9–57.2 Mb). SRY aims to obtain Y-specific markers, and hence the low coverage and shorter assembled result on PAR of SRY is expected (Fig. 3B and Supplementary Table S4).

We further compared the resulting assemblies between the experimental and computational methods. We collected a trio dataset consisting of the parental genomes HG01107 and HG01108, as well as the offspring genome HG01109, in order to perform trio binning and compare the resulting assemblies of HG01109. SRY can sort reads first and then assemble or directly sort the assembled contigs based on MSK. The fast assembler wtdbg2 [26] was used to assemble those sorted reads and flow-sorting reads, as well as perform genome assemblies for trio binning and WGS (Supplementary Table S3). The total contig alignment length on the T2T-Y chromosome from SRY is ∼5.7 Mb and ∼9.6 Mb longer than those from the sorted contigs of trio binning and WGS, respectively. Moreover, the alignment lengths and the contiguity (NA50) on each discrete region of the Y chromosome from SRY are all longer than the other 2 methods (Supplementary Table S5), indicating that it is better to sort the reads first and then assemble them. Additionally, the SRY assembly exhibits lower contamination from other chromosomes compared to flow sorting (Supplementary Table S4). Similarly to the result obtained from read sorting, the assembly result from SRY performed better in heterochromatic regions compared to flow sorting. However, in the PAR, the assembly result from SRY was inferior to those from flow sorting.

### Toward complete genome assembly of the Y chromosome

**Figure 4:**
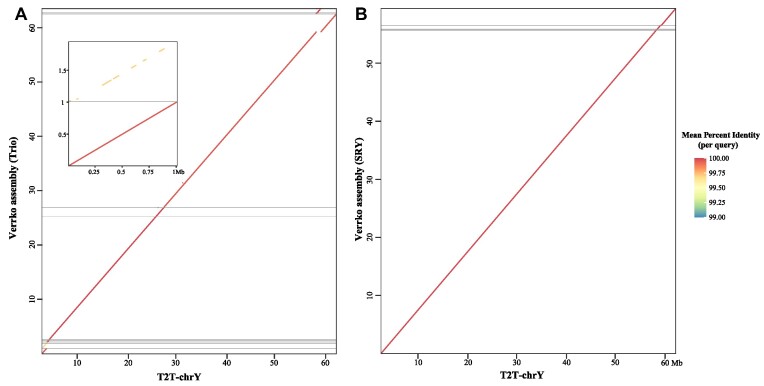
Verkko assembly using Trio or SRY. The x-axis represents the complete Y chromosome assembled by the T2T consortium, and y-axes represent assemblies by Verkko with trio (A) and SRY (B), respectively. Inset in (A) represents an enlarged view of the first 1-Mb region of the T2T-chrY. The diagonal colored with identity shows the alignment result. (Note: T2T-Y palindrome P5 [18483410–19235627] is expected to be inverted, and this error was not resolved by the assembly using Verkko SRY mode.)

Rautiainen et al. [15] developed an assembly software called Verkko, designed for HiFi and ultra-long Nanopore data, in order to achieve better automation of T2T-level chromosome assembly. Verkko demonstrated good result in the assembly of HG002. We sorted the Y chromosome data from HG002 and used Verkko for assembly. The results showed that, compared to Verkko with trio [15], the assembly of the sorted data (Verkko with SRY) reduced the number of contigs from 23 to 9 and corrected 1 assembly error (Fig. 4). Additionally, due to the high similarity of the X and Y chromosome PAR regions, Verkko trio’s assembly result did not phase this region well, resulting in 2 approximately 1-Mb contigs aligning to the same region of the Y chromosome (Fig. 4A). Verkko SRY assembled this region not only completely but also with higher accuracy (Fig. 4B). This indicates that SRY can further improve the performance of the assembly software with new sequencing technology.

We further selected the genome of the yellow catfish [27], which contains young Y chromosomes, for simulated evaluation. The similarity between its Y chromosome and X chromosome exceeds 99%. To facilitate evaluation, we removed the gaps in the genome. We simulated 100× HiFi and ultra-long Nanopore data (50× for the Y chromosome), respectively. As a result, we sorted ∼45× HiFi data and ∼37× ultra-long Nanopore data of the Y chromosome. The assembly size of the results using Verkko software was 42.7Mb (98.8% of the Y chromosome), consisting of only 2 contigs. According to the quast results, both contigs aligned almost perfectly to the reference Y chromosome. This indicates that our software can achieve relatively good results in assembling young Y chromosomes as well.

## Discussion

The deciphering of sex chromosomes is crucial for studying reproductive biology, sex determination, and other key molecular processes that contribute to the evolutionary trajectory of species. To enable more complete assembly of sex chromosome sequences, we have developed the SRY method, a software tool that efficiently sorts third-generation long reads of sex-limited chromosomes based on MSK markers.

The performance of SRY can be influenced by the number of male and female individuals, whereas flow sorting only requires 1 male individual of interest and is not affected. However, SRY outperformed flow sorting in terms of read sorting accuracy and demonstrated comparable or better performance in most regions, except for the PAR where SRY had lower coverage and assembly results. The effectiveness of SRY is also influenced by the lengths of the reads and the quality of the base pairs. Notably, the incorporation of HiFi and ultra-long Nanopore data significantly improved the assembly quality of SRY in the PAR region. The capability of SRY to effectively sort long reads of sex-limited chromosomes (regardless of their age) highlights its potential as a valuable alternative to experimental methods for studying sex-specific genomic regions.

With the further reduction in sequencing costs, there will be a greater availability of population-level second- and third-generation sequencing data. After identifying MSK using second-generation data, SRY can be utilized for sorting and assembling third-generation long reads from all individuals within the population. The application and comparison of sex chromosomes within the population will contribute to our understanding of complex biological processes and genetic variations.

## Methods

### SRY process

First, SRY used the kmer_count program to acquire *k*-mer (*k* = 21) [12] sets from short reads of targeted male species and populations. Next, the program filterx [28] was used to identify specific *k*-mers associated with the male population. We labeled the *k*-mer files of all male individuals as “group1” and all female individuals as “group2.” By comparing these groups, we identified *k*-mers as specific *k*-mers that are present in at least 2/3 of the individuals from group1. Then, SRY selects long reads of targeted species that have MSK. Finally, SRY filters those long reads with lower MSK densities than the average value of whole Y chromosome.

### False positive for MSK

False positive is introduced if a subsequence of length *k* (k-mer) originated from autosomes or X chromosome is incorrectly identified as a MSK, with 2 possible sources: genomic coverage and population heterogeneity.

In the model, *n* males and *n* females are sequenced with the sequencing depth *d*. The length of a read is *l* and the sequencing error per site is *r*. $C_n^i$ represents the number of ways to choose *i* elements from a set of *n* elements, also known as the binomial coefficient. In an individual, a *k*-mer is considered present only if it appears more than once, and this event occurs with probability *p*_o_. A *k*-mer is identified as an MSK if it is present in at least *m* males but not in any of the females. Particularly, we use *m* = 2/3*n* as the critical value by simulation study.

Let *X* be the frequency of appearance of a *k*-mer that is present in an individual, and then it follows a Possion distribution with rate *λ* = *d*(1 *– r*)*^k^*(*l − k* + 1)/*l*. It is easier to calculate *q*_o_ = 1 *− p*_o_, which is the probability that a *k*-mer is absent, and thus


\begin{eqnarray*}
{q}_o = P\left( {X = 0} \right) + P\left( {X\ = 1} \right) = {e}^{ - \lambda } + \lambda {e}^{ - \lambda } = (1 + \lambda ){e}^{ - \lambda }
\end{eqnarray*}

We then can calculate the false positive rate caused by genomic coverage (*f*_1_) by


(1) \begin{eqnarray*}
{f}_1 = q_{\mathrm{o}}^n\sum\limits_{i = \left\lceil {{\textstyle{2 \over 3}}n} \right\rceil }^n {C_n^ip_{\mathrm{o}}^iq_{\mathrm{o}}^{n - i}}
\end{eqnarray*}

If we set *r* = 0.01, *l* = 150, *d* = 5, *k* = 21, and *n* = 5, then *f*_1_ is roughly 4.4 × 10^−5^. If the sample size *n* increases to 10, then *f*_1_ decreases to 1.9 × 10^−9^, indicating that the false positive introduced by genomic coverage can be ignored if the sample size *n* is not too small.

For simplicity, we only consider heterogeneity in autosomes and ignore heterogeneity in the X chromosome. Assume 1 heterozygous site leads to 2 kinds of *k*-mers (*k*-mer-1 and *k*-mer-2), and the heterozygous proportions for 2 *k*-mers are *p_h_*_1_ and *p_h_*_2_ (with *p_h_*_1_ + *p_h_*_2_ = 1). If *k*-mer-*j* (*j* = 1,2) from autosomes is observed in many males but is not observed in any females, then *k*-mer-*j* is mistakenly identified as an MSK. As the discussion for the case of genomic coverage, the frequency of appearance of *k*-mer-*j, X_j_*, follows Poisson distributions with parameters *λ_j_* = *p_hj_ λ = p_hj_ d* (1 *– r*)*^k^*(*l − k* + 1)/*l*. As before, if a *k*-mer appears less than twice in an individual, we consider the *k*-mer is absent, and the probability of this event *q*_o_*_j_* can be calculated as follows:


\begin{eqnarray*}
{q}_{oj} = P\left( {{X}_j = 0} \right) + P\left( {{X}_j = 1} \right) = {e}^{ - \lambda j} + {\lambda }_j{e}^{ - \lambda j} = \left( {1 + {\lambda }_j} \right){e}^{ - \lambda j}. \end{eqnarray*}

Given the heteropoietic rate *h* = 0.001, the probability that a *k*-mer with length 21 contains more than 1 heterozygous site is only 2.1 × 10^−4^, which can be neglected. We also ignore the probability that *k*-mer-1 is identified as *k*-mer-2 mistakenly with edit distance 1 due to sequencing error, as such events occur with probability 0.99^20^*r/3 = 0.3%. Then the false-positive rate due to population heterogeneity *f*_2_ can be calculated as follows:


(2) \begin{eqnarray*}
{f}_2 = q_{{\mathrm{o1}}}^n\sum\limits_{\left\lceil {i = {\textstyle{2 \over 3}}n} \right\rceil }^n {C_n^ip_{{\mathrm{o1}}}^iq_{{\mathrm{o1}}}^{n - i}} + q_{{\mathrm{o2}}}^n\sum\limits_{\left\lceil {i = {\textstyle{2 \over 3}}n} \right\rceil }^n {C_n^ip_{{\mathrm{o2}}}^iq_{{\mathrm{o2}}}^{n - i}} . \end{eqnarray*}

The total false-positive rate *f* is a weighted average of the false-positive rate from the 2 sources, that is,


(3) \begin{eqnarray*}
f = \left( {1 - kh} \right){f}_1 + kh{f}_2
\end{eqnarray*}

Note that *f*_2_ is actually a function of *p_hj_*, which is an unknown parameter in the model. We further used the biallelic SNV datasets from the 1000 Genomes Project [29] to estimate the empirical distribution of *p_h_*_1_. We use a discrete distribution ranging from 0 to 0.5 to characterize the distribution of *p_h_*_1_, which takes values of 0.05, 0.15, 0.25, 0.35, and 0.45 with probabilities of 92.77%, 2.59%, 1.80%, 1.48%, and 1.36%, respectively. Combining the uncertainty of the heterozygotic rate, the false-positive rate is


(4) \begin{eqnarray*}
f = (1 - kh){f}_1 + kh\sum\limits_x {{f}_2(x)} P({p}_{h1} = x). \end{eqnarray*}

### True-positive rate (TPR) of identifying MSK

As discussed before, the probability of a *k*-mer present in more than two-thirds of male individuals is $\sum\limits_{i = {\textstyle{2 \over 3}}n}^n {C_n^ip_{{\mathrm{o}}j}^iq_{{\mathrm{o}}j}^{n - i}} $. As the probability that a *k*-mer from autosomes is identified as a MSK due to sequencing error is too small, the probability that an MSK is present in none of the females is roughly 1. Then, the true-positive rate (TPR) of identifying MSK is the product of the probability of the 2 events, that is,


\begin{eqnarray*}
{\rm TPR} = \sum\limits_{i = {\textstyle{2 \over 3}}n}^n {C_n^ip_{{\mathrm{o}}j}^iq_{{\mathrm{o}}j}^{n - i}}
\end{eqnarray*}

### The probability of sorting long reads

For third-generation long reads, the sequencing errors are higher (PacBio CLR or Nanopore) and their lengths vary a lot. Assume a long read contains *N* specific markers, with *N* to be the function of the read length and the distribution of MSK, and the probability that a *k*-mer is correctly sequenced is *p* = (1 − *r*_3_)*^k^* (*r*_3_ is the sequencing error of long reads). Then, the probability that at least *M* MSKs are identified is


\begin{eqnarray*}
\sum\limits_{i = M}^N {C_N^i{p}^i{{(1 - p)}}^{N - i}}
\end{eqnarray*}

In SRY software, the average number of MSKs (about 7/kb) across the Y chromosome is taken as the value of *M* to sort Y-chromosome long reads.

### Assessment

In order to evaluate the MSK identification process of SRY, we first used kmer_count to obtain the *k*-mer of all chromosomes of the human T2T genome and used filterx [28] to identify the specific *k-*mer of the T2T-Y chromosome, which served as the standard for subsequent evaluations. Then, we used the mason_simulator (v2.0.9) program in the mason [20] package with the parameter (–illumina-prob-mismatch 0.009 –illumina-prob-insert 0.0005 –illumina-prob-deletion 0.0005 –illumina-read-length 150) to generate short-read data for male and female populations using the human T2T genome with or without the T2T-Y chromosome as a reference, respectively. We used different seed values for all individuals to avoid the result that the simulated data for all individuals were the same. Finally, SRY used these population data to identify MSKs, which were evaluated by comparison with T2T-Y chromosome-specific *k-*mers.

For the theoretical value of the precision of SRY on identifying MSK, we used the following formula:


\begin{eqnarray*}
{\mathrm{YSK}}*{\mathrm{TPR}}/\left( {{\mathrm{YSK}}*{\mathrm{TPR}} + {\mathrm{AXK}}*{\mathrm{FPR}}} \right) \end{eqnarray*}

where YSK represents the specific *k*-mer number of the T2T-Y chromosome, AXK represents the *k*-mer number of the human T2T autosomes and X chromosome, and TPR represents the true-positive rate and FPR represents the false-positive rate of identifying MSK, respectively.

We further simulated 50× Nanopore, PacBio CLR, and HiFi reads (25× for the Y chromosome) based on the human T2T genomes using the badread [21] package (v0.1.3) with the following commands, respectively:

*badread simulate –reference human_autoX.fa –quantity 50X –error_model nanopore –start_adapter 0,0 –end_adapter 0,0 –junk_reads 0 –random_reads 0 –chimeras 0* (simulated Nanopore reads of autosomes and the X chromosome)

*badread simulate –reference human_Y.fa –quantity 25X –error_model nanopore –start_adapter 0,0 –end_adapter 0,0 –junk_reads 0 –random_reads 0 –chimeras 0* (simulated Nanopore reads of the Y chromosome)

*badread simulate –reference human_autoX.fa –quantity 50X –error_model pacbio –identity 85,95,3 –length 7500,7500 –start_adapter 0,0 –end_adapter 0,0 –junk_reads 0 –random_reads 0 –chimeras 0* (simulated PacBio CLR reads of autosomes and the X chromosome)

*badread simulate –reference human_Y.fa –quantity 25X –error_model pacbio –identity 85,95,3 –length 7500,7500 –start_adapter 0,0 –end_adapter 0,0 –junk_reads 0 –random_reads 0 –chimeras 0* (simulated PacBio CLR reads of the Y chromosome)

*badread simulate –reference human_autoX.fa –quantity 50x –error_model pacbio –qscore_model pacbio –identity 99,100,3 –length 12000,12000 –start_adapter 0,0 –end_adapter 0,0 –junk_reads 0 –random_reads 0 –chimeras 0* (simulated PacBio HiFi reads of autosomes and the X chromosome)

*badread simulate –reference human_Y.fa –quantity 25x –error_model pacbio –qscore_model pacbio –identity 99,100,3 –length 12000,12000 –start_adapter 0,0 –end_adapter 0,0 –junk_reads 0 –random_reads 0 –chimeras 0* (simulated PacBio HiFi reads of the Y chromosome)

SRY was assessed for its ability to sort these simulated long reads by considering different TPR values of MSK.

Even the precision of MSK decreased to 70% (including ∼7,000,000 MSKs and ∼3,000,000 non-MSKs), and the density of these non-MSKs on autosomes and X chromosomes was only 1 per kb, which was significantly lower than the threshold set by SRY (7/kb). Therefore, we took the theoretical precision of SRY on sorting long reads as 1. For simplicity, the process of calculating the TPR of SRY on sorting long reads ignored the length distribution of the reads and used the window with a 10-kb length to calculate the specific *k-*mer distribution of the T2T-Y chromosome.

### Y chromosome assembly, identification, and evaluation

We collected ∼60× ultra-long ONT data and ∼35× HiFi data [15] and used SRY for the long-read sorting of the Y chromosome. Due to the abundance of repetitive sequences in the heterochromatic regions of the Y chromosome, the number of available Y-specific markers is limited. Therefore, we used 2 lengths of *k*-mers (*k* = 21 and *k* = 51) for the sorting of HiFi reads. Verkko (v1.0) [15] was used to assemble the selected data with parameters (-d Asm –hifi hifi.sorted.fq.gz –nano ul-ont.sorted.fq.gz –threads 128). We compared the assembly results of Verkko in trio mode (collected from [15]) and SRY mode to T2T-CHM13 using Quast (v5.0.2) [30]. The file with the suffix name “coords.filtered” was used by DotPlotly [31] (parameters: -slt -m 100 -q 100) to generate the alignment plot.

We collected the dataset of a trio family including short reads from the father (HG01107, ∼113×) and mother (HG01108, ∼79×) and Nanopore reads from the child (HG01109, ∼72×) [32]. SRY separated 1.3 G (∼25×) long reads of HG01109 using MSK markers identified from HX1. Then, we used wtdbg2.5 [26] with parameters “-L 0 -p 0 -k 21 -s 0.25 -S 2 –rescue-low-cov-edges” to assemble those long reads. The remaining long reads were assembled by wtdbg2.5 with parameters “-x ont -g 3g” and polished with the program wtpoa-cns in wtdbg2.5. All of the assembled contigs were further polished with wtpoa-cns using short reads. Sorting of long reads and genome assemblies for the other 9 individuals was performed the same way. Trio binning phased HG01109 long reads with the command “canu -stopAfter=haplotype genomeSize=3 g -haplotypeMale HG01107.fastq.gz -haplotypeFemale HG01108.fastq.gz -nanopore-raw HG01109.fasta.tar.bz2.” Wtdbg2 with the parameters (-g 3.1 G -x ont) was applied to assemble phasing reads from trio binning and perform whole genome assembly for WGS. SRY was then used to partition candidate contigs of the Y chromosome for trio binning and WGS. We utilized quast (v5.0.2) [30] with default parameters to evaluate the assembled genome quality.

## Availability of Supporting Source Code and Requirements

Project name: SRY: Sorting long Reads of Y or other sex-limited chromosome

Project homepage: https://github.com/caaswxb/SRY

Operating system(s): Linux or Unix

Programming language: shell and perl

Other requirements: kmc, samtools, seqtk, and parallel

License: MIT License

RRID: SCR_025036

Biotools ID: SRY

## Supplementary Material

giae015_GIGA-D-23-00223_Original_Submission

giae015_GIGA-D-23-00223_Revision_1

giae015_GIGA-D-23-00223_Revision_2

giae015_Response_to_Reviewer_Comments_Original_Submission

giae015_Response_to_Reviewer_Comments_Revision_1

giae015_Reviewer_1_Report_Original_Submission Zuyao Liu, Ph.D -- 9/1/2023 Reviewed

giae015_Reviewer_1_Report_Revision_1 Zuyao Liu, Ph.D -- 1/25/2024 Reviewed

giae015_Reviewer_2_Report_Original_Submission Shilpa Garg -- 10/16/2023 Reviewed

giae015_Reviewer_2_Report_Revision_1 Shilpa Garg -- 2/21/2024 Reviewed

giae015_Reviewer_3_Report_Original_Submission Arang Rhie -- 10/22/2023 Reviewed

giae015_Supplemental_File

## Data Availability

We downloaded all Nanopore, PacBio, and Illumina datasets of HX1 via NCBI, project number PRJNA301527 [22, 23]. The SRA numbers of the Han Chinese population are listed in Supplementary Tables S1 and S2. The trio family (HG01107, HG01108, and HG01109), HG005, HG006, HG01243, HG02055, HG03098, and HG03492 reads are available at Amason S3 [33]. Short reads as well as PacBio and/or Nanopore long reads of HG002 and HG003 are available at the National Center for Biotechnology Information (NCBI) [34]. We also downloaded NCBI accession ERR3241824 for HG01107 and NCBI accession ERR3241825 for HG01108 to improve the performance of trio binning. All assembly results have been submitted to Figshare [35]. An archival copy of the code and supporting data is also available via the *GigaScience* database, GigaDB [36].

## Additional Files

**Supplementary Table S1**. Summary of datasets for 46 males of the Han Chinese population.

**Supplementary Table S2**. Summary of datasets for 44 females of the Han Chinese population.

**Supplementary Table S3**. Statistics of PacBio and Nanopore reads identified by SRY from long-read sequencing datasets. P represents PacBio CLR reads, and N represents Nanopore reads. The average error rate is calculated based on the alignment results of the third-generation data on the human T2T genome.

**Supplementary Table S4**. Comparison of the assembled genomes for SRY and flow sorting. Human sample numbers are in parentheses, and the sample numbers and metrics of the other 0 individuals are detailed in Supplementary Table S6.

**Supplementary Table S5**. Contig sorting for the HG01109 assembly results of trio binning and WGS using SRY.

**Supplementary Table S6**. Statistics of 9 human assemblies.

## Abbreviations

BAC: bacterial artificial chromosome; PAR: pseudoautosomal region; SRY: Sorting long Reads of Y or other sex-limited chromosome; MSK: male-specific *k-*mers; NCBI: National Center for Biotechnology Information; TPR: true positive rate; T2T: Telomere-to-Telomere; WGS: whole genome shotgun.
