# Supplementary material for: An effective strategy for assembling the sex-limited chromosome
Source: Gigascience. 2024 Apr 16;13:giae015. doi: 10.1093/gigascience/giae015 (PMC11020242; doi:10.1093/gigascience/giae015)
Supplement: giae015_GIGA-D-23-00223_Original_Submission [file giae015_giga-d-23-00223_original_submission.pdf]

GigaScience

An effective strategy for assembling the sex-limited chromosome

--Manuscript Draft--

**Manuscript Number:**	GIGA-D-23-00223
**Full Title:**	An effective strategy for assembling the sex-limited chromosome
**Article Type:**	Technical Note
**Funding Information:**	National Natural Science Foundation of China (91731304) — Prof Jue Ruan<br>National Key Research and Development Program of China (2019YFA0707003) — Prof Jue Ruan<br>National Natural Science Foundation of China (31860638) — Prof Qing-You Liu
**Abstract:**	Most available reference genomes lack the sequence map of sex-limited (such as Y and W) chromosomes, which make the assemblies incomplete and difficult to conduct further research on sex chromosomes. Recent advances in long reads sequencing and population sequencing raise the opportunity to assemble sex-limited chromosomes without the traditional complicated experimental efforts. We introduced the first computational method, Sorting long Reads of Y or other sex-limited chromosome (SRY), which achieved improved assembly results compared to flow sorting. Specifically, SRY outperformed in the heterochromatic region and demonstrated comparable performance in other regions. Furthermore, we observed that SRY enhanced the capabilities of the hybrid assembly software, as evidenced by improved continuity and accuracy. Our method can realize true complete genome assembly and facilitate downstream research of sex-limited chromosomes.
**Corresponding Author:**	xiaobo wang<br>Chinese Academy of Agricultural Sciences Agricultural Genomes Institute at Shenzhen<br>Shenzhen, CHINA
**Corresponding Author Secondary Information:**	
**Corresponding Author's Institution:**	Chinese Academy of Agricultural Sciences Agricultural Genomes Institute at Shenzhen
**Corresponding Author's Secondary Institution:**	
**First Author:**	xiaobo wang
**First Author Secondary Information:**	
**Order of Authors:**	xiaobo wang<br>Hong-Wei Lu<br>Qing-You Liu<br>A-Lun Li<br>Hong-Ling Zhou<br>Yong Zhang<br>Tian-Qi Zhu<br>Jue Ruan
**Order of Authors Secondary Information:**	
**Additional Information:**	
**Question**	**Response**

Powered by Editorial Manager® and ProduXion Manager® from Aries Systems Corporation

Are you submitting this manuscript to a special series or article collection?	No
**Experimental design and statistics**<br><br>Full details of the experimental design and statistical methods used should be given in the Methods section, as detailed in our Minimum Standards Reporting Checklist. Information essential to interpreting the data presented should be made available in the figure legends.<br><br>Have you included all the information requested in your manuscript?	Yes
**Resources**<br><br>A description of all resources used, including antibodies, cell lines, animals and software tools, with enough information to allow them to be uniquely identified, should be included in the Methods section. Authors are strongly encouraged to cite Research Resource Identifiers (RRIDs) for antibodies, model organisms and tools, where possible.<br><br>Have you included the information requested as detailed in our Minimum Standards Reporting Checklist?	Yes
**Availability of data and materials**<br><br>All datasets and code on which the conclusions of the paper rely must be either included in your submission or deposited in publicly available repositories (where available and ethically appropriate), referencing such data using a unique identifier in the references and in the "Availability of Data and Materials" section of your manuscript.<br><br>Have you have met the above requirement as detailed in our Minimum	Yes

Standards Reporting Checklist?	

Powered by Editorial Manager® and ProduXion Manager® from Aries Systems Corporation

Click here to access/download;Manuscript;Manuscript-20230731.docx

An effective strategy for assembling the sex-limited chromosome

Xiao-Bo Wang[1#], Hong-Wei Lu[1#], Qing-You Liu[2#], A-Lun Li[1] , Hong-Ling Zhou[1], Yong Zhang[5], Tian-Qi Zhu[3,4*], Jue Ruan[1*]

1. Shenzhen Branch, Guangdong Laboratory for Lingnan Modern Agriculture, Genome Analysis Laboratory of the Ministry of Agriculture and Rural Affairs, Agricultural Genomics Institute at Shenzhen, Chinese Academy of Agricultural Sciences, Shenzhen, Guangdong 518120, China
2. Guangdong Provincial Key Laboratory of Animal Molecular Design and Precise Breeding, School of Life Science and Engineering, Foshan University, Foshan 528225, China
3. National Center for Mathematics and Interdisciplinary Sciences, Academy of Mathematics and Systems Science, Chinese Academy of Sciences, Beijing 100190, China
4. Key Laboratory of Random Complex Structures and Data Science, Academy of Mathematics and Systems Science, Chinese Academy of Sciences, Beijing 100190, China
5. Key Laboratory of Zoological Systematics and Evolution & State Key Laboratory of Integrated Management of Pest Insects and Rodents, Institute of Zoology, Chinese Academy of Sciences, Beijing 100101, China

[#]These authors contributed equally to this work.

[*]Corresponding author: E-mail: ruanjue@caas.cn; zhutq@amss.ac.cn

Abstract
Most available reference genomes lack the sequence map of sex-limited (such as Y and W) chromosomes, which make the assemblies incomplete and difficult to conduct further research on sex chromosomes. Recent advances in long reads sequencing and population sequencing raise the opportunity to assemble sex-limited chromosomes without the traditional complicated experimental efforts. We introduced the first computational method, Sorting long Reads of Y or other sex-limited chromosome (SRY), which achieved improved assembly results compared to flow sorting. Specifically, SRY outperformed in the heterochromatic region and demonstrated comparable performance in other regions. Furthermore, we observed that SRY enhanced the capabilities of the hybrid assembly software, as evidenced by improved continuity and accuracy. Our method can realize true complete genome assembly and facilitate downstream research of sex-limited chromosomes.

Introduction
Traditionally, homogametic (XX females or ZZ males) genomes have been preferred for genome sequencing projects, because the haploid nature of both sex chromosomes (X and Y, or Z and W) in heterogametic species provide reduced sequencing depth that can decrease assembly contiguity and length[1]. While XY or ZW chromosomes have diverged significantly from their ancestral autosomes[2, 3], their homology can still pose challenges for genome assembly. Homologous regions, such as the pseudoautosomal regions (PAR), can lead to fragmented contigs similar to large repeats. Plenty of repetitive sequences in sex-limited (Y or W) chromosome further increase the assembly difficulties.

So far, there are principally two experimental approaches aimed to solve the problem. The first one, BAC-based method, was applied on deciphering the Y chromosomes of several mammals including human[3], chimpanzee[4], rhesus macaque[5], and mouse[6]. It employs single-haplotype iterative mapping and sequencing (SHIMS), provides the best solution so far to overcome the difficulty of assembling Y chromosome. Unfortunately, it is time-consuming, labor-intensive and expensive. The other one is chromosome flow-sorting, which is based on chromosome size and GC content (**Fig. 1a**), with high automation and high throughput[1]. However, it requires cells to be in metaphase, where chromosomes are in a condensed state that are easily physically separated[7]. Moreover, it can mistakenly sort other chromosomes or debris having similar sizes or GC contents with sex-specific chromosome, and bring in bias during the amplification stage[1, 8].

Takashi *et al*. used the F_1 population data of the persimmon to identify male-specific markers[9], and utilized these markers to partition and assemble short reads. However, they did not provide software for the algorithm, nor do they consider the effect of population heterogeneity on the identification of male-specific markers. YGS[10] compares male assembly results with length k subsequences (*k*-mers) of female short reads to obtain Y contigs. The Sex-detector[11] uses pedigree data to identify sex-specific genes in RNA-seq assemblies. However, all three methods lack the ability to sort long reads to reduce the assembling difficulty.

Thanks to both the longer read lengths and higher sequencing accuracy, long reads have a higher potential to be identified to its original chromosome by pure computing method. Recently, trio binning was developed to utilize specific markers for long reads sorting *in silico*[12] (**Fig. 1b**). It compares *k*-mers of short reads from parental genomes and identifies *k*-mers that are unique to each parent. Trio binning further uses these *k*-mers to separate long reads of the offspring and conducts *de novo* haplotype assemblies, separately. Theoretically, Y- (or W-) specific markers[13] can be

selected and used for sorting long reads from sex-limited chromosome. Compared to whole genome shotgun (WGS) assembly, trio binning assembly covers more genomic regions of Y chromosome with a better contiguity (**Table 1**). It indicates that computational method is promising, though trio binning cannot efficiently address the problem of assembling sex-limited chromosome based on its scheme to select specific markers. The Telomere-to-Telomere (T2T) consortium has used a variety of third-generation sequencing technologies to complete the assembly of the Y chromosome[14], but the assembly process requires plenty of manual adjustments. In the sexPhase program, SNPs obtained from the short- and long-read alignment are used for the long-read sorting of the X and Y chromosomes. But it relies on pre-existing sex markers and targets only the less differentiated sex chromosomes.

In order to solve the above problems, we try to find new solutions from population datasets. In whole genome sequencing, the sequencing depth between sex chromosomes and autosomes is different. Take XY male as an example, the sequencing depth of X or Y chromosome is half that of autosome. Thus, X/Y-specific markers can be separated by different sequencing depth. As we know, the X-specific markers also exist in female, so they can be removed from X/Y-specific markers to obtain Y-specific markers. But only one male and one female could suffer from sampling error, and their comparison would erroneously identify markers containing heterozygous loci as Y-specific markers. However, population-level data comparisons could reduce sampling error and lessen the impact of the error. Thus, Y-specific markers based on population data can be used to partition long reads and further perform the genome assembly of the Y chromosome.

Results

Overview of SRY

To reach the goal of sorting long reads of sex-limited chromosome, we developed an *in silicon* sorting method called SRY (**Fig. 1c**). The basic principle of SRY is to obtain sex-specific markers by comparing male and female populations, and to sort long sequences according to the specific markers. To be specific, SRY firstly selects *k*-mers with half of the sequencing depth in male populations. Then, SRY filters out X-linked *k*-mers and *k*-mers from heterozygous sites present in female populations to identify male-specific *k*-mers (MSK). Owing to the impact of population structure and sequencing errors, the operation of SRY is in fact a sampling process, which unavoidably involves *k*-mers from X chromosome and autosomes. So SRY calculates MSK density of long reads and excludes those with lower marker density. These separated long reads are further delivered to assemblers to perform genome assembly. Also, MSK can be used to select Y chromosome contigs from a whole genome assembly from a male individual[15-17].

Evaluating SRY with theoretical models and simulated data

There are two main sources of false positives for the identification of specific *k*-mers by SRY, one is coverage and the other is population heterogeneity. Accordingly, we constructed theoretical models of the false positive and true positive to assess MSK identified by SRY (see the Methods section for details). In addition, we used the mason_simulator software to simulate the data under different heterogeneity and different number of individuals (5X for each individual) for the evaluation of SRY. Consistent with the theoretical results, the results based on simulated data show that an increase in population heterogeneity lessens the F1-score of SRY, while an increase in the number of individuals augments the F1-score of SRY (**Fig. 2a**). When the number of individuals in

both male and female populations is less than 7, the increase in the number of individuals has a significant effect on improving the F1-score of SRY (**Fig. 2a**). However, when the number is more than 7, the increase in the number of individuals has little effect (**Fig. 2a**).

Furthermore, we provide a theoretical model for the process of sorting long reads of Y chromosome (see Methods section for details). The core problem regarding the model is that there are N specific markers in a genomic region, and what is the probability that at least M markers are retained in the corresponding error-prone long reads. In addition to theoretical values, we used badread software to simulate the human T2T genome with 50X each of HiFi, Nanopore and PacBio CLR reads, and set a series of precision of MSK to assess the performance of SRY on sorting long reads. We find that the F1-score of SRY is larger than 90% even when the precision of MSK decreases to 70% (**Fig. 2b**). This is because the genome size of autosomes and X chromosome is about 3G, so the density of non-specific *k*-mers (non-MSK) derived from these chromosomes is small (1 *k*-mer/kb). The filter condition of SRY is ~7 *k*-mers per kilobase, so it is easy to filter out these non-Y chromosome sequences.

Comparison with the experimental method on real data

We collected datasets including short and long reads of a Chinese individual HX1[18, 19], and re-sequencing short reads of a Han Chinese population[20] to identify MSK (**Supplementary Table 1 and Table 2**). SRY obtained about 10 million MSK as well as sorted 3.7G (~46X) PacBio CLR and (~13X) ONT long reads of Y chromosome (**Supplementary Table 3**). We further collected Nanopore long reads (~2.3G, number of reads is 305,284) of an African human Y separated by flow sorting[8] and used minimap2[21] to align the sorted long reads from the two methods to the human T2T genome, separately. The results show that 94.0% of the sorted reads from SRY are mapped on T2T-Y chromosome, which is significantly higher than that of flow sorting (**Fig. 3a**). The human Y chromosome consists of several distinct regions (ampliconic, X-degenerate, X-transposed, pseudoautosomal, heterochromatic, others)[3]. We compared the performance of the two methods in these regions and found that SRY demonstrates comparable performance to flow sorting in the ampliconic, X-degenerate, X-transposed and others (**Fig. 3b**). However, SRY outperforms in terms of coverage and depth specifically in the heterochromatic region. (**Fig. 3b, c and Table 1**). The event of X-Y recombination is frequent on pseudoautosomal region (PAR, PAR1:1-2.8Mb, PAR2:56.9-57.2Mb). SRY aims to obtain Y-specific markers, hence the low coverage and shorter assembled result on PAR of SRY is expected (**Fig. 3b and Table 1**).

We further compared the resulting assemblies between the experimental and computational methods. SRY can achieve sort reads first and then assemble, or directly sort the assembled contigs based on MSK. The fast assembler wtdbg2[22] was used to assemble those sorted reads and flow sorting reads, and perform genome assemblies for trio binning and WGS (**Supplementary Table 3**). The total contig alignment length on T2T-Y chromosome from SRY is ~5.7Mb and ~9.6Mb longer than those from the sorted contigs of trio binning and WGS respectively. Moreover, the alignment lengths and the contiguity (NA50) on each discrete region of Y chromosome from SRY are all longer than the other two (**Table 1**), indicating that it is better to sort the reads first and then assemble them. Compared with flow sorting, there are less contamination from other chromosomes in the SRY assembly results (**Table 1**). Similarly to the result obtained from read sorting, the assembly result from SRY performed better in heterochromatic regions compared to flow sorting. However, in the PAR, the assembly result from SRY was inferior to those from flow sorting.

Towards complete genome assembly of Y chromosome

Mikko et al[23] developed an assembly software called Verkko, designed for HiFi and ultra-long Nanopore data, in order to achieve better automation of T2T-level chromosome assembly. Verkko demonstrated good result in the assembly of HG002. We sorted the Y chromosome data from HG002 and used Verkko for assembly. The results showed that, compared to Verkko with trio[23], the assembly of the sorted data (Verkko with SRY) reduced the number of contigs from 23 to 9 and corrected one assembly error (**Fig. 4**). Additionally, due to the high similarity of the X and Y chromosome PAR regions, Verkko trio's assembly result did not phasing this region well, resulting in two approximately 1Mb contigs aligning to the same region of the Y chromosome (**Fig. 4a**). Verkko SRY not only assembled this region completely, but also with higher accuracy (**Fig. 4b**). This indicates that SRY can further improve the performance of the assembly software with new sequencing technology.

Discussion

The development and evaluation of the SRY method for sorting long reads of sex-limited chromosomes have provided valuable insights into its effectiveness and potential applications. SRY has demonstrated its ability to identify MSK and successfully sort long sequences according to these markers. These separated long reads are then delivered to assemblers for genome assembly. Additionally, MSK can be used to select Y chromosome contigs from a whole genome assembly of a male individual . SRY provides an in silico sorting method that effectively identifies and sorts long reads of sex-limited chromosomes based on sex-specific markers.

However, our results can be influenced by read lengths and base pair qualities of reads. These factors indeed affect the accuracy of read mapping and assembly results.

Another consideration is that the accuracy of SRY-based sorting is influenced by the number of male and female individuals included. In contrast, flow sorting only requires one male individual of interest. However, SRY outperformed flow sorting in terms of mapping reads to the Y chromosome and demonstrated comparable or better performance in most regions, except for the pseudoautosomal region (PAR) where SRY had lower coverage and assembly results. Notably, to ensure a fair comparison with flow sorting, we limited our dataset collection to Nanopore or PacBio CLR data for the 10 individuals. This restricted dataset might have contributed to the suboptimal performance of SRY in the PAR region.

Nevertheless, it is worth mentioning that the incorporation of HiFi and ultra-long Nanopore data significantly improved the assembly quality of SRY in the PAR region. This suggests that the use of higher-quality sequencing data can greatly enhance the performance of SRY in assembling the challenging PAR region. The improved assembly results of SRY in the PAR region, along with its overall success in sorting and assembling long reads of sex-limited chromosomes, highlight its potential as a valuable alternative to experimental methods for studying sex-specific genomic regions.

Overall, our study demonstrates the effectiveness of SRY in sorting and assembling long reads of sex-limited chromosomes based on Y chromosome-specific markers. It is important to note that future research addressing W-chromosomes and accumulating relevant data in various species will be essential in expanding the scope and applicability of the SRY method to include sex-limited genomic regions.

Methods

SRY process

Firstly, SRY used kmer_count program to acquire *k*-mer (k=21)[12] sets from short reads of targeted male species and populations. Next, the program filterx (https://github.com/ruanjue/filterx) is used to identify specific *k*-mers associated with the male population. We labeled the *k*-mer files of all male individuals as "group1" and all female individuals as "group2". By comparing these groups and identifying *k*-mers as specific *k*-mers that are present in at least 2/3 of the individuals from group1. Then, SRY selects long reads of targeted species that have male specific *k*-mers (MSK). Finally, SRY filters those long reads with lower MSK densities than average value of whole Y chromosome.

False positive for MSK

False positive is introduced if a subsequence of length k (k-mer) originated from autosomes or X chromosomes is incorrectly identified as a MSK, with two possible sources: genomic coverage and population heterogeneity.

In the model, n males and n females are sequenced with the sequencing depth d. The length of a read is l and the sequencing error per site is r. C_n^i represents the number of ways to choose i elements from a set of n elements, also known as the binomial coefficient. In an individual, only the frequency of appearance of a k-mer that is more than once can we consider it to be present, and this event occurs with probability p_o. A k-mer is identified as an MSK if it is present in at least m males but not in any of the females. Particularly, we use m = 2/3n as the critical value by simulation study.

Let X be the frequency of appearance of a k-mer is present in an individual, then it follows a Possion distribution with rate $\lambda = d(1- r)^k(l - k + 1)/l$. It is easier to calculate $q_o = 1 - p_o$, which is the probability that a k-mer is absent, and thus

$q_o = P(X = 0) + P(X = 1) = e^{-\lambda} + \lambda e^{-\lambda} = (1 + \lambda)e^{-\lambda}$

We then can calculate the false positive rate caused by genomic coverage (f_1) by

$$f_1 = q_o^n \sum_{i=\lceil \frac{2}{3}n \rceil}^{n} C_n^i p_o^i q_o^{n-i} \tag{0.1}$$

If we set $r = 0.01$, $l = 150$, $d = 5$, $k = 21$ and $n = 5$, then f_1 is roughly 4.4×10^{-5}. If the sample size n increases to 10, then f_1 decreases to 1.9×10^{-9}, indicating that the false positive introduced by genomic coverage can be ignored if the sample size n is not too small.

For simplicity, we only consider heterogeneity in autosomes, and ignore heterogeneity in X chromosome. Assume one heterozygous site leads to two kinds of k-mers (k-mer-1 and k-mer-2), and the heterozygous proportions for two k-mers are p_{h1} and p_{h2} (with $p_{h1} + p_{h2} = 1$). If k-mer-j (j=1,2) from autosomes is observed in many males while it is not observed in any females, then k-mer-j is mistakenly identified as an MSK. As the discussion for the case of genomic coverage, the frequency of appearance of k-mer-j, X_j, follows Poisson distributions with parameters $\lambda_j = p_{hj}\,\lambda = p_{hj}\, d\,(1- r)^k(l - k + 1)/l$. As before, if a k-mer appears less than twice in an individual, we consider the k-mer is absent, and the probability of this event q_{oj} can be calculate as follows

$q_{oj} = P(X_j = 0) + P(X_j = 1) = e^{-\lambda j} + \lambda_j\, e^{-\lambda j} = (1+\lambda_j)\, e^{-\lambda j}$.

Given the heteropoietic rate h = 0.001, the probability that a k-mer with length 21 contains more than one heterozygous site is only 2.1×10^{-4}, which can be neglected. We also ignore the probability that k-mer-1 is identified as k-mer-2 mistakenly with edit distance 1 due to sequencing error, as such events occur with probability 0.99^20*r/3 = 0.3%. Then the false positive rate due to population heterogeneity f_2 can be calculated as follows:

$$f_2 = q_{o1}^n \sum_{\lceil i=\frac{2}{3}n \rceil}^{n} C_n^i p_{o1}^i q_{o1}^{n-i} + q_{o2}^n \sum_{\lceil i=\frac{2}{3}n \rceil}^{n} C_n^i p_{o2}^i q_{o2}^{n-i}. \tag{0.2}$$

The total false positive rate f is a weighted average of the false positive rate from the two sources, that is

$$f = (1 - kh)\, f_1 + khf_2 \tag{1.3}$$

Note that f_2 is actually a function of p_{hj}, which is an unknown parameter in the model. We further used the biallelic SNV datasets from 1000 genome project (http://ftp.1000genomes.ebi.ac.uk/vol1/ftp/data_collections/1000_genomes_project/release/20190312_biallelic_SNV_and_INDEL/) to estimate the empirical distribution of p_{h1}. We use a discrete distribution ranging from 0 to 0.5 to characterize the distribution of p_{h1}, which takes value of 0.05, 0.15, 0.25, 0.35, 0.45 with probability 92.77%, 2.59%, 1.80%, 1.48% and 1.36%, respectively. Combining the uncertainty of heterozygotic rate, the false positive rate is:

$$f = (1-kh)\, f_1 + kh\sum_x f_2(x)P(p_{h1} = x). \tag{0.3}$$

True positive rate (TPR) of identifying MSK

As discussed before, the probability of a k-mer present in more than two-thirds of male individuals is $\sum_{i=\frac{2}{3}n}^{n} C_n^i p_{oj}^i q_{oj}^{n-i}$. As the probability that a k-mer from autosomes is identified as a MSK due to sequencing error is too small, the probability that an MSK is present in none of the females is roughly 1. Then the TPR of identifying MSK is the product of the probability of the two events, that is

$$\text{TPR} = \sum_{i=\frac{2}{3}n}^{n} C_n^i p_{oj}^i q_{oj}^{n-i}$$

The probability of sorting long reads

For third-generation long reads, the sequencing errors are higher (PacBio CLR or Nanopore) and their lengths vary a lot. Assume a long read contains N specific markers, with N to be the function of the read length and the distribution of MSK, and the probability that a k-mer is correctly sequenced is $p = (1\text{-}r_3)^k$ (r_3 is the sequencing error of long reads). Then the probability that at least M MSKs are identified is

$$\sum_{i=M}^{N} C_N^i p^i (1-p)^{N-i}$$

In SRY software, the average number of MSKs (about 7/kb) across the Y chromosome is taken as the value of M to sort Y-chromosome long reads.

Assessment

In order to evaluate the MSK identification process of SRY, we firstly used kmer_count to obtain the *k*-mer of all chromosomes of the human T2T genome, and used filterx (https://github.com/ruanjue/filterx) to identify the specific *k*-mer of T2T-Y chromosome, which served as the standard for subsequent evaluations. Then, we used the mason_simulator (v2.0.9) program in the mason package (http://www.seqan.de/projects/mason/) with the parameter (--illumina-prob-mismatch 0.009 --illumina-prob-insert 0.0005 --illumina-prob-deletion 0.0005 --illumina-read-length 150) to generate short-read data for male and female populations using the human T2T genome with or without T2T-Y chromosome as a reference, respectively. We used different seed values for all individuals to avoid the result that the simulated data for all individuals were same. Finally, SRY used these population data to identify MSKs, which were evaluated by comparison with T2T-Y chromosome-specific *k*-mers.

For the theoretical value of the precision of SRY on identifying MSK, we used the following formula:

YSK*TPR/(YSK*TPR + AXK*FPR)

Where YSK represents the specific *k*-mer number of T2T-Y chromosome, AXK represents the *k*-mer number of the human T2T autosomes and X chromosome, and TPR represents the true positive rate and FPR represents the false positive rate of identifying MSK, respectively.

We further simulated 50X Nanopore, PacBio CLR and HiFi reads (25X for Y chromosome) based on the human T2T genomes using badread[24] package (v0.1.3) with the following commands, respectively:

badread simulate --reference human_autoX.fa --quantity 50X --error_model nanopore --start_adapter 0,0 --end_adapter 0,0 --junk_reads 0 --random_reads 0 --chimeras 0 (simulated Nanopore reads of autosomes and X chromosome)

badread simulate --reference human_Y.fa --quantity 25X --error_model nanopore --start_adapter 0,0 --end_adapter 0,0 --junk_reads 0 --random_reads 0 --chimeras 0 (simulated Nanopore reads of Y chromosome)

badread simulate --reference human_autoX.fa --quantity 50X --error_model pacbio --identity 85,95,3 --length 7500,7500 --start_adapter 0,0 --end_adapter 0,0 --junk_reads 0 --random_reads 0 --chimeras 0 (simulated PacBio CLR reads of autosomes and X chromosome)

badread simulate --reference human_Y.fa --quantity 25X --error_model pacbio --identity 85,95,3 --length 7500,7500 --start_adapter 0,0 --end_adapter 0,0 --junk_reads 0 --random_reads 0 --chimeras 0 (simulated PacBio CLR reads of Y chromosome)

badread simulate --reference human_autoX.fa --quantity 50x --error_model pacbio --qscore_model pacbio --identity 99,100,3 --length 12000,12000 --start_adapter 0,0 --end_adapter 0,0 --junk_reads 0 --random_reads 0 --chimeras 0 (simulated PacBio HiFi reads of autosomes and X chromosome)

badread simulate --reference human_Y.fa --quantity 25x --error_model pacbio --qscore_model pacbio --identity 99,100,3 --length 12000,12000 --start_adapter 0,0 --end_adapter 0,0 --junk_reads 0 --random_reads 0 --chimeras 0 (simulated PacBio HiFi reads of Y chromosome)

SRY was assessed for its ability to sort these simulated long reads by considering different TPR values of MSK.

Even the precision of MSK decreases to 70% (including ~7,000,000 MSKs and ~3,000,000 non-MSKs), the density of these non-MSKs on autosomes and X chromosomes was only 1 per kb, which was significantly lower than the threshold set by SRY (7/kb). Therefore, we took the theoretical

precision of SRY on sorting long reads as 1. For simplicity, the process of calculating the TPR of SRY on sorting long reads ignored the length distribution of the reads and used the window with 10kb length to calculate the specific *k*-mer distribution of the T2T-Y chromosome.

Y chromosome assembly, identification and evaluation

We collected ~60X ultra-long ONT data and ~35X HiFi data[23], and used SRY for the long-read sorting of the Y chromosome. Due to the abundance of repetitive sequences in the heterochromatic regions of the Y chromosome, the number of available Y-specific markers is limited. Therefore, we used two lengths of *k*-mers (*k*=21 and *k*=51) for the sorting of HiFi reads. Verkko (v1.0)[23] was used to assemble the selected data using default parameters. We compared the assembly results of Verkko in trio mode and SRY mode to T2T-CHM13 using the Quast (v5.0.2)[25] software. The file with the suffix name "coords.filtered" was used by DotPlotly (parameters: -slt -m 100 -q 100) (https://github.com/tpoorten/dotPlotly) to generate the alignment plot.

We collected the dataset of a trio family including short reads from father (HG01107, ~113X) and mother (HG01108, ~79X) and Nanopore reads from child (HG01109, ~72X)[26]. SRY separated 1.3G (~25X) long reads of HG01109 using MSK markers identified from HX1. Then, we used wtdbg2.5[22] with parameters “-L 0 -p 0 -k 21 -s 0.25 -S 2 --rescue-low-cov-edges” to assembly those long reads. The remaining long reads were assembled by wtdbg2.5 with parameters “-x ont -g 3g” and polished with the program wtpoa-cns in wtdbg2.5. All of the assembled contigs were further polished with wtpoa-cns using short reads. Sorting of long reads and genome assemblies for other nine individuals were performed the same way. Trio binning phased HG01109 long reads using with command “canu -stopAfter=haplotype genomeSize=3g -haplotypeMale HG01107.fastq.gz -haplotypeFemale HG01108.fastq.gz -nanopore-raw HG01109.fasta.tar.bz2”. Wtdbg2 with the parameters (-g 3.1G -x ont) was applied to assembly phasing reads from trio binning and perform whole genome assembly for WGS. SRY was then used to partition candidate contigs of Y chromosome for trio binning and WGS. We utilized quast[25] (v5.0.2) with default parameters to evaluate the assembled genome quality.

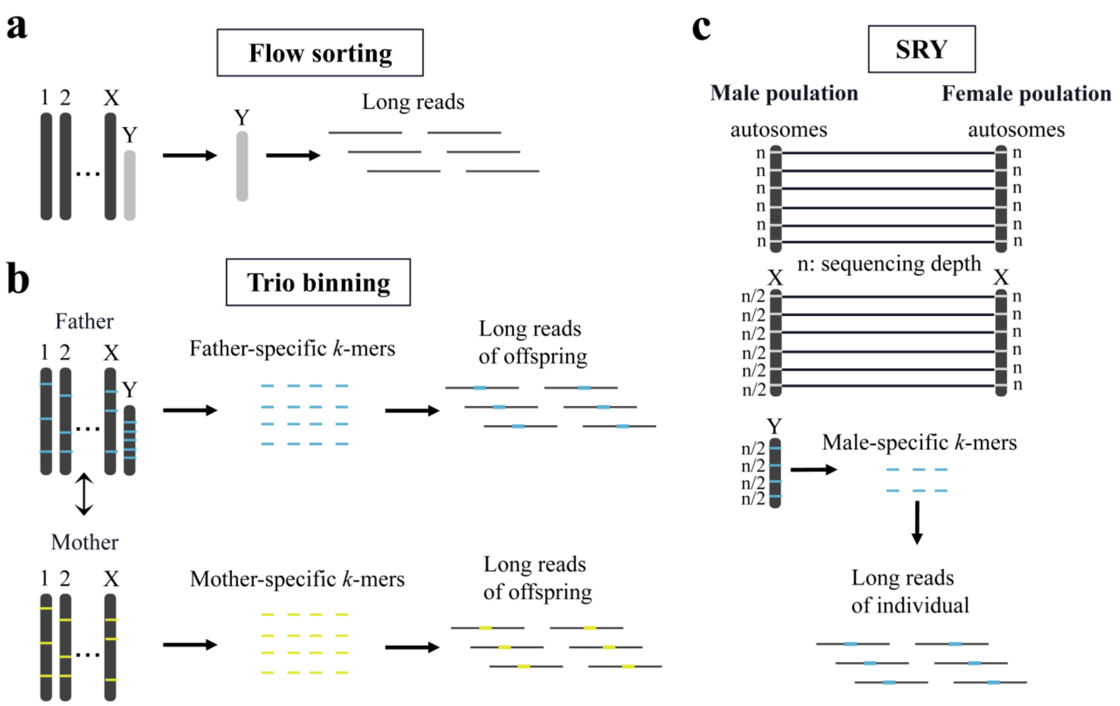

Fig. 1: Overview of three methods for sorting long reads. a Flow-sorting is an experimental method for separating Y chromosome. **b** Trio binning compares *k*-mers from short reads of parent genomes and identifies father-specific and mother-specific *k*-mers, respectively. These specific *k*-mers are used to bite long reads for each parent. **c** SRY rules out *k*-mers presenting in both male and female populations and retains *k*-mers only occurred in male population with half sequencing depth. SRY utilizes these male-specific *k*-mers to separate long reads of Y chromosome.

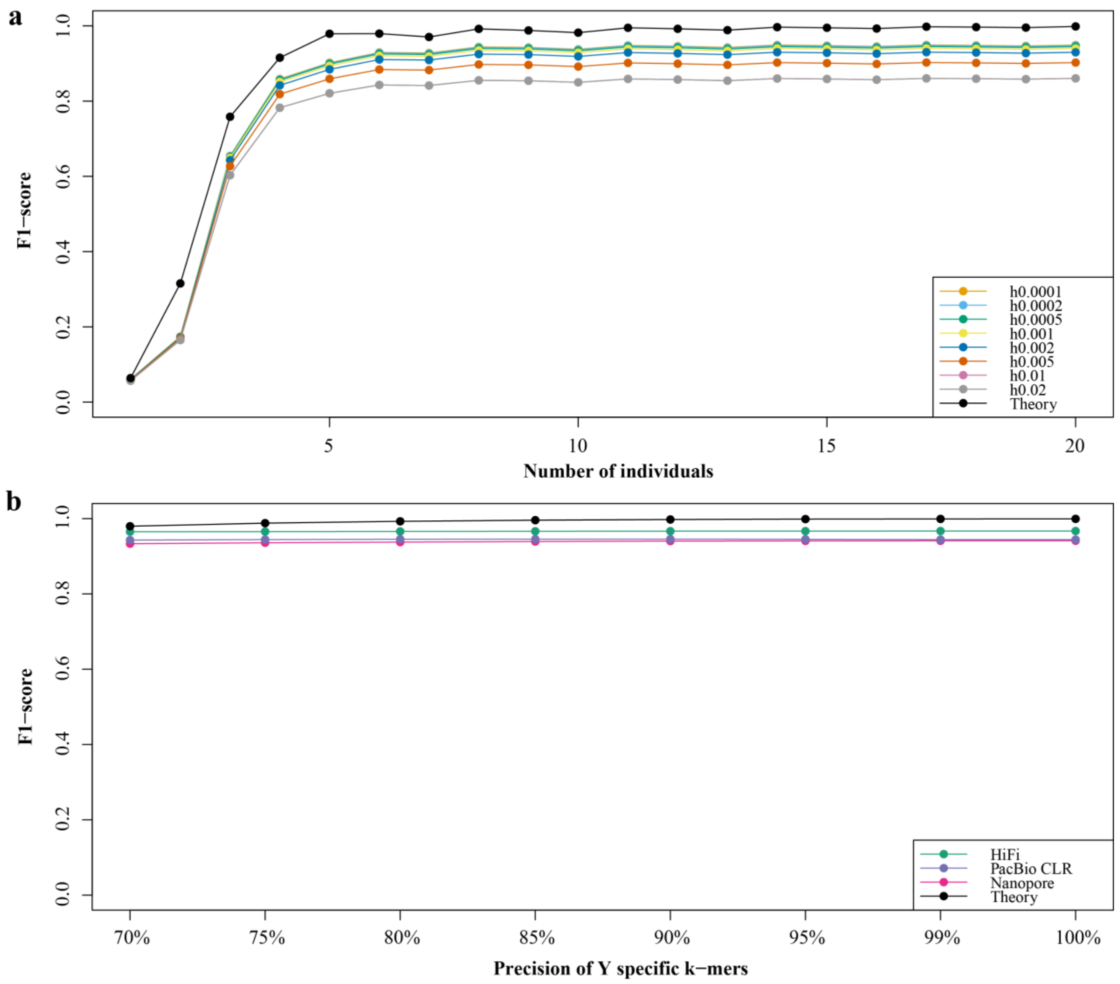

Fig. 2: Theoretical model and performance of SRY on simulated data. a The F1-score of SRY on identifying male-specific *k*-mers with different individual number (per sex) and population heterogeneity, respectively. For simplicity, we did not consider the similarity distribution between the Y chromosome and other chromosomes in the calculation of the theoretical value. **b** The F1-score of SRY on sorting HiFi, PacBio CLR and Nanopore long reads. The combination of length distribution and specific *k*-mers distribution can cause TPR differences between theoretical and simulated data as well as TPR differences within simulated data.

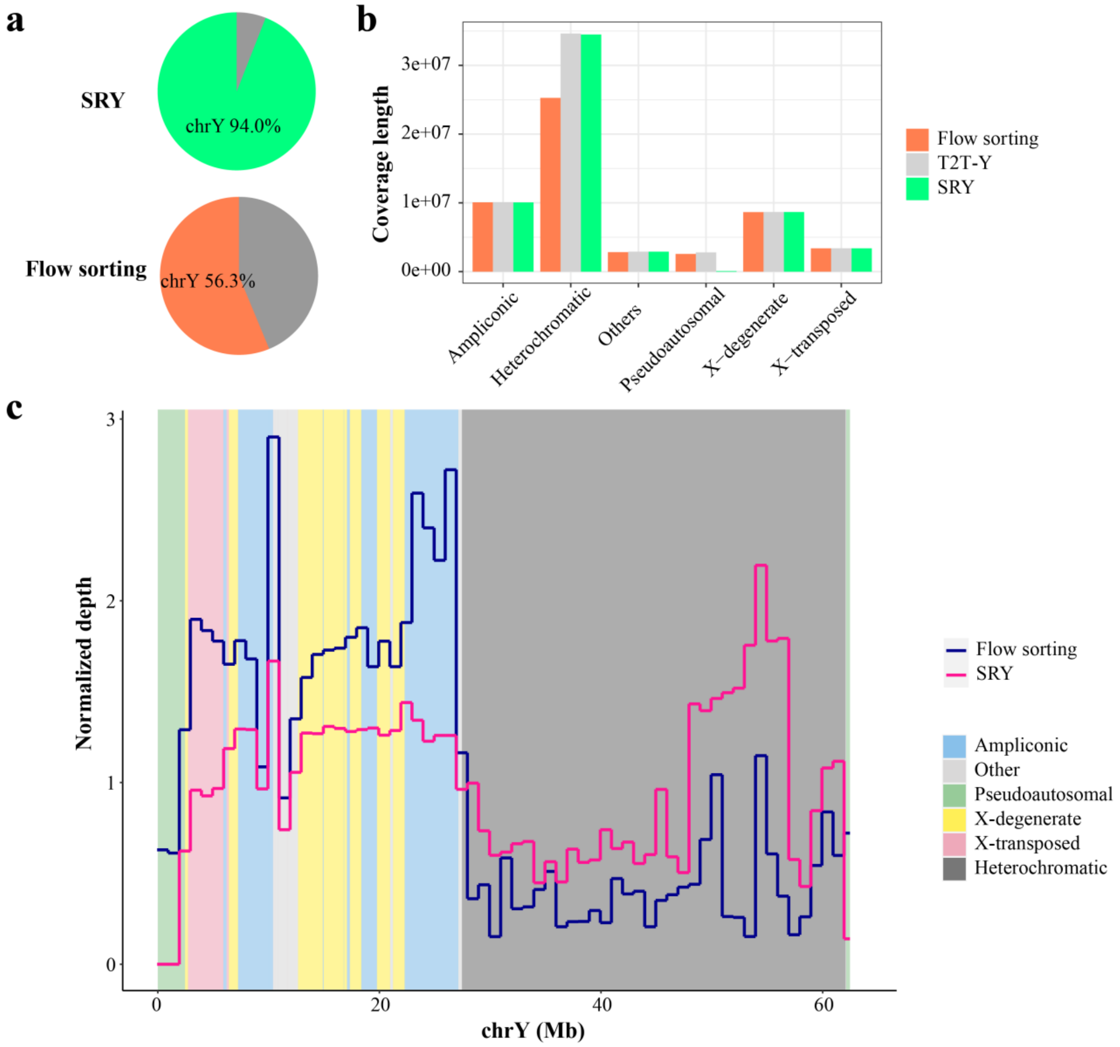

Fig. 3: Comparison of sorting results between SRY and flow sorting. a Alignment distribution of sorted reads for SRY and flow sorting. The alignment on autosomes and X chromosome is colored in gray. **b** The coverage of sorting reads by the two methods on discrete regions of Y chromosome. SRY aims to separate male-specific long reads, so the coverage is lower on pseudoautosomal region where recombination events occur frequently between X and Y chromosome. **c** The normalized depth of long reads separated by the two methods. The colored rectangles represent discrete regions on the T2T-Y chromosome.

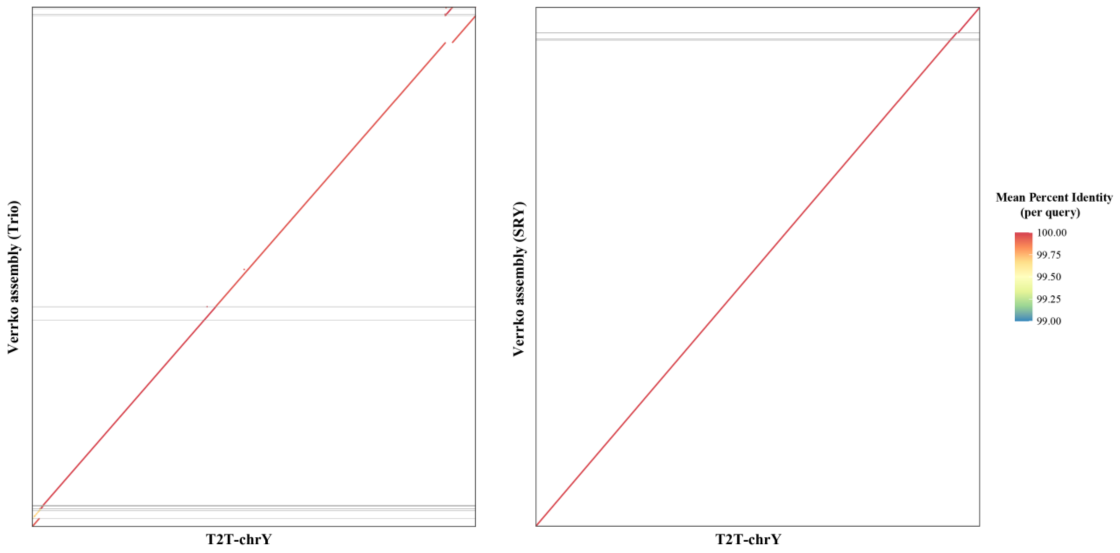

Fig. 4: Verkko assembly using Trio or SRY. The x axis represents the complete Y chromosome assembled by T2T consortium, and y axes represent assemblies by verkko with Trio (left) and SRY (right), respectively. The diagonal colored with identity shows the alignment result.

		Experimental	Computational			
		Flow_sorting (HG02982)	SRY read-sorting+ WTDBG2 assembly		Trio_binning read-sorting +WTDBG2 assembly+ SRY contig-sorting (HG01109)	WTDBG2 assembly+ SRY contig-sorting (HG01109)
			HG01109	Pan-Y		
Depth of sorted reads		~45X	~25X	~16-71X	-	-
All	Total length	22,000,118	22,866,849	17,705,259-22,866,849	15,161,111	14,591,512
	N50	1,572,692	1,529,669	1,418,447-2,333,095	1,208,828	507,517
	Autosome+X aligned length	1,846,274 (8.4%)	497,792 (2.2%)	246,997-578,565 (1.2%-2.9%)	288,974 (2.0%)	2,737 (0.0%)
	Y aligned length	18,577,804	19,713,509	16,737,249-20,388,526	14,058,622	10,127,949
	NA50	710,714	711,640	606,225-1,138,334	501,025	290,040
	NA75	104,485	42,458	42,458-260,602	197,270	166,322
	Mismatches (/100kbp)	263.80	267.55	175.56-294.71	333.66	160.19
	Indels (/100kbp)	189.54	194.58	109.41-221.67	316.04	150.95
Ampliconic	Aligned length	6,471,932	6,332,466	4,755,420-6,332,466	3,747,647	3,986,246
	Mismatches (/100kbp)	358.03	497.48	372.61-546.87	743.74	474.86
	Indels (/100kbp)	165.60	229.55	84.39-229.55	314.39	215.17
X-degenerate	Aligned length	8,884,024	8,810,648	8,283,581-8,883,474	7,715,598	6,378,403
	Mismatches (/100kbp)	164.47	152.84	124.91-161.83	327.39	149.71
	Indels (/100kbp)	78.46	87.7	41.79-132.41	291.2	112.38
X-transposed	Aligned length	3,880,237	3,862,338	3,675,209-3,862,338	3,579,267	356,321
	Mismatches (/100kbp)	623.08	602.77	497.26-602.77	807.24	24,596.98
	Indels (/100kbp)	112.86	166.23	56.35-184.35	387.48	586.9
Pseudoautosomal	Aligned length	2,711,039	640,462	494,367-640,462	413,077	319,043
	Mismatches (/100kbp)	1,414.61	14,258.98	13,728.13-29,498.05	28,196.73	44,693.71
	Indels (/100kbp)	259.42	850.6	850.6-1,800.38	1,062.47	1,287.86
Heterochromatic	Aligned length	70,190	1,210,886	243,487-2,607,300	7,892	7,030
	Mismatches (/100kbp)	3,360.46	4,705.24	1,993.89-4,705.24	11,031.86	11,479.59
	Indels (/100kbp)	830.67	1,465.1	562.99-1,961.29	713.27	561.22
Others	Aligned length	1,386,458	1,490,295	1,300,738-1,777,989	396,649	335,380
	Mismatches (/100kbp)	2,104.37	2,154.25	1,891.85-2,529.37	14,215.11	14,183.14
	Indels (/100kbp)	238.67	462.89	462.89-471.89	773.11	591.64

Table 1 Comparison of the assembled genomes for SRY and flow sorting. Human sample numbers are in parentheses, and the sample numbers and metrics of Pan-Y are detailed in supplementary table 4.

Reference

1. Tomaszkiewicz, M., Medvedev, P. & Makova, K.D. Y and W Chromosome Assemblies: Approaches and Discoveries. *Trends. Genet.* **33**, 266-282 (2017).
2. Bellott, D.W. et al. Avian W and mammalian Y chromosomes convergently retained dosage-sensitive regulators. *Nat. Genet.* **49**, 387-394 (2017).
3. Skaletsky, H. et al. The male-specific region of the human Y chromosome is a mosaic of discrete sequence classes. *Nature* **423**, 825 (2003).
4. Hughes, J.F. et al. Chimpanzee and human Y chromosomes are remarkably divergent in structure and gene content. *Nature* **463**, 536-539 (2010).
5. Hughes, J.F. et al. Strict evolutionary conservation followed rapid gene loss on human and rhesus Y chromosomes. *Nature* **483**, 82-86 (2012).
6. Soh, Y.Q.S. et al. Sequencing the Mouse Y Chromosome Reveals Convergent Gene Acquisition and Amplification on Both Sex Chromosomes. *Cell* **159**, 800-813 (2014).
7. Dolezel, J. et al. Chromosomes in the flow to simplify genome analysis. *Funct. Integr. Genomics* **12**, 397-416 (2012).
8. Kuderna, L.F.K. et al. Selective single molecule sequencing and assembly of a human Y chromosome of African origin. *Nat. Commun.* **10**, 4 (2019).
9. Akagi, T., Henry, I.M., Tao, R. & Comai, L. A Y-chromosome–encoded small RNA acts as a sex determinant in persimmons. *Science* **346**, 646-650 (2014).
10. Carvalho, A.B. & Clark, A.G. Efficient identification of Y chromosome sequences in the human andDrosophilagenomes. *Genome Res.* **23**, 1894-1907 (2013).
11. Muyle, A. et al. SEX-DETector: a probabilistic approach to study sex chromosomes in non-model organisms. *Genome Bio. Evol.* **8**, 2530-2543 (2016).
12. Koren, S. et al. De novo assembly of haplotype-resolved genomes with trio binning. *Nat. Biotechnol.* **36**, 1174-1182 (2018).
13. Tomaszkiewicz, M. et al. A time- and cost-effective strategy to sequence mammalian Y Chromosomes: an application to the de novo assembly of gorilla Y. *Genome Res.* **26**, 530-540 (2016).
14. Nurk, S. et al. The complete sequence of a human genome. *Science* **376**, 44-53 (2022).
15. Rangavittal, S. et al. DiscoverY: a classifier for identifying Y chromosome sequences in male assemblies. *BMC genomics* **20**, 1-11 (2019).
16. Rangavittal, S. et al. RecoverY: k-mer-based read classification for Y-chromosome-specific sequencing and assembly. *Bioinformatics* **34**, 1125-1131 (2018).
17. Hall, A. B. et al. Six novel Y chromosome genes in Anophelesmosquitoes discovered by independently sequencing males and females. *BMC genomics* **14**, 1-13 (2013).
18. Shi, L. et al. Long-read sequencing and de novo assembly of a Chinese genome. *Nat. Commun.* **7** (2016).
19. Liu, Q. et al. Detection of DNA base modifications by deep recurrent neural network on Oxford Nanopore sequencing data. *Nat. Commun.* **10**, 2449 (2019).
20. Lan, T. et al. Deep whole-genome sequencing of 90 Han Chinese genomes. *GigaScience* **6** (2017).
21. Li, H. Minimap2: pairwise alignment for nucleotide sequences. *Bioinformatics* **34**, 3094-3100 (2018).
22. Ruan, J. & Li, H. Fast and accurate long-read assembly with wtdbg2. *Nat. Methods* **17**, 155-

158 (2020).
23. Rautiainen, M. et al. Telomere-to-telomere assembly of diploid chromosomes with Verkko. *Nat. Biotechnol*. https://doi.org/10.1038/s41587-023-01662-6 (2020).
24. Wick, R. R. Badread: simulation of error-prone long reads. *J. of Open Source Softw.* **4**, 1316 (2019).
25. Gurevich, A., Saveliev, V., Vyahhi, N. & Tesler, G. QUAST: quality assessment tool for genome assemblies. *Bioinformatics* **29**, 1072-1075 (2013).
26. Shafin, K. et al. Nanopore sequencing and the Shasta toolkit enable efficient de novo assembly of eleven human genomes. *Nat. Biotechnol.* (2020).

Data availability

We downloaded all Nanopore, PacBio and Illumina datasets under NCBI project number PRJNA301527 for HX1. The SRA numbers of Han Chinese population were listed at supplementary table 1 and 2. The trio family (HG01107, HG01108 and HG01109), HG005, HG006, HG01243, HG02055, HG03098 and HG03492 reads are available at https://s3-us-west-2.amazonaws.com/human-pangenomics/index.html. Short reads as well as PacBio and/or Nanopore long reads of HG002 and HG003 are available at https://ftp-trace.ncbi.nlm.nih.gov/ReferenceSamples/giab/data/AshkenazimTrio/. We also downloaded ERR3241824 for HG01107 and ERR3241825 for HG01108 to improve the performance of trio binning. All the assembling results have been submitted to figshare (https://doi.org/10.6084/m9.figshare.14564484).

Code availability

The SRY source code is hosted by GitHub at: https://github.com/caaswxb/SRY.

Acknowledgments

This work was supported by National Natural Science Foundation of China (Grant No. 91731304 to J.R.), National Key Research and Development Program of China (Grant No. 2019YFA0707003 to J.R.) and National Natural Science Foundation of China (Grant No. 31860638 to Q. L.). We thank S. Wu from CAAS for his suggestions on genome assembly. We thank High-performance Computing Center of Agricultural Genomics Institute at Shenzhen, China Academy of Agricultural Sciences.

Author contributions

J.R. and Q. L. designed the project, and J.R. managed the project. X.W., J.R. and A.L. developed the SRY method. T.Z. constructed the theory model. X.W. and H.L. collected genomic data, performed analysis and wrote the paper. J.R., H.Z. and Y.Z. revised the manuscript.

Competing interests

The authors declare no competing interests.

Figure4

Click here to access/download;Figure;figure4.pdf

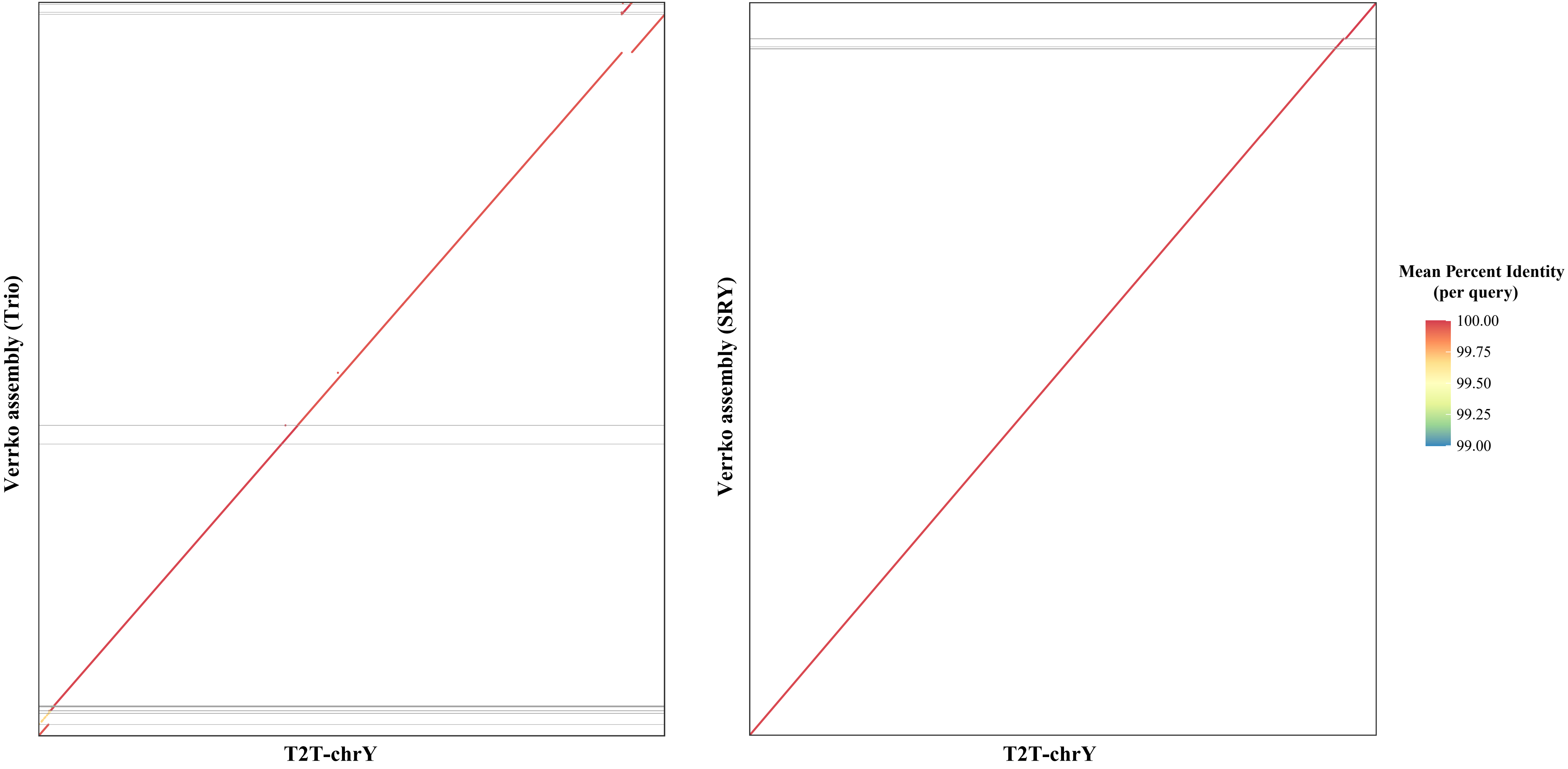

Figure3

Click here to access/download;Figure;figure3.pdf

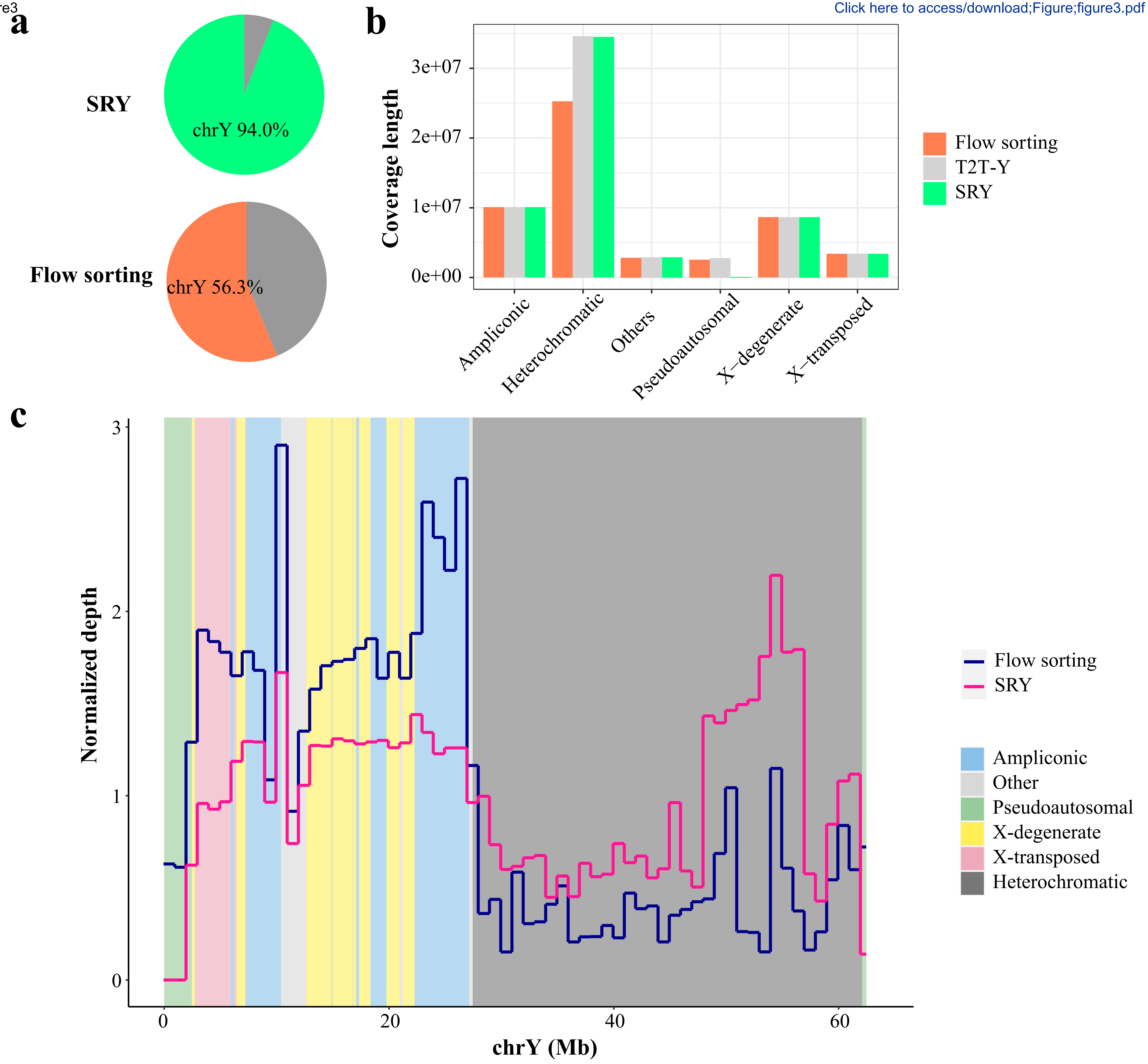

Figure2

Click here to access/download;Figure;figure2.pdf

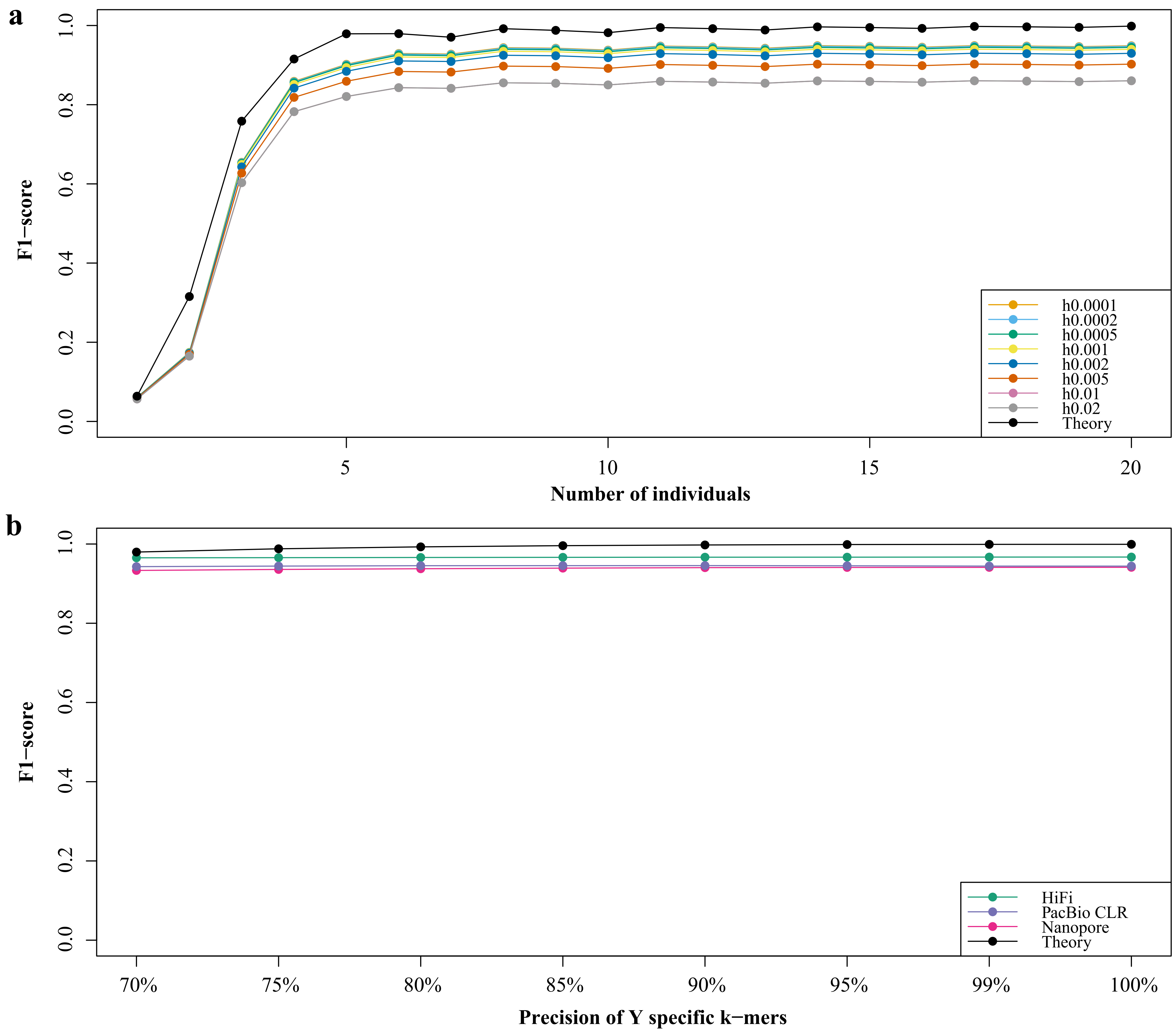

Figure1

Click here to access/download;Figure;figure1.pdf

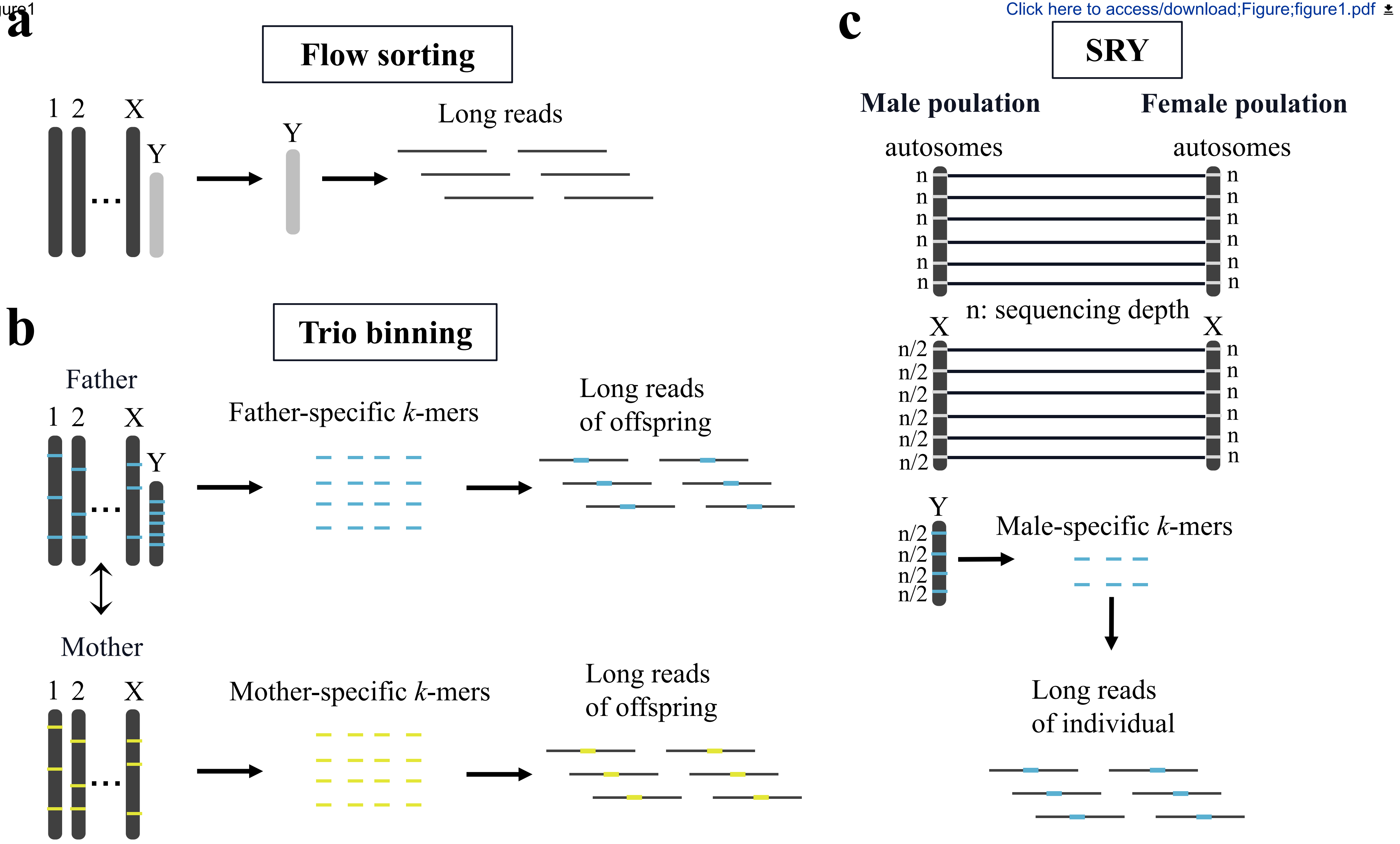

Click here to access/download
Supplementary Material
Supplemental-20230731.docx

Click here to access/download;Personal Cover;CV-20230731.docx

Aug. 1th, 2023

Dear Editor,

I hope this letter finds you well. I am pleased to submit a revised version of our manuscript entitled "**An effective strategy for assembling the sex-limited chromosome**", which we believe is suitable for publication in *GigaScience*. This resubmission follows the thorough review process and constructive feedback received from the two reviewers at Nature Communications.

In response to the reviewers' suggestions, we have made substantial revisions to the manuscript, addressing their concerns and incorporating their valuable suggestions. In accordance with the recommendation of Reviewer 1, we have performed additional analyses comparing our computational method SRY with the verkko trio model. This comparison, based on the human T2T reference genome, allows us to highlight the superiority of our SRY approach. We have included these comparative analyses in the revised manuscript, and the results clearly demonstrate that SRY significantly enhances the assembly results of the Y chromosome when hifi and ultralong ONT reads are utilized.

The revised manuscript now provides a more comprehensive assessment of the performance and the broader implications of our SRY method. The inclusion of the additional analyses and the overall improvements made following reviewer feedback have significantly enhanced the manuscript's scientific rigor and overall impact.

In light of these revisions, we kindly request the consideration of our manuscript for publication in *GigaScience*. We believe that our study addresses an important gap in current reference genomes by presenting a computational method that enables accurate assembly and further research on sex-limited chromosomes. The findings have significant implications for the field and are of broad interest to both geneticists and computational biologists.

Thank you for considering our revised manuscript. We appreciate your time and attention to our work and look forward to hearing from you soon. Please feel free to contact us if you require any further information or clarification.

Sincerely,

Jue Ruan
PH.D
Agricultural Genomics Institute
Chinese Academy of Agricultural Sciences
Shenzhen, Guangdong 518120, China
Email: ruanjue@caas.cn

Tel: +86-18513511304
Fax: +86-755-23250365

Response to Reviewers

Reviewer #1 (Remarks to the Author):

The authors developed a k-mer based method, SRY, that selects Y-specific markers from population of homogametic (XX or ZZ) and heterogametic (XY or ZW) genome data. The male specific k-mers (tested in human, MSK) are obtained from the single-copy region k-mers, by subtracting all k-mers seen in the homogametic individuals. Then, reads are binned to the Y chromosome depending on the MSK density for further assembling the Y.

The idea for using homogametic vs. heterogametic populations to obtain heterogametic sex-limited markers was around, however to the best of my knowledge, never been described nor tested extensively for assembling sex-limited chromosomes de novo, without the use of a prior reference (or candidate reference) genome.

I have numerous comments and concerns to raise on the benchmark the authors performed. Even if the concerns are addressed, I would rather be pessimistic that this method would be widely adapted. The methods used for producing the first Telomere-to-Telomere genome is now automated and available as Verkko, which produces phased, diploid assemblies of both haplotypes. Most of the Y, except the PAR, are separable with PacBio HiFi reads. ONT ultra-long reads were vital for resolving palindromes and heterochromatic region of the Y. The Y assembly results from the SRY authors failed to assemble the PAR, and were far from getting the Y chromosome complete. The authors also admit that their pan-genome Ys have collapses. In addition, there are no data showing that the ampliconic sequences, palindromes, or inverted repeats are correctly assembled. Coverage is not enough. The Y has been known to have polymorphic inversions and ampliconic duplications, which some are shared among particular Y haplogroup lineage. This makes the Y unique (and interesting) compared to the other chromosomes. What would be the use of the SRY assemblies? I worry that such collapsed, error-prone assemblies would mis-lead the interpretation of the Y chromosome (or any other sex-limited chromosome) studies.

Response: Thank you for your insightful comments and concerns regarding the benchmark performed in our study. We appreciate your skepticism regarding the wide adaptation of our method. We would like to address your points.We acknowledge that Verkko, an automated phased assembly method, has been successful in producing Telomere-to-Telomere genomes. However, we found that sorting HiFi and ultra-long Nanopore data by SRY yielded better results compared to Verkko's trio mode. Our aim was to explore alternative approaches and compare them with the flow sorting method used by Kuderna et al. In order to ensure a fair comparison, we performed sorting on Nanopore or PacBio data from 10 individuals. These 10 individuals were not chosen to represent a diverse population, but rather to provide a consistent dataset for comparison purposes. We did not intend for these assemblies to be comprehensive representations of the Y chromosome in these individuals. To address your concerns, we have made revisions to the manuscript. We have emphasized the benefits of sorting HiFi and ultra-long Nanopore data over Verkko's trio mode. We have also removed the analysis of the pan-genome Ys to align with your viewpoint.

One other comment regarding the SRY method is that the Y chromosome harbors the most number of paternal-specific, multi-copy k-mers. As such, limiting the MSK set to single-copy region would result in large fractions of missing k-mers that belong to the Y. Amplicons, palindromes are full of duplicated k-mers. The Yqh, the large heterochromatin block consists of k-mers with much higher frequency. Eliminating all those k-mers explain why the SRY assemblies have more collapses.
Response: We apologize for any confusion caused by our previous response. It seems that our explanation was not clear enough. We would like to clarify that our sorting approach did not solely focus on single-copy regions. Instead, we targeted the entire Y chromosome to identify MSK. We observed that the use of SRY-based sorting on ultra-long Nanopore and HiFi data led to improved assembly results for the Y chromosome compared to Verkko's trio mode. This finding suggests that our method has the potential to enhance Y chromosome assembly.

Below are specific comments and concerns that needs to be addressed properly.
Line 32-33 "Generally, most genome sequencing projects prefer homogametic (XX females or ZZ males) to heterogametic (XY males or ZW females) genomes,":
I would argue that this is not true anymore. It has been historically preferred to assemble homogametic genomes. Using the parental genomes, trio binning enables complete chromosome phasing, and as such has been preferred for obtaining both sex chromosomes in heterogametic genomes. In fact, trio-binning works better on more diverged (highly heterozygous) genomes, such as the F1 hybrids from sub-species cross.
Response: We acknowledge that the term "generally" may not accurately reflect the current state of genome sequencing projects. In line with your suggestion, we have revised the statement to "traditionally" to better convey the historical preference for assembling homogametic genomes.

Line 35-36 "Besides, as XY or ZW chromosomes were evolved from a pair of autosomes":
That evolution happened very long time ago, enough to make the XY or ZW diverged. The PAR are the only pieces that still frequently recombines. X-transposed regions have divergence of ~97%, which is easily separable using HiFi reads.
Response: We fully agree with your observation that during the sorting of HiFi reads by SRY, the coverage for the non-PAR regions is generally satisfactory. The sentence has been corrected to "While XY or ZW chromosomes have diverged significantly from their ancestral autosomes, their homology can still pose challenges for genome assembly. Homologous regions, such as the pseudoautosomal regions (PAR), can lead to fragmented contigs similar to large repeats. ".

Line 40 "BAC-based method, was once successfully applied on deciphering the Y chromosomes":
I disagree that they were "successful". If so, why did the human Y have over 50% of gaps remained? The initial Y paper (Skaletsky et al. 2003) admits that they could only able to span the boundaries, with the distal Yq end not identified with certainty: "We attempted to sequence BACs spanning the boundaries and representing the body of each of the three heterochromatic blocks. We succeeded, with the exception that the distal boundary of the major heterochromatic region, on distal Yq, was not identified with certainty."
Response: Thank you for bringing it to my attention. The word "successfully" has been removed.

Line 50 “population heterozygosity”: I wonder “population heterogeneity” would be a better usage?
Response: Thank you for the suggestion. We have replaced "population heterozygosity" with "population heterogeneity".

Line 56 “Thanks to the increasing sequencing read length”: Both the read length and higher accuracy played a major role to the success of the T2T assemblies.
Response: Thank you for pointing that out. We have made the modification to reflect that both the read length and higher accuracy played a major role in the success of the T2T assemblies.

Line 60 “sexPhase program”: Is this a tool? There was no such program used for assembling the T2T-HG002 X and Y. For the T2T XY, haplotype specific markers from homopolymer compressed, trio-binned k-mers were used to walk through the graph to resolve the PAR. The rest of the X and Y portion were resolved automatically at the HiFi resolution, thanks to the highly diverse X and Y sequences.
Response: sexPhase (https://github.com/tangerzhang/sexPhase) is a software specifically developed for analyzing the dioecious plant species, Ficus.

Line 62 “Recently, trio binning …”: I’d suggest to switch the order with T2T Y. Trio binning predates the HiFi reads and was designed to work best on noisy long-reads.
Response: Thank you for your suggestion. We have made the modification to switch the order.

Line 65 “HG01109”: Was there a context here? This seems not relevant and should be removed.
Response: Removed.

Line 99 “one is coverage and the other is heterozygosity”: what is the heterozygosity here? The difference between X and Y? Or the heterogeneity among the XY individuals (specifically, divergence between the Ys among these XY individuals)?
Response: .the term "heterozygosity" refers to population heterogeneity, not the difference between X and Y chromosomes or the diversity among the Y chromosomes among XY individuals. It pertains to the variation or diversity within a population. I apologize for the confusion caused by the previous use of "heterozygosity." It has been revised to "heterogeneity" in the manuscript to better reflect the intended meaning.

Line 101 “we used the mason_simulator software”: The T2T Y was released earlier this year (April). Why is the simulation performed on the incomplete GRCh38Y? Along this line, I would argue that all the benchmarks performed on GRCh38Y should be performed on the T2T-Y.
Response: You make a valid point. We apologize for the oversight in using the incomplete GRCh38Y reference genome for the simulation. We agree that it would be more appropriate to perform the simulation using the complete and more recent T2T-Y reference genome. We have revised our methodology to use the T2T-Y reference genome for all the benchmarks and analyses in the study. This change ensures that the evaluation is done on the most up-to-date and comprehensive reference genome available.

Line 108 “PacBio reads”: PacBio HiFi reads or CLR reads? It is important which read dataset the SRY is designed for. HiFi and CLR have very different error models.
Response: Apologies for the confusion.The term "PacBio reads" in line 108 (actually 118) refers specifically to PacBio CLR (Continuous Long Read) reads. However, we have also developed a separate module in our software that handles PacBio HiFi (High Fidelity) reads. We have included simulations using HiFi data to evaluate the performance of the SRY on HiFi reads.

Lines 127-128 “This is because longer reads are more likely to have more specific k-mers and a higher density of specific k-mers.”: Error rates in the reads should be accounted as well.
Response: The error rates in the reads should indeed be considered as a factor that can affect the efficiency of target enrichment. Additionally, the distribution of specific k-mers can also impact the performance.Upon further consideration and with the switch to the T2T-Y reference, we recognize that the conclusion in lines 127-128 is not accurate. It is clear that multiple factors, including read length, error rates and k-mer distribution, can have a combined impact on the efficiency of target enrichment. Therefore, we have removed this inference from the manuscript.

Line 130-, Comparison with other methods on real data section: As mentioned above, the entire section needs the comparison and evaluation be performed on T2T-Y.
Response: Thank you for your feedback. We have now replaced the comparison and evaluation with the results obtained using T2T-Y.

Line 134 “(71X) PacBio and ONT long reads”: Again, be specific on what coverage was used in what dataset. For example, “(~X) PacBio CLR reads and (~X) ONT long reads”.
Response: I have made the revision based on your suggestion. The line now reads as follows: "(~46X) PacBio CLR and (~13X) ONT long reads".

Line 135 “HG01109”: Is there a known Y lineage for this genome? If so, indicate it here.
Response: Thank you for your inquiry. There is no known Y lineage for the genome HG01109.

Line 136 “African human Y separated by flow sorting”: This is HG02982, A0 lineage. I am not convinced comparing two different Y lineages are fair, as one is more closer to the GRCh38Y. Besides, do we really need to benchmark against the flow-sorting based method? This would be my least interest of the benchmark. I haven’t seen any recent large-scale genome assembly projects performed based on flow-sorting. The better benchmark, and interest for the readers, would be the comparison on HiFi reads, against the trio-binned Verkko or hifiasm assemblies. If the application of SRY is targeting non-HiFi based assemblies, it should be specified.
Response: Thank you for your comments and suggestions. We understand your concern regarding the comparison of different Y lineages. However, it is important to note that currently there are no available computational methods specifically designed for sorting sex-specific chromosome third-generation sequencing data. In our study, the flow-sorting method is the only approach that provides third-generation sequencing data for comparison. While we acknowledge that large-scale genome assembly projects based on flow-sorting are not common, it is still valuable to assess the performance of SRY selection using this method as it represents an existing approach for sex-

specific chromosome sequencing. Based on your suggestion, we have included a comparison with the trio-binned Verkko assemblies using ultra-long ONT and HiFi reads. We found that the application of SRY sorting followed by assembly using the Verkko method yielded better results.

Line 138 “84% of the sorted reads from SRY are mapped on Y chromosome”: Where do the 16% go? I believe, again, the T2T-CHM13 would provide a better understanding for the MSK marker efficiency.
Response: We agree with the reviewer's suggestion to use T2T-CHM13 as a reference to further investigate the marker efficiency of MSK. After aligning the sorted reads from SRY to the T2T-CHM13 reference, we found that 94% of the SRY sorted reads were mapped to the Y chromosome. As discussed earlier, the remaining 6% of the Y chromosome reads that were not sorted by SRY could be attributed to factors such as read length, sequencing errors, and specific k-mer distribution patterns. These factors may have affected the ability of SRY to accurately identify and sort all Y chromosome reads.

Line 139 “one times higher”: one times means “equal”. Correct this. Did you mean “Two times higher”?
Response: Corrected.

Line 165- : Again, the entire section needs to be performed on T2T-Y.
Response: We have performed the entire section on T2T-Y as requested.

Lines 166-172: Most medically relevant genes on the Y are the ampliconic, multi-copy genes. Having those collapsed is a poor indicator. Besides, what about other types of misassemblies? Structural accuracy and base accuracy have never been mentioned.
Response: We would like to highlight that as discussed earlier, the assembly results from the 10 individuals were solely used for comparison with flow sorting. The analysis of the pan-Y genome has been removed from the study. Additionally, we have included a comparison with the verkko trio model to provide a more comprehensive evaluation.

Line 178 “which maybe result from the loss of function of heterochromatin sequences”: No, this is not true. The heterochromatic region here in GRCh38Y include the centromere, DYZ17, DYZ19, and the large Yqh block. The centromere in the GRCh38Y is infect a model sequence, obtained from RP11 cell line (R1b Y haplogroup). DYZ17 and DYZ19 has been mis-assembled in GRCh38Y. The concentrated SNPs are reflecting this complicated mis-match of Y haplogroup, mis-assembly, and collapses in GRCh38Y and the assembled Ys.
Response: We apologize for the confusion caused. We have removed this section from the manuscript, in line with our previous response.

Line 181: Correct gramma
Response: Corrected.

Line 186-189: It is expected to have a lot of re-arrangements compared to any Y reference. I would again disagree that showing the collinearity with GRCh38Y means “high quality”. A

thorough evaluation is required for the large SVs on the Y, to proof the assembly is in high quality before drawing any conclusion. This goes in line with the ~300 SVs found per individual on average. It should be checked that those 300 does not include assembly artefacts, such as mis-assemblies (which could frequently happen in palindromes), collapses or false duplications.

Response: As previously mentioned, the portion of Pan-Y analysis including this result has been removed from the manuscript.

Fig. 3: I'd be interested to see this on the T2T-Y, not on the collapsed and gapped GRCh38Y.

Response: We have already completed the analysis using the T2T-Y as a reference and have updated Figure 3 accordingly.

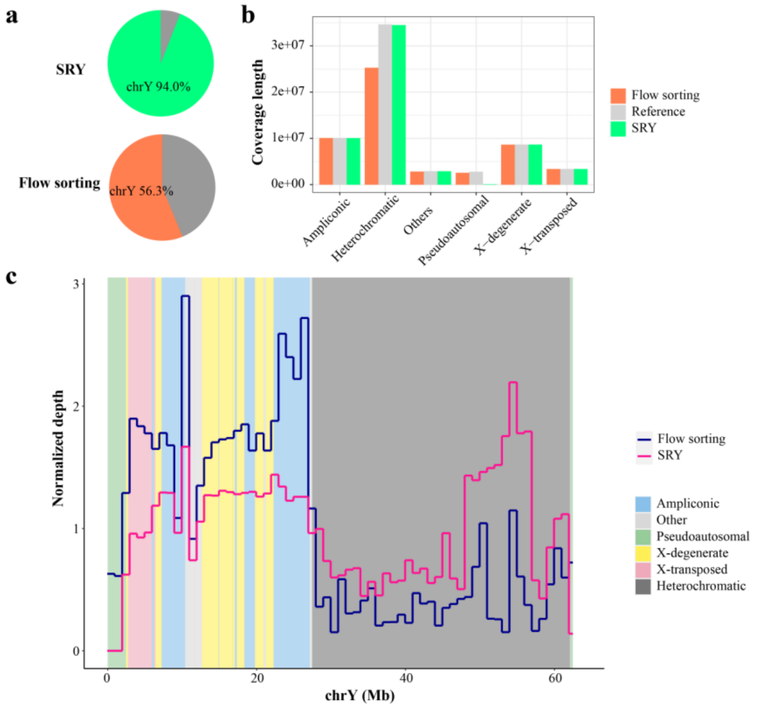

Fig. 3 Theoretical model and performance of SRY on simulated data.

Fig. 3c: What is "the normalized depth"? Why are they close to 1.5~2 in SRY, and 2 in flow sorting?

Response: Apologies for any confusion caused. The "normalized depth" in Figure 3c refers to the depth of coverage in each window, divided by the mean depth of coverage. This normalization enables comparison between different datasets. With the use of the T2T-Y reference, we can indeed observe that the normalized depth of SRY enrichment data is closer to 1 on the whole. This indicates a more uniform coverage across the entire Y chromosome.

Reviewer #2 (Remarks to the Author):

The authors propose a tool SRY for identifying male-specific reads from a set of whole-genome long-read sequenced individuals. SRY relies on identifying male-specific k-mers (MSK) inferred as specific to the Y chromosome by having half the depth of the sequencing data and being absent in a female genome. If a read contains enough MSK, it is kept. These reads can then be assembled into the Y chromosome.

The authors perform a large number of analyses using several biological and simulated datasets.

This is a commendable effort and can serve as a good source of information for other sex-chromosome-specific studies. Furthermore, the assemblies produced from only assembling SRY-selected reads outperform assembling all reads and later extract the Y contigs (Table 1). Overall, these results are compelling.

That said, some limitations in the reporting of the analysis make it difficult to compare the efficacy of their method to, e.g., flow sorting (major comment below). Also, some previous studies overlap slightly with the methodology in SRY and are not mentioned (major comment below). However, these concerns do not substantially impact the results of the study. I believe they can all be addressed by presenting more details of their data and reporting any drawbacks with SRY compared to, e.g., flow-sorting. The rest of the paper reads well (up to some minor comments) and is presented relatively succinctly and clearly.

Response: Thank you for your positive comment. We appreciate your review and would like to address each of the points you have raised in the following responses.

MAJOR

- Previous potentially related work: "Also, MSK can be used to select Y chromosome contigs from a whole genome assembly from a male individual.". Such studies are also male-specific k-mer based [1-3]. However, SRY precesses long reads, so I believe the novelty of SRY still holds.

[1] Rangavittal, et al. DiscoverY. https://doi.org/10.1186/s12864-019-5996-3

[2] Rangavittal, et al. RecoverY https://doi.org/10.1093/bioinformatics/btx771

[3] Hall AB et al. 2013 doi: 10.1186/1471-2164-14-273.

Response: The literature you provided has been added to our references.

- Tool filterX: GitHub repository should be documented better, and the tool could be described better in the methods. For example, is the input a female genome or a chromosome?

Response: The documentation of the filterX tool on the GitHub repository has been improved to provide more detailed instructions. Users can find comprehensive usage instructions by running the "filterX -h" command.

Regarding the input for the filterX tool, we utilized *k*-mer files from both male and female populations (or genomes). We labeled the *k*-mer files of all male individuals as "group1" and all female individuals as "group2". By comparing these groups and identifying *k*-mers that are present in at least 2/3 of the individuals from group1, we aim to identify specific *k*-mers associated with the male population.

These improvements will be implemented accordingly.

- Section "Comparison with other methods on real data": It is great that the authors compare SRY against flow-sorting. However, too many details about the differences between the flow-sorted (African human Y) and the regular sequencing dataset (HG01107?) processed with SRY are missing. The whole section is too vague to follow the details. For example:

- Clarify which datasets were processed with SRY. Was it only the father?

Response: SRY was used to identify Males-Specific Kmers (MSKs) from a Chinese population dataset, and these MSKs were then used for sorting third-generation data from a Chinese individual, HX1. These sorted data (HX1) were used to compare with the flow-sorted data.

We further compared the assembly results of sorted data by SRY and flow sorting. We applied

SRY to sort data from 10 individuals, and then assembled this sorted data and the flow-sorted data using wtdbg2.

To meet the requirements of the trio binning software, we collected a set of family data (Father: HG01107, Mother: HG01108, Son: HG01109). The sorted data obtained by trio binning comes from HG01109. The data processed by SRY was also from HG01109 and is included in the ten individuals mentioned.

Apologies for the confusion caused earlier. We have made revisions to the relevant paragraphs to provide clearer information.

- Both read lengths and average base pair qualities of the reads could affect the statistics reported in Figure 3. These numbers should be discussed.

Response: We thank the reviewer for bringing this important point to our attention, and we have integrated this discussion into our paper to provide a more comprehensive context for understanding our findings and their implications.

- Related to the previous point, if there is an inherent quality difference (read-length, sequence quality, or other) using flow-sorting, this should be communicated. Also, if the flow-sorting naturally leads to lower coverage (which I assume), this should be discussed.

Response: We appreciate the valuable feedback and acknowledge the importance of addressing potential quality differences between SRY and flow-sorting approaches. Flow sorting isolates chromosomes based on the difference in their DNA content or size, which might result in incomplete chromosome isolation and consequently lead to lower genomic coverage. Regarding your comment on read length and sequence quality, they should be association with the sequencing platform rather than the flow sorting method itself.

- "We [...] found that SRY has slightly higher coverage in the male-specific regions of the Y chromosome (MSY) than flow sorting". The total number of reads and total number of base pairs should be reported. Otherwise, this coverage difference does not indicate anything in regard to efficacy in extracting chrY reads. (e.g., more sequencing capacity may have gone into generating the regular dataset?).

Response: The data from the SRY sorting is presented in Supplementary Table 3 (HX1: ~3.7G，read number: 394,446). The flow sorting data has a file size of 2.3G, with a total number of reads of 305,284.

After utilizing the latest human assembly version T2T-Y, we observed a significant advantage in SRY sorting in the heterochromatic region. However, the sorting efficacy in the pseudoautosomal region (PAR) was relatively lower. This discrepancy can be attributed to the fact that the PAR region is involved in XY recombination, resulting in a lesser number of MSK.

- "Furthermore, SRY achieves better uniformity of coverage than flow sorting in the MSY" Why is this? Does flow sorting have an experimental bias regarding location? Or is the variance higher because the absolute coverage is lower? Any other statistical happening from normalizing the data?
- When I read Table 1, flow sorting has the longest aligned length and highest NA50 and NA75 (but shorter total length and slightly higher mismatch and indel rate). From these numbers, SRY does not seem favorable to flow sorting in some regards. Only the positive results compared to flow

sorting are mentioned in the section. More emphasis on the limitations/negative results should be discussed.

Response: Flow sorting isolates chromosomes based on the difference in their DNA content or size. This method may result in incomplete chromosome isolation, leading to lower genomic coverage. In contrast, SRY-based sorting specifically targets the male-specific region, which may result in a more focused and efficient extraction of reads from the Y chromosome.

Indeed, while SRY-based sorting demonstrated better performance in assembling the heterochromatic regions, it exhibited poorer assembly results in the pseudoautosomal region (PAR). These findings have been included in our manuscript.

Additionally, using the T2T-Y assembly as a reference, we found that the performance of SRY-based sorting was comparable to flow sorting. We utilized high-quality hifi and ultra-long ONT data, followed by assembly using the verkko pipeline. This improved assembly quality in the PAR region, as discussed in the manuscript's discussion section. We point out the positive impact of using high-quality data on the overall results.

- SRY relies on having access to several male and female individuals, as the accuracy of the identification of the reads only seems reasonable as the number of individuals reaches 6 (Fig. 2 A and B). This contrasts flow-sorting, which requires only one male individual of interest. The authors should make transparent that the possible advantage of flow-sorting is that only one male individual is needed in comparison to their approach.

Response: Thank you for your suggestion. We have mentioned this advantage of flow sorting in our revised manuscript as follows:

"Another consideration is that the accuracy of SRY-based sorting is influenced by the number of male and female individuals included. In contrast, flow sorting only requires one male individual of interest. "

- The tool assumes uniformly random coverage, with may not be true for real data. It would be good if the authors assess or at least comment on eventual coverage bias based on their experience working with the many biological datasets.

Response: Thank you for your valuable feedback. We would like to clarify that the SRY software does not assume uniformly random coverage as a condition. The final coverage is closely related to the distribution of specific *k*-mers in the genome. In regions with a higher abundance of specific *k*-mers, such as X-degenerate regions (Fig. 3C), SRY tends to select more reads, resulting in higher coverage. On the other hand, heterochromatic regions and PAR regions have lower abundance of specific *k*-mers (Fig. 3C), which leads to fewer reads being selected and lower coverage.

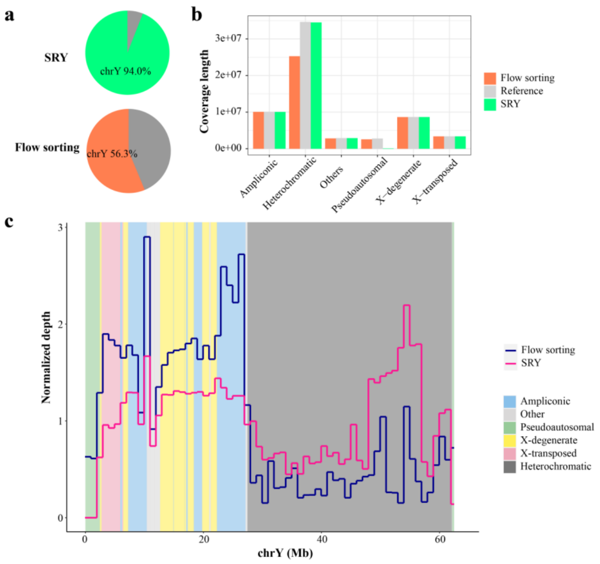

Fig. 3 Theoretical model and performance of SRY on simulated data.

- Is SRY better at classifying longer or shorter reads? An analysis to look at PPV/TPR for reads binned into different read lengths would be interesting to understand the tool's limitations.

Response: SRY classification can be influenced by various factors, such as read length and the distribution of specific k-mers. SRY uses the average density of specific k-mers in reads to make classifications. It is not necessarily the case that SRY prefers longer reads for classification. For example, let's consider a region with a length of 15 kb, where specific k-mers only appear in the first 10 kb. If the reads from this 10 kb region meet the set criteria for specific k-mer density, they will be classified by SRY. However, the average specific k-mer density of the 15 kb reads will be lower than the set criteria, resulting in them not being classified by SRY. This is demonstrated in Figure 2b (PacBio CLR vs Nanopore).

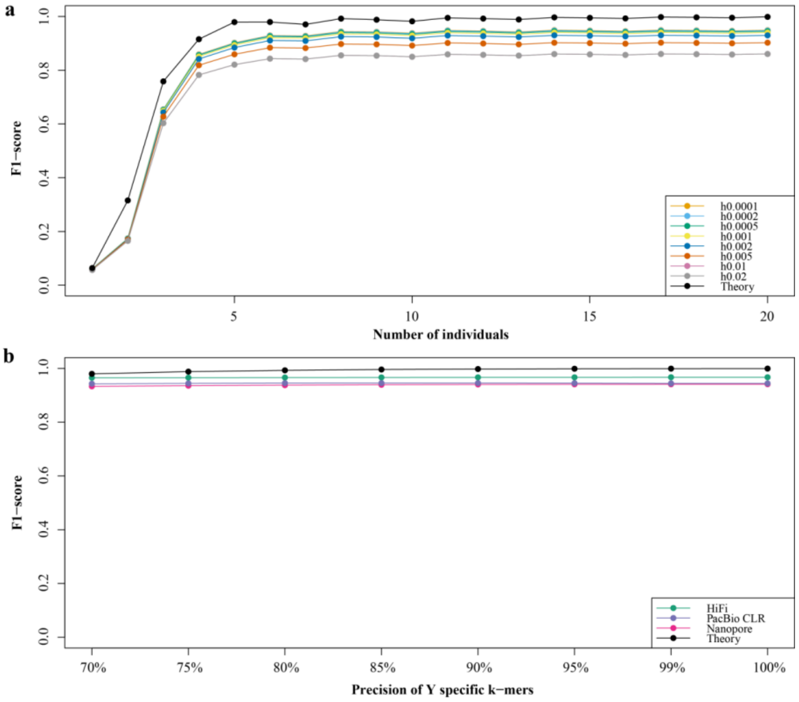

Fig. 2 Theoretical model and performance of SRY on simulated data.

- I cannot find the variable C_n^I being defined anywhere. This is quite a big mishap by the authors, given that most formulas include this variable. I don't have any reason to suspect the formulas would be wrong, though. Is C_n^i the average sequencing coverage of an individual?

Response: C represents the binomial symbol. C_n^i does not represent the average sequencing coverage of an individual. Instead, it represents the number of ways to choose i elements from a set of n elements, also known as the binomial coefficient. We have added the definition of this symbol to the article.

MINOR

* In the abstract, the authors claim SRY applies to any sex-limited chromosome without demonstration or discussion. Is the biology of W- chromosomes the same as Y, i.e. can the same assumptions be made?

Response: In our analysis, we were unable to find suitable data related to W-chromosomes to test their applicability in the same way as Y-chromosomes. Additionally, it is worth noting that differences can exist not only between W and Y chromosomes but also among Y chromosomes of different species. These differences can manifest in variations in the number and distribution of specific k-mers, which are inherent characteristics of Y or W chromosomes. Further investigation and analysis would be required to gather sufficient data and provide more conclusive insights into the biology of Y- and W-chromosomes. We have now added this point into the discussion section.

Results

* "We find that SRY is almost 100% true even when the TPR value of MSK decreases to 70% (Fig. 2c)." Overstatement. Either "near 100% accurate" and refer to (Fig. 2c and 2d) or "has near 100% specificity".

Response: We have revised the sentence to address the concern. The sentence now reads: "We find that the F1-score of SRY is larger than 90% even when the precision of MSK decreases to 70% (Fig. 2b).". F1-score is a measure that combines precision and recall to evaluate the performance of a binary classification model. It is calculated as the harmonic mean of precision and recall, ranging from 0 to 1, where a higher value indicates better performance.

* "non-specific k-mers" Is this non-MSK?

Response: Yes. We have added this abbreviation.

* "This is because longer reads are more likely to have more specific k-mers and a higher density of specific k-mers." I understand why reads have more MSK if they are longer, but why do they have higher density?

Response: Thank you for bringing this to our attention. Upon further review, we found that the conclusion in the sentence you mentioned is indeed incorrect. Longer reads are not necessarily associated with a higher density of specific k-mers. Therefore, we have already removed this statement from the manuscript.

* While the availability statement includes links for data, the authors should mention the details of the data they use in the paper. For example, avg read lengths for the ONT reads and PacBio, as well

as type of reads (PacBio HiFi or CLR) and average error rate (in regards to analysis mentioned in my major point).

Response: We apologize for the oversight in the availability statement. To address this, we have added the necessary details in Supplementary Table 3.

Sample	Long-read Category	Sorted length	Average Sorted length	Sorted Count	Total length	Average length	Count	Average error rate
**HG002**	**P**	1.5G	8.1K	190,858	234.7G	7.9K	29,809,956	14.3%
	**N**	1.8G	16.8K	104,146	184.2G	13.6K	13,586,420	10.4%
**HG003**	**P**	0.7G	7.7K	93,368	111.9G	7.5K	14,851,293	14.5%
	**N**	2.0G	11.9K	167,110	272.6G	11.4K	23,954,632	14.5%
**HG005**	**P**	1.5G	10.4K	142,173	201.3G	9.2K	21,933,756	14.3%
	**N**	1.5G	28.1K	51,927	181.3G	26.5K	6,839,693	10.7%
**HG006**	**P**	0.6G	11.4K	55,647	86.7G	9.4K	9,186,765	14.7%
	**N**	1.3G	28.0K	47,306	162.5G	26.4K	6,162,748	10.0%
**HG01109**	**N**	1.3Gb	5.3K	251,676	219.4G	8.1K	26,975,771	11.1%
**HG01243**	**N**	1.0G	7.5K	132,190	187.1G	11.5K	16,293,849	10.9%
**HG02055**	**N**	1.3G	22.9K	54,771	202.4G	20.0K	10,141,336	12.3%
**HG03098**	**N**	1.1G	10.5K	109,862	177.0G	11.1K	15,898,550	12.9%
**HG03492**	**N**	0.9G	3K	293,576	157.5G	4.4K	35,999,328	11.5%
**HX1**	**P**	2.9G	8.3K	345,760	731.7G	8.4K	87,485,724	17.0%
	**N**	0.8G	16.1K	48,686	100.3G	16.0K	6,255,000	15.0%

Supplementary Table 3. **Statistics of PacBio and Nanopore reads identified by SRY from long-read sequencing datasets.** P represents PacBio CLR reads, and N represents Nanopore reads. The average error rate is calculated based on the alignment results of the third-generation data on the human T2T genome.

* Figure 2: Does "Individual number" on the x-axis refer to individuals per sex? That is, 5 means 5 male and 5 females?

Response: Yes, a value of 5 means that there are 5 males and 5 females represented in the data.

* "subsequence of length k" -> "substring of length k" (sequence is more general in the mathematical sense and allows gaps)

Response: Corrected.

* "which is one times higher than that of flow-sorting method (Fig. 3a)" -> "twice as high" or "which is twice the fraction of ..."

Response: Corrected.

* "The total alignment length on GRCh38 Y chromosome from SRY is ~4.0Mb and ~5.6Mb longer" - Describe what is aligned. So you mean alignment length of contigs from reads produced with SRY?

Response: Yes, we have replaced all reference genomes used in our analysis with the human T2T genome and corrected the sentence to “The total contig alignment length on T2T-Y chromosome

from SRY is ~5.7Mb and ~9.6Mb longer”.

* Fig 4b: I believe the Y-axis should contain a minus, that is "-Log(SNP Num.."

Response: We have taken the suggestion from Reviewer 1 and removed the Pan-Y analysis. Instead, we have added an analysis using HiFi and ultra-long Nanopore reads for data sorting and assembly, and changed the Fig. 4.

Methods:

* k=21 is picked without discussion. This seems tailored to human, for which 21 starts to become unique. Did the authors test for suitable k during their experiments? What did they find? A brief discussion around this would be good.

Response: The trio binning method utilized k=21 for human, and the authors have discussed the impact of k-mer length in the Online method section. we have now cited the article

Koren, S. et al. De novo assembly of haplotype-resolved genomes with trio binning. *Nat. Biotechnol.* **36**, 1174-1182 (2018).

* "For third-generation long reads, the sequencing errors are higher (typically 15%)" Not true since quite a while back. PacBio typically less than 1% in HIFI and ont less than 7%.

Response: The revised sentence now reads, "For third-generation long reads, the sequencing errors are higher (PacBio CLR or Nanopore)."

* "In the model, n males and n females are sequenced with the sequencing depth d." It would be good to discuss what happens with the model if there are an unequal number of males and females and/or depths.

Response: Our objective in using data from multiple individuals is to eliminate the influence of sequencing biases and insufficient coverage as much as possible. Based on simulations with human data, we have found that having at least 6 individuals and a coverage depth of at least 5X per individual (which is typically achievable through resequencing) yields reliable results. Therefore, even if there are differences in the number of male and female individuals, as well as variations in sequencing depths, the influence on the outcomes is small (Fig. 2).

* "We also ignore the probability that k-mer-1 is identified as k-mer-2 mistakenly due to sequencing error, as such events occur with probability r/3 = 0.3%." The probability that a k-mer becomes a given other k-mer with edit distance 1 should be slightly lower; 0.99^20*(0.01/3).

Response: Corrected.

Text

* "that at lease M"

Response: Corrected.

* "specific k-mer of Y chromosome as a standard"

Response: The sentence has been corrected to “specific k-mers of the T2T-Y chromosome, which served as the standard for subsequent evaluations”.

* "We further detected novel sequences for each Y chromosome using hupan software" HUPAN software could be cited here.

Response: As discussed above, we have removed the Pan-Y analysis. Thank you for your reminder.
