# Supplementary material for: An effective strategy for assembling the sex-limited chromosome
Source: Gigascience. 2024 Apr 16;13:giae015. doi: 10.1093/gigascience/giae015 (PMC11020242; doi:10.1093/gigascience/giae015)
Supplement: giae015_GIGA-D-23-00223_Revision_1 [file giae015_giga-d-23-00223_revision_1.pdf]

GigaScience

An effective strategy for assembling the sex-limited chromosome

--Manuscript Draft--

**Manuscript Number:**	GIGA-D-23-00223R1
**Full Title:**	An effective strategy for assembling the sex-limited chromosome
**Article Type:**	Technical Note
**Funding Information:**	National Natural Science Foundation of China (91731304) — Prof Jue Ruan<br>National Key Research and Development Program of China (2019YFA0707003) — Prof Jue Ruan<br>National Natural Science Foundation of China (31860638) — Prof Qing-You Liu
**Abstract:**	Most currently available reference genomes lack the sequence map of sex-limited (such as Y and W) chromosomes, which results in incomplete assemblies that hinder further research on sex chromosomes. Recent advancements in long reads sequencing and population sequencing have provided the opportunity to assemble sex-limited chromosomes without the traditional complicated experimental efforts. Here, we introduce the first computational method, Sorting long Reads of Y or other sex-limited chromosome (SRY), which achieves improved assembly results compared to flow sorting. Specifically, SRY outperforms in the heterochromatic region and demonstrates comparable performance in other regions. Furthermore, SRY enhances the capabilities of the hybrid assembly software, resulting in improved continuity and accuracy. Our method enables ture complete genome assembly and facilitates downstream research of sex-limited chromosomes.
**Corresponding Author:**	xiaobo wang<br>Chinese Academy of Agricultural Sciences Agricultural Genomes Institute at Shenzhen<br>Shenzhen, CHINA
**Corresponding Author Secondary Information:**	
**Corresponding Author's Institution:**	Chinese Academy of Agricultural Sciences Agricultural Genomes Institute at Shenzhen
**Corresponding Author's Secondary Institution:**	
**First Author:**	xiaobo wang
**First Author Secondary Information:**	
**Order of Authors:**	xiaobo wang<br>Hong-Wei Lu<br>Qing-You Liu<br>A-Lun Li<br>Hong-Ling Zhou<br>Yong Zhang<br>Tian-Qi Zhu<br>Jue Ruan
**Order of Authors Secondary Information:**	
**Response to Reviewers:**	Dear Hans,<br><br>I am writing to submit a revised version of our manuscript entitled “An effective strategy for assembling the sex-limited chromosome”. We would like to thank the reviewers for

Powered by Editorial Manager® and ProduXion Manager® from Aries Systems Corporation

their insightful comments and constructive feedback.

In response to the reviewers' comments, we have carefully revised the manuscript to address all their concerns. Specifically, we have focused on incorporating the suggestions from the reviewers and addressing the issues raised in Reviewer 3's comment #6. We have included a detailed explanation of our approach in the revised manuscript, which we hope will clarify any confusion that may have arisen.

We believe that these changes have significantly improved the quality of our manuscript, and we hope that you will find it suitable for publication in GigaScience.

Thank you for considering our revised manuscript.

Sincerely,
Xiaobo Wang

Response to reviewers
Reviewer 1:

The authors have introduced a novel bioinformatic approach for sex chromosome assembly, addressing a persistently challenging problem in genomics. This method harnesses the full potential of whole-genome resequencing data without necessitating supplementary experimental procedures, rendering it applicable to a wide array of non-model species. Notably, the method exhibits robustness when applied to human data, surpassing established techniques such as flow-sorting and trio-binning. While the manuscript exhibits promise, several key aspects warrant refinement and elucidation to bolster its consideration for publication in GigaScience.
Response: Thank you for your insightful comments on our manuscript. We appreciate your recognition of the novelty of our bioinformatic approach for sex chromosome assembly and its potential impact on genomics research. We have provided a detailed response to each of your suggestions below.

1. Language Polishing: A degree of language refinement is advisable to enhance the overall clarity and professionalism of the manuscript.
Response: Thank you for your feedback. We have carefully revised the manuscript based on your suggestions as well as those of other reviewers. The modified sections are indicated in blue font.

2. Y Chromosome Assembly Discrepancy: The authors should acknowledge and provide an explanation for the substantial difference between the length of the latest Y chromosome assembly from T2T (~62Mb) and the assembly from SRY with Verkko (~23Mb), as detailed in Table 1.
Response: Thank you for your comment. The Flow sorting method was used to measure only Nanopore data. In order to make a fair comparison with the Flow sorting method, we selected only Nanopore or PacBio data from 10 individuals, which resulted in an assembly of only around 23Mb. We have already mentioned this limitation in the Discussion section of the manuscript.
In order to obtain a complete Y chromosome assembly and to compare with the trio mode of the verkko software, we used long-read Nanopore and HiFi data, which ultimately resulted in a ~60Mb assembly (Figure 4, SRY with Verkko) .
Therefore, the difference in assembly size is mainly due to data sources.

3. Y Chromosome Completeness: In cases where the Y chromosome assembly is incomplete, the inclusion of a figure or table delineating the proportion that SRY can recover in distinct regions of the Y chromosome would be beneficial. This could facilitate a comparative analysis of the method's efficacy across different regions.
Response: Thank you for the clarification. I apologize for any confusion caused. As mentioned in the previous response, we were able to obtain an almost complete Y chromosome assembly using long-read Nanopore and HiFi data. However, for the purpose of a fair comparison with the Flow sorting method, we obtained an incomplete assembly and displayed the assembly statistics for different regions in Table 1. To focus our discussion on the complete Y chromosome assembly, we have made the decision to present Table 1 as a supplementary material.

Powered by Editorial Manager® and ProduXion Manager® from Aries Systems Corporation

4. Figure 4 Clarity: It is imperative to label the coordinates on both the X and Y axes in Figure 4 to enhance clarity. While Figure 4 suggests that the assembly from SRY is complete compared to T2T-CHM13, the total length of the SRY assembly (approximately 23Mb) should be clearly reconciled with this observation.
Response: Thank you for your feedback. We have updated the figure to include clear coordinate labels on both axes to enhance clarity (below).

Fig. 4: Verkko assembly using Trio or SRY

5. Table 1 Organization: The organization of Table 1 should be improved to enhance readability and comprehensibility.
Response: Thank you for your suggestion. We have made improvements to enhance the readability and comprehensibility of Table 1. We have split it into two separate tables, labeled as Supplementary Table 4 and Supplementary Table 5, which are included as supplementary material.
Furthermore, we have removed some less relevant information from the tables to ensure clarity and focus on the key details.

6. MSK-Based Read Filtering: Authors should explicitly address the potential exclusion of reads from Y regions with lower than average MSK, especially in species with both young and old parts on Y chromosomes. If possible, provide recommendations or strategies for rescuing such reads.
Response: Regarding the potential exclusion of reads from Y regions with lower than average MSK (Male Specific K-mer), our intention in using average MSK as a threshold is to reduce false positives caused by sequencing errors or other factors.
In the case of humans, the differentiation time between the Y and X chromosomes is estimated to be around 300 million years ago (old Y). In our human simulated dataset (~25X coverage for the Y chromosome), we successfully captured approximately 24X (95.6%) of the Y chromosome using HiFi data and approximately 23X (91.5%) using Nanopore data.
For yellow catfish (detailed in Response to Q7), which contains a young Y chromosome with a similarity of over 99% to the X chromosome, we simulated 50X coverage of HiFi data and 50X coverage of ultra-long Nanopore data. Using SRY, we captured approximately 46X (92.2%) of the Y chromosome with HiFi data and approximately 37X (73.2%) with ultra-long Nanopore data.
Therefore, for Y chromosomes that contain both old and young parts, we recommend prioritizing HiFi data as the loss of data will have minimal impact on assembly. If feasible, we also suggest including ultra-long Nanopore data to increase the completeness of the assembly results.

7. Simulation for species with young sex chromosomes: It is essential to conduct additional simulations for testing the efficiency of isolating Y reads for species with young sex chromosomes. This analysis should consider the variation between X and Y chromosomes, aiding researchers in evaluating the method's suitability for their specific study organisms.
Response: We have conducted simulations to test the efficiency of isolating Y reads for species with young sex chromosomes, using the yellow catfish as a model organism [1]. This species contains a young Y chromosome with a size of approximately 43.2 Mb, which shares greater than 99% similarity with the X chromosome. In our simulations, we generated 100X HiFi and Nanopore data (50X for the Y chromosome) and obtained 46X of HiFi and 37X of ultra-long Nanopore data after sorting for the Y chromosome. Using the verkko software, we assembled the Y chromosome into two contigs with a total size of 42.7 Mb. These results demonstrate that our method is effective for assembling the Y chromosome of species with young sex chromosomes.We have incorporated these results into our manuscript.

[1] Gong G, Xiong Y, Xiao S, et al. Origin and chromatin remodeling of young X/Y sex chromosomes in catfish with sexual plasticity. National Science Review, 2023, 10(2): nwac239.

Addressing these points will further strengthen the manuscript's scientific rigor and its suitability for publication in GigaScience.
Response: Thank you for your valuable feedback.

Powered by Editorial Manager® and ProduXion Manager® from Aries Systems Corporation

Reviewer 2:
The SRY method, developed and evaluated for sorting long reads of sex-limited chromosomes, has shown promise in effectively identifying and sorting sequences based on sex-specific markers, particularly the Y chromosome. These sorted long reads are then utilized for genome assembly. Additionally, the SRY method can be used to select Y chromosome contigs from a male individual's whole genome assembly.Overall, the success of SRY in sorting and assembling long reads of sex-limited chromosomes highlights its potential as an alternative to experimental methods for studying sex-specific genomic regions.
Response: Thank you for your positive comments.

Here are some comments for further improvement of manuscript:
1) The authors may want to consider to presenting a table for standard evaluation metrics (k-mer or alignment-based). See Garg 2021 (https://genomebiology.biomedcentral.com/articles/10.1186/s13059-021-02328-9).
Response: Thank you for your suggestion. Table 1 presents the standard evaluation metrics for genome assembly using quast, an alignment-based software that is widely used for assessing genome assembly results. However, we have made some changes based on the feedback from other reviewers. The updated Table 1 has been moved to supplementary materials and is now labeled as Supplementary Table 4 and 5. We believe that these optimizations enhance the presentation of our results.

2) Adding a few important genes that are medically relevant and assembled properly may further add value to the work.
Response: In our study, we found that the assembly of gene sequences is generally not complex and can be relatively straightforward. Using the Verkko assembly method, both the direct assembly and the assembly after SRY sorting yielded comparatively complete genes on the Y chromosome. However, we observed that the assembly after sorting improved sequence contiguity, which is beneficial for analyzing regulatory sequences near the genes. We acknowledge that further investigation is needed to explore this aspect in future research. We appreciate your feedback.

Reviewer 3:
1.In the introduction, add recent marker based graph phasing algorithms in long-reads, such as hifiasm trio and verkko trio mode after the T2T-Y. They are different from trio-binning, which tries to phase the reads upfront. Graph based phasing is using markers to determine haplotype specific paths to traverse.
Response: Thank you for your feedback. In the introduction, we include recent marker-based graph phasing algorithms in long-reads, such as hifiasm trio and verkko trio mode, after discussing T2T-Y.

a. T2T-Y chromosome should be referencing Rhie et al., Nature 2023. Verkko is a successor of the manual efforts taken in T2T-Y, which should be also noted in the introduction.
Response: We have made the necessary updates to reference Rhie et al., Nature 2023 as the source for the T2T-Y chromosome. Additionally, we have included a note in the introduction to acknowledge that Verkko is a successor of the manual efforts undertaken in T2T-Y.

b. Reference for sexPhase program is still missing. Also, some rephrasing of the sentence is needed, as the way it is currently written is easily misleading to be understood as sexPhase was part of the methods used in the assembly of the T2T-Y.
Response: Thank you for pointing that out. The reference for the sexPhase program is a article titled "Genomes of the Banyan Tree and Pollinator Wasp Provide Insights into Fig-Wasp Coevolution," published in Cell, 2020. This software was specifically used for the assembly of the fig tree sex chromosomes mentioned in the article and does not have general applicability. Therefore, it is not suitable to be included as the latest development after T2T-Y. We have removed this content and added a description of marker-based graph phasing algorithms instead.

2. There are other approaches for phasing genomes taken in plants, for example the poly ploid potato phasing using many siblings of the child by Mari et al. bioRxiv 2022.
Response: Thank you for your suggestion. We have incorporated the information into the Introduction section of our paper.

Powered by Editorial Manager® and ProduXion Manager® from Aries Systems Corporation

3. "But only one male and one female could suffer from sampling error" - this part is unclear. Please clarify.
Response: We apologize for the unclear wording in the previous version. We have since made revisions to the manuscript and have updated this section to read: "By incorporating population data into our analysis, we anticipate a reduction in the impact of sequencing coverage and allelic genotype variations when identifying Y chromosome-specific markers in a comparative analysis involving only two individuals of different genders." We hope that this revised statement is clearer and more accurately reflects our intended meaning.

4. Reference for the mason_simulator, badread software is missing.
Response: Corrected.

5. Provide the accession (HG02982) for the "African human Y" in the main text.
Response: Accession (HG02982) for "African human Y" has been added now.

6. I appreciate that the authors compared assemblies to T2T-Y as I requested before. However, fundamentally, mapping to T2T-Y and comparing length of each sequence classes is comparing apples to oranges, particularly in the heterochromatic region and ampliconic region of the Y. It is known to have variable copy numbers and size differences between two individuals. Frequent inversions have been reported in the ampliconic regions across different Y haplogroup. The number, size, and distribution of the repeat arrays composing the heterochromatic region has been shown to vary among different Y haplogroups in Hallast et al., Nature 2023. This can be also seen in Fig. 3c; the overall depth of the flow sorting in the heterochromatic region is below 1 - indicating the Yqh is shorter than T2T-Y, as it is in Fig. 3b. To make the benchmark legit, the authors should compare SRY and the flow sorting method using samples from the same individual. HG02982 and HX1 are presumably having very different sequence compositions given the diverged population history (African vs. Asian). Comparing total length of the assembled region against a 3rd different Y haplogroup (HG002Y) makes things more complicated, especially on regions that are known to vary a lot. If the authors think flow sorting based method needs to be compared, it should be benchmarked on the same individual to make an apple-to-apple comparison. I do agree results from read sorting (i.e. portion of reads sequenced from non-Y chromosomes in SRY vs. flow-sorting) is an important finding. However, I'd still argue comparing assemblies from the two different Y haplogroups is a stretch. The authors could have performed the same assembly length comparison on the T2T-Y using results from their SRY sorted reads with Verkko of HG002 vs. Verkko assembly using trio-binned markers.
Response: We completely agree with your points. As you mentioned, the comparison results from read sorting are indeed important findings. We acknowledge that using a different Y haplogroup for assembly length comparison may not be ideal. In our study, we did not have access to publicly available HG02982 whole genome long-read data, so we could only compare the SRY sorting results from another sample (HX1) to the flow sorting results. As a methodological study, we currently do not have the capability to collect HG02982 samples and perform third-generation sequencing. Even if we were able to obtain HG02982 samples, there could be inconsistencies in sequencing data length and accuracy due to updates in sequencing platforms, which would compromise the fairness of the comparison of the sorting results. However, we have taken your suggestion and compared and analyzed the sorting results of HG002 in the subsequent results section. In addition to your valuable feedback, we have also taken into consideration other suggestions from the reviewers. As a result, we have decided to move the Table 1 to the supplementary materials (Supplementary Table 4-6) to shift the focus on the assembly results of the complete Y chromosome. Thank you for your constructive comments, and we hope that the revised manuscript will meet your expectations.

7. In the section where assemblies are compared, the authors point to Table 1, which contains results from HG01109. HG01109 has never been mentioned before. I thought the authors were comparing assemblies from SRY sorted reads of HX1? I am not sure why the authors suddenly added a 3rd PUR genome with no context. Was this a mistake? Add results from HX1 to Table 1.
Response: We apologize for any confusion caused. The population data we collected

Powered by Editorial Manager® and ProduXion Manager® from Aries Systems Corporation

for our study is representative of the Chinese population, and the long-read sorting data mentioned, HX1, is an individual of Chinese. In order to compare the assembly results with the trio binning method, we included the sample HG01109, which is part of a trio dataset consisting of the parental HG01107 and HG01108, along with their offspring HG01109. To clarify this in the manuscript, we have added the following explanation: "We collected a trio dataset consisting of the parental genomes HG01107 and HG01108, as well as the offspring genome HG01109, in order to perform trio binning and compare the resulting assemblies of HG01109." We have now included the results from HX1 in Supplementary Table 6 for a more clear presentation of the comparisons.

8. Please add divider lines in Table 1 between All / Ampliconic / X-degenerate / X-transposed / PAR / Het / Others. It is hard to see which rows belong to which category.
Response: Corrected in Supplementary Table 4-6.

9. The last result section where authors compare results from Verkko, it is unclear how the verkko assembly was run. The authors say "default option", and later "in trio mode" in the methods. Did the authors collect parental reads from HG002 (HG003 and HG004)? How was "trio mode" performed? Did the authors used trio binning to sort the reads, then run Verkko? Or used the homopolymer compressed parental kmers and used that in the Rukki step of Verkko (and this should be benchmarked)? Was the HG002 trio assembly taken from Rautiainen et al. paper? Please clarify and add the missing parts to the main text and methods.
Response: We apologize for the lack of clarity in our manuscript regarding the Verkko assembly. To clarify, we ran the Verkko assembly with the following parameters: "-d Asm --hifi hifi.sorted.fq.gz --nano ul-ont.sorted.fq.gz --threads 128". The HG002 trio assembly used in our study was obtained from the Rautiainen et al. paper. We have updated the methods to include this information.

10. Related to the above section, it is hard to see in Fig. 4a the "two approximately 1 Mb contigs aligning to the same region of the Y chromosome". An enlarged inset of the dotplot may be helpful. Also, add legends and scale to the X and Y axis of the dotplots.
Response: We have updated Figure 4a to include an enlarged inset of the approximately 1 Mb region, and added legends and scale to both X and Y axes of the dotplot. Please see the Fig.4 below:

11. Note there is a mis-assembly reported on T2T-Y palindrome P5 (https://github.com/marbl/CHM13-issues/blob/main/v2.0_issues.bed), which the entire P5 should be inverted. I don't see this in the dotplots of Fig. 4.
Response: Thank you for bringing this to our attention. Upon reviewing the alignment of T2T-Y palindrome P5 (18483410-19235627), we have identified an approximately 3.5kb inversion (18857803-18861259) in the assembly generated by the verkko sry mode. Due to its relatively short alignment length, this inversion may not be clearly visible in the dotplots of Figure 4. Additionally, we have performed assembly using hifiasm in the P5 region and did not find any inversions. This indicates that the current approach (SRY) of read sorting may not effectively resolve the situation with P5. We have made a note of this in Figure 4 to provide clarification.

12. In the discussion, the authors are mentioning results from the 10 trios that have been removed from the previous results. Please add the 10 trio results to the main text if it was a mistake, or remove the irrelevant results from the Discussions and Supp. Tables.
Response: We apologize for any confusion caused. In our study, we collected data from 10 individuals, out of which only HG01109 had trio data. The previous removal referred to the pan-genome analysis of these 10 individuals. The assembly results of the 10 individuals based on SRY sorting were mainly used for comparison with the flow sorting method (Supplementary Table 4 and 6). We have deleted the sentence “we limited our dataset collection to Nanopore or PacBio CLR data for the 10 individuals” in the Discussion section.

13. The authors discuss the suboptimal performance of SRY in the PAR is contributed by the restricted data types. I thought it was contributed by the lower density of the markers? The PAR parental marker density was very similar to that of autosomes, with

Powered by Editorial Manager® and ProduXion Manager® from Aries Systems Corporation

	stretches of runs of homozygosity, presumably to maintain enough homology for recombination. What was the marker density in the PAR? Was it below their 7 kmer / 1kb?<br>Response: Thank you for your question. You are correct that the suboptimal performance of SRY in the PAR is due to the lower density of markers. We apologize for any confusion caused by our statement. We have re-examined the data and found that there are no MSK in the PAR region. Considering the coherence of the context, we have removed the sentence in question.<br><br>14. The authors mentioned there are no ZW genomes available to test SRY. There is a Zebra finch trio (ZW, female, bTaeGut2) and a male sample (ZZ, male, bTaeGut1) available with HiFi of the child (bTaeGut2) and Illumina of all the genomes from the Vertebrate Genomes Project (Rhie et al., Nature, 2021). Perhaps the authors could apply SRY on this individual, and compare the W chromosome results to what has been released on https://www.genomeark.org/vgp-all/Taeniopygia_guttata.html.<br>Response: Thank you for providing the information about the availability of ZW genomes for testing SRY. We firstly performed sex determination on seven individuals using the chrW sequence from the reference genome. Our analysis revealed that four individuals were female while three were male. Utilizing SRY, we obtained 19,128,829 specific kmers and sorted approximately 275.8 Mb of HiFi sequences. We assembled the sorted HiFi sequences using hifiasm and verkko softwares. Comparing the two assemblers, we found that hifiasm showed slightly better results (hifiasm total length: 19.7 Mb, N50: 516.3 Kb; verkko total length: 18.1 Mb, N50: 478.3 Kb). The assembly size from hifiasm closely approximated the size of the reference genome (~20.0 Mb when exclude N) and shows good collinearity (figure below). However, our assembly results did not cover the PAR region, which is a limitation of our method.
**Additional Information:**	
**Question**	**Response**
Are you submitting this manuscript to a special series or article collection?	No
**Experimental design and statistics**<br><br>Full details of the experimental design and statistical methods used should be given in the Methods section, as detailed in our Minimum Standards Reporting Checklist. Information essential to interpreting the data presented should be made available in the figure legends.<br><br>Have you included all the information requested in your manuscript?	Yes
**Resources**<br><br>A description of all resources used, including antibodies, cell lines, animals and software tools, with enough information to allow them to be uniquely identified, should be included in the Methods section. Authors are strongly encouraged to cite Research Resource	Yes

Powered by Editorial Manager® and ProduXion Manager® from Aries Systems Corporation

Identifiers (RRIDs) for antibodies, model organisms and tools, where possible.<br><br>Have you included the information requested as detailed in our Minimum Standards Reporting Checklist?	
**Availability of data and materials**<br><br>All datasets and code on which the conclusions of the paper rely must be either included in your submission or deposited in publicly available repositories (where available and ethically appropriate), referencing such data using a unique identifier in the references and in the “Availability of Data and Materials” section of your manuscript.<br><br>Have you have met the above requirement as detailed in our Minimum Standards Reporting Checklist?	Yes

Manuscript Click here to access/download;Manuscript;Manuscript-r.docx

An effective strategy for assembling the sex-limited chromosome

Xiao-Bo Wang[1#], Hong-Wei Lu[1#], Qing-You Liu[2#], A-Lun Li[1] , Hong-Ling Zhou[1], Yong Zhang[5], Tian-Qi Zhu[3,4*], Jue Ruan[1*]

1. Shenzhen Branch, Guangdong Laboratory for Lingnan Modern Agriculture, Genome Analysis Laboratory of the Ministry of Agriculture and Rural Affairs, Agricultural Genomics Institute at Shenzhen, Chinese Academy of Agricultural Sciences, Shenzhen, Guangdong 518120, China
2. Guangdong Provincial Key Laboratory of Animal Molecular Design and Precise Breeding, School of Life Science and Engineering, Foshan University, Foshan 528225, China
3. National Center for Mathematics and Interdisciplinary Sciences, Academy of Mathematics and Systems Science, Chinese Academy of Sciences, Beijing 100190, China
4. Key Laboratory of Random Complex Structures and Data Science, Academy of Mathematics and Systems Science, Chinese Academy of Sciences, Beijing 100190, China
5. Key Laboratory of Zoological Systematics and Evolution & State Key Laboratory of Integrated Management of Pest Insects and Rodents, Institute of Zoology, Chinese Academy of Sciences, Beijing 100101, China

[#]These authors contributed equally to this work.

[*]Corresponding author: E-mail: ruanjue@caas.cn; zhutq@amss.ac.cn

Abstract

Most currently available reference genomes lack the sequence map of sex-limited (such as Y and W) chromosomes, which results in incomplete assemblies that hinder further research on sex chromosomes. Recent advancements in long reads sequencing and population sequencing have provided the opportunity to assemble sex-limited chromosomes without the traditional complicated experimental efforts. Here, we introduce the first computational method, Sorting long Reads of Y or other sex-limited chromosome (SRY), which achieves improved assembly results compared to flow sorting. Specifically, SRY outperforms in the heterochromatic region and demonstrates comparable performance in other regions. Furthermore, ~~we observed that~~ SRY enhances the capabilities of the hybrid assembly software, resulting in improved continuity and accuracy. Our method enables true complete genome assembly and facilitates downstream research of sex-limited chromosomes.

Introduction

Traditionally, genomes of homogametic individuals (XX females or ZZ males) have been preferred for genome sequencing projects, because the haploid nature of both sex chromosomes (X and Y, or Z and W) in heterogametic species ~~provide~~ reduces sequencing depth ~~that~~ which can lead to decreased assembly contiguity and length[1]. ~~While~~ Although XY or ZW chromosomes have significantly diverged from their ancestral autosomes[2, 3], they still exhibit homology, which can ~~still~~ pose challenges for genome assembly. Homologous regions, such as the pseudoautosomal regions (PAR), can ~~lead to~~ result in fragmented contigs similar to large repeats. Plenty of repetitive sequences in sex-limited (Y or W) chromosome further increase the assembly difficulties.

~~So far~~ Currently, there are ~~principally~~ two main experimental approaches aimed at solving the problem. The first ~~one~~ approach, known as the BAC-based method, has been applied in deciphering the Y chromosomes of several mammals including human[3], chimpanzee[4], rhesus macaque[5], and mouse[6]. However, it is time-consuming, labor-intensive and expensive. The ~~other one~~ second approach is chromosome flow-sorting, which ~~is based~~ relies on chromosome size and GC content (**Fig. 1a**)~~, with~~ and offers high automation and ~~high~~ throughput[1]. However, it requires cells to be in metaphase, where chromosomes are in a condensed state that ~~are~~ can be easily physically separated[7]. ~~Moreover~~ Additionally, it may mistakenly sort other chromosomes or debris ~~having~~ with similar sizes or GC contents ~~with~~ to the sex-specific chromosome, ~~and bring in~~ which can introduce bias during the amplification stage[1, 8].

Takashi *et al.* used the F_1 population data of the persimmon to identify male-specific markers[9], and utilized these markers to partition and assemble short reads. However, they did not provide software for the algorithm~~, nor do they~~ or consider the effect of population heterogeneity on the identification of male-specific markers. YGS[10] compares the male assembly results with length k subsequences (*k*-mers) of female short reads to obtain Y contigs. The Sex-detector[11] uses pedigree data to identify sex-specific genes in RNA-seq assemblies. However, all three methods lack the ability to sort long reads to reduce the assembling difficulty.

Thanks to ~~both~~ the longer read lengths and higher sequencing accuracy, long reads have a higher potential to be identified to ~~its~~ their original chromosome by pure computing methods. Recently, trio binning was developed to sort long reads *in silico*[12] using specific markers (**Fig. 1b**). It compares *k*-mers of short reads from parental genomes and identifies *k*-mers that are unique to each parent. ~~Trio binning further uses~~ tThese *k*-mers are then used to separate long reads of the

offspring and conducts ~~*de novo*~~ haplotype assemblies, separately. Theoretically, Y- (or W-) specific markers[13] can be selected and used for sorting long reads from sex-limited chromosome. Compared to whole genome shotgun (WGS) assembly, trio binning assembly covers more genomic regions of the Y chromosome with ~~a~~ better contiguity (~~**Table 1**~~ **Supplementary Table 4**). It indicates that computational method is promising, though trio binning cannot efficiently address the problem of assembling sex-limited chromosome based on its scheme to select specific markers. The Telomere-to-Telomere (T2T) consortium has used ~~a variety of~~ various third-generation sequencing technologies to complete the assembly of the Y chromosome[13], but the ~~assembly~~ process requires ~~plenty of~~ substantial manual adjustments. It is worth noting that recent marker-based graph phasing algorithms in long-reads, such as hifiasm[14] trio and verkko[15] trio mode, have emerged as alternative approaches to enhance the accuracy and efficiency of phasing. Specially, verkko is a successor of the manual efforts taken in T2T-Y. Additionally, there are alternative approaches for phasing genomes in plants. For instance, Mari et al.[16] proposed a polyploid potato phasing method that utilizes many siblings of the child for genotyping, and achieved significant results.

To further improve the quality of sex-specific chromosome assembly and reduce the need for manual curation, we try to find new solutions from population datasets. In whole genome sequencing, the sequencing depth between sex chromosomes and autosomes is different. For example, ~~Take~~ in a XY male ~~as an example~~, the sequencing depth of the X or Y chromosome is half that of autosomes. ~~Thus~~ There, X/Y-specific markers can be separated by different sequencing depth. Moreover, the X-specific markers also exist in females, so they can be removed from X/Y-specific markers to obtain Y-specific markers. By incorporating population data into our analysis, we anticipate a reduction in the impact of sequencing coverage and allelic genotype variations when identifying Y chromosome-specific markers in a comparative analysis involving only two individuals of different genders.

Results

Overview of SRY

To reach the goal of sorting long reads of sex-limited chromosome, we developed an *in silicon* sorting method called SRY (**Fig. 1c**). The ~~basic principle~~ process of SRY ~~is to obtain~~ involves identifying sex-specific markers by comparing male and female populations, and subsequently sorting long sequences according to the specific markers. ~~To be specific,~~ SRY firstly selects *k*-mers with half of the sequencing depth in male populations. ~~Then~~ Subsequently, SRY filters out X-linked *k*-mers and *k*-mers originating from heterozygous sites ~~present~~ found in female populations, thereby enabling the identification of male-specific *k*-mers (MSK). Owing to the impact of population structure and sequencing errors, the operation of SRY is in fact a sampling process, which unavoidably involves *k*-mers from X chromosome and autosomes. ~~So~~ Therefore, SRY calculates the MSK density of long reads and excludes those with lower marker density. These separated long reads are ~~further~~ subsequently delivered to assemblers to perform genome assembly. ~~Also~~ Moreover, MSK can be used to select Y chromosome contigs from a whole genome assembly from a male individual[17-19].

Evaluating SRY with theoretical models and simulated data

There are two ~~main sources~~ primary factors contributing to false positives ~~for~~ in the identification

of specific *k*-mers by SRY: ~~one is~~ coverage and ~~the other is~~ population heterogeneity. Accordingly, we constructed theoretical models of the false positive and true positive to assess MSK identified by SRY (see the Methods section for details). In addition, we used the mason_simulator[20] software to simulate the data under different heterogeneity and different number of individuals (5X for each individual) for the evaluation of SRY. Consistent with the theoretical results, the results based on simulated data show that an increase in population heterogeneity ~~lessens~~ decreases the F1-score of SRY, while an increase in the number of individuals augments the F1-score of SRY (**Fig. 2a**). Notably, when the number of individuals in both male and female populations is less than 7, the increase in the number of individuals has a significant effect on improving the F1-score of SRY (**Fig. 2a**). However, ~~when~~ once the number ~~is more than~~ exceeds 7, the increase in the number of individuals has little effect (**Fig. 2a**).

Furthermore, we provide a theoretical model for the process of sorting long reads of Y chromosome (see Methods section for details). The central issue addressed by this model is determining the probability that at least M markers are retained in the corresponding error-prone long reads within a genomic region consisting of N specific markers. In addition to theoretical values, we used badread[21] software to simulate the human T2T genome with 50X each of HiFi, Nanopore and PacBio CLR reads, and set a ~~series~~ range of MSK precision level to assess the performance of SRY on sorting long reads. Remarkably, even when the precision of MSK decreases to 70%, we found that the F1-score of SRY remains above 90% (**Fig. 2b**). This ~~is because~~ can be attributed to the fact that the genome size of autosomes and X chromosome is ~~about~~ approximately 3Gb, resulting in a low density of non-specific k-mers (non-MSK) derived from these chromosomes (1 k-mer/kb). The filter ~~condition~~ criterion of SRY is ~7 *k*-mers per kilobase, ~~so~~ making it ~~is~~ easy to ~~filter out~~ exclude these non-Y chromosome sequences.

Comparison with the experimental method on real data

We collected datasets including short and long reads of a Chinese individual HX1[22,23], and re-sequencing short reads of a Han Chinese population[24] to identify MSK (**Supplementary Table 1 and Table 2**). SRY obtained about 10 million MSK as well as sorted 3.7G (~46X) PacBio CLR and (~13X) ONT long reads of Y chromosome (**Supplementary Table 3**). We further collected Nanopore long reads (~2.3G, number of reads is 305,284) of an African human Y (HG02982) separated by flow sorting[8] and used minimap2[25] to align the sorted long reads from the two methods to the human T2T genome, separately. The results show that 94.0% of the sorted reads from SRY are mapped on T2T-Y chromosome, which is significantly higher than that of flow sorting (**Fig. 3a**). The human Y chromosome consists of several distinct regions (ampliconic, X-degenerate, X-transposed, pseudoautosomal, heterochromatic, others)[3]. We compared the performance of the two methods in these regions and found that SRY demonstrates comparable performance to flow sorting in the ampliconic, X-degenerate, X-transposed and others (**Fig. 3b**). However, SRY outperforms in terms of coverage and depth specifically in the heterochromatic region. (**Fig. 3b, c and ~~Table 1~~ Supplementary Table 4**). Notably, the event of X-Y recombination is frequent on pseudoautosomal region (PAR, PAR1:1-2.8Mb, PAR2:56.9-57.2Mb). SRY aims to obtain Y-specific markers, hence the low coverage and shorter assembled result on PAR of SRY is expected (**Fig. 3b and ~~Table 1~~ Supplementary Table 4**).

We further compared the resulting assemblies between the experimental and computational methods. We collected a trio dataset consisting of the parental genomes HG01107 and HG01108,

as well as the offspring genome HG01109, in order to perform trio binning and compare the resulting assemblies of HG01109. SRY can achieve sort reads first and then assemble, or directly sort the assembled contigs based on MSK. The fast assembler wtdbg2[26] was used to assemble those sorted reads and flow sorting reads, and perform genome assemblies for trio binning and WGS (**Supplementary Table 3**). The total contig alignment length on T2T-Y chromosome from SRY is ~5.7Mb and ~9.6Mb longer than those from the sorted contigs of trio binning and WGS respectively. Moreover, the alignment lengths and the contiguity (NA50) on each discrete region of Y chromosome from SRY are all longer than the other two methods (~~**Table 1**~~ **Supplementary Table 5**), indicating that it is better to sort the reads first and then assemble them. Additionally, the SRY assembly exhibits lower contamination from other chromosomes compared to flow sorting (~~**Table 1**~~ **Supplementary Table 4**). Similarly to the result obtained from read sorting, the assembly result from SRY performed better in heterochromatic regions compared to flow sorting. However, in the PAR, the assembly result from SRY was inferior to those from flow sorting.

Towards complete genome assembly of Y chromosome

Mikko et al[15] developed an assembly software called Verkko, designed for HiFi and ultra-long Nanopore data, in order to achieve better automation of T2T-level chromosome assembly. Verkko demonstrated good result in the assembly of HG002. We sorted the Y chromosome data from HG002 and used Verkko for assembly. The results showed that, compared to Verkko with trio[15], the assembly of the sorted data (Verkko with SRY) reduced the number of contigs from 23 to 9 and corrected one assembly error (**Fig. 4**). Additionally, due to the high similarity of the X and Y chromosome PAR regions, Verkko trio's assembly result did not phasing this region well, resulting in two approximately 1Mb contigs aligning to the same region of the Y chromosome (**Fig. 4a**). Verkko SRY not only assembled this region completely, but also with higher accuracy (**Fig. 4b**). This indicates that SRY can further improve the performance of the assembly software with new sequencing technology.

We further selected the genome of the yellow catfish[27], which contains young Y chromosomes, for simulated evaluation. The similarity between its Y chromosome and X chromosome exceeds 99%. To facilitate evaluation, we removed the gaps in the genome. We simulated 100X HiFi and ultra-long Nanopore data (50X for the Y chromosome) respectively. As a result, we sorted ~45X HiFi data and ~37X ultra-long Nanopore data of the Y chromosome. The assembly size of the results using verkko software was 42.7Mb (98.8% of the Y chromosome), consisting of only 2 contigs. According to the quast results, both contigs aligned almost perfectly to the reference Y chromosome. This indicates that our software can achieve relatively good results in assembling young Y chromosomes as well.

Discussion

The deciphering of sex chromosomes is crucial for studying reproductive biology, sex determination, and other key molecular processes that contribute to the evolutionary trajectory of species. To enable more complete assembly of sex chromosome sequences, we have developed the SRY method, a software tool that efficiently sorts third-generation long reads of sex-limited chromosomes based on male-specific k-mer (MSK) markers.

The performance of SRY can be influenced by the number of male and female individuals, whereas flow sorting only requires one male individual of interest and is not affected. However,

SRY outperformed flow sorting in terms of ~~mapping reads to the Y chromosome~~ read sorting accuracy and demonstrated comparable or better performance in most regions, except for the pseudoautosomal region (PAR) where SRY had lower coverage and assembly results. The effectiveness of SRY is also influenced by the lengths of the reads and the quality of the base pairs. ~~Nevertheless, it is worth mentioning that~~ Notably, the incorporation of HiFi and ultra-long Nanopore data significantly improved the assembly quality of SRY in the PAR region. The capability of SRY to effectively sort long reads of sex-limited chromosomes (regardless of their age), highlight its potential as a valuable alternative to experimental methods for studying sex-specific genomic regions.

With the further reduction in sequencing costs, there will be a greater availability of population-level second and third-generation sequencing data. After identifying MSK using second-generation data, SRY can be utilized for sorting and assembling third-generation long reads from all individuals within the population. The application and comparison of sex chromosomes within the population will contribute to our understanding of complex biological processes and genetic variations.

Methods

SRY process

Firstly, SRY used kmer_count program to acquire *k*-mer (*k*=21)[12] sets from short reads of targeted male species and populations. Next, the program filterx (https://github.com/ruanjue/filterx) is used to identify specific *k*-mers associated with the male population. We labeled the *k*-mer files of all male individuals as "group1" and all female individuals as "group2". By comparing these groups and identifying *k*-mers as specific *k*-mers that are present in at least 2/3 of the individuals from group1. Then, SRY selects long reads of targeted species that have male specific *k*-mers (MSK). Finally, SRY filters those long reads with lower MSK densities than average value of whole Y chromosome.

False positive for MSK

False positive is introduced if a subsequence of length k (k-mer) originated from autosomes or X chromosome is incorrectly identified as a MSK, with two possible sources: genomic coverage and population heterogeneity.

In the model, n males and n females are sequenced with the sequencing depth d. The length of a read is l and the sequencing error per site is r. C_n^i represents the number of ways to choose i elements from a set of n elements, also known as the binomial coefficient. In an individual, only the frequency of appearance of a k-mer that is more than once can we consider it to be present, and this event occurs with probability p_o. A k-mer is identified as an MSK if it is present in at least m males but not in any of the females. Particularly, we use $m = 2/3n$ as the critical value by simulation study.

Let X be the frequency of appearance of a k-mer is present in an individual, then it follows a Possion distribution with rate $\lambda = d(1-r)^k(l-k+1)/l$. It is easier to calculate $q_o = 1 - p_o$, which is the probability that a k-mer is absent, and thus

$q_o = P(X=0) + P(X=1) = e^{-\lambda} + \lambda e^{-\lambda} = (1+\lambda)e^{-\lambda}$

We then can calculate the false positive rate caused by genomic coverage (f_1) by

$$f_1 = q_o^n \sum_{i=\lceil \frac{2}{3}n \rceil}^{n} C_n^i p_o^i q_o^{n-i} \tag{0.1}$$

If we set r = 0.01, l = 150, d = 5, k = 21 and n = 5, then f_1 is roughly 4.4×10^{-5}. If the sample size n increases to 10, then f_1 decreases to 1.9×10^{-9}, indicating that the false positive introduced by genomic coverage can be ignored if the sample size n is not too small.

For simplicity, we only consider heterogeneity in autosomes, and ignore heterogeneity in X chromosome. Assume one heterozygous site leads to two kinds of k-mers (k-mer-1 and k-mer-2), and the heterozygous proportions for two k-mers are p_{h1} and p_{h2} (with $p_{h1} + p_{h2} = 1$). If k-mer-j (j=1,2) from autosomes is observed in many males while it is not observed in any females, then k-mer-j is mistakenly identified as an MSK. As the discussion for the case of genomic coverage, the frequency of appearance of k-mer-j, X_j, follows Poisson distributions with parameters $\lambda_j = p_{hj}\,\lambda = p_{hj}\, d\, (1-r)^k(l-k+1)/l$. As before, if a k-mer appears less than twice in an individual, we consider the k-mer is absent, and the probability of this event q_{oj} can be calculate as follows
$q_{oj} = P(X_j = 0) + P(X_j = 1) = e^{-\lambda j} + \lambda_j\, e^{-\lambda j} = (1+\lambda_j)\, e^{-\lambda j}$.
Given the heteropoietic rate h = 0.001, the probability that a k-mer with length 21 contains more than one heterozygous site is only 2.1×10^{-4}, which can be neglected. We also ignore the probability that k-mer-1 is identified as k-mer-2 mistakenly with edit distance 1 due to sequencing error, as such events occur with probability 0.99^20*r/3 = 0.3%. Then the false positive rate due to population heterogeneity f_2 can be calculated as follows:

$$f_2 = q_{o1}^n \sum_{\lceil i=\frac{2}{3}n \rceil}^{n} C_n^i p_{o1}^i q_{o1}^{n-i} + q_{o2}^n \sum_{\lceil i=\frac{2}{3}n \rceil}^{n} C_n^i p_{o2}^i q_{o2}^{n-i}. \tag{0.2}$$

The total false positive rate f is a weighted average of the false positive rate from the two sources, that is

$$f = (1 - kh)\, f_1 + khf_2 \tag{1.3}$$

Note that f_2 is actually a function of p_{hj}, which is an unknown parameter in the model. We further used the biallelic SNV datasets from 1000 genome project (http://ftp.1000genomes.ebi.ac.uk/vol1/ftp/data_collections/1000_genomes_project/release/20190312_biallelic_SNV_and_INDEL/) to estimate the empirical distribution of p_{h1}. We use a discrete distribution ranging from 0 to 0.5 to characterize the distribution of p_{h1}, which takes value of 0.05, 0.15, 0.25, 0.35, 0.45 with probability 92.77%, 2.59%, 1.80%, 1.48% and 1.36%, respectively. Combining the uncertainty of heterozygotic rate, the false positive rate is:

$$f = (1-kh)\, f_1 + kh \sum_x f_2(x) P(p_{h1} = x). \tag{0.3}$$

True positive rate (TPR) of identifying MSK

As discussed before, the probability of a k-mer present in more than two-thirds of male individuals is $\sum_{i=\frac{2}{3}n}^{n} C_n^i p_{oj}^i q_{oj}^{n-i}$. As the probability that a k-mer from autosomes is identified as a MSK due to sequencing error is too small, the probability that an MSK is present in none of the females is roughly 1. Then the TPR of identifying MSK is the product of the probability of the two events,

that is

$$\text{TPR} = \sum_{i=\frac{2}{3}n}^{n} C_n^i p_{oj}^i q_{oj}^{n-i}$$

The probability of sorting long reads

For third-generation long reads, the sequencing errors are higher (PacBio CLR or Nanopore) and their lengths vary a lot. Assume a long read contains *N* specific markers, with *N* to be the function of the read length and the distribution of MSK, and the probability that a *k*-mer is correctly sequenced is $p = (1\text{-}r_3)^k$ (r_3 is the sequencing error of long reads). Then the probability that at least *M* MSKs are identified is

$$\sum_{i=M}^{N} C_N^i p^i (1-p)^{N-i}$$

In SRY software, the average number of MSKs (about 7/kb) across the Y chromosome is taken as the value of *M* to sort Y-chromosome long reads.

Assessment

In order to evaluate the MSK identification process of SRY, we firstly used kmer_count to obtain the *k*-mer of all chromosomes of the human T2T genome, and used filterx (https://github.com/ruanjue/filterx) to identify the specific *k*-mer of T2T-Y chromosome, which served as the standard for subsequent evaluations. Then, we used the mason_simulator (v2.0.9) program in the mason[20] package with the parameter (--illumina-prob-mismatch 0.009 --illumina-prob-insert 0.0005 --illumina-prob-deletion 0.0005 --illumina-read-length 150) to generate short-read data for male and female populations using the human T2T genome with or without T2T-Y chromosome as a reference, respectively. We used different seed values for all individuals to avoid the result that the simulated data for all individuals were same. Finally, SRY used these population data to identify MSKs, which were evaluated by comparison with T2T-Y chromosome-specific *k*-mers.

For the theoretical value of the precision of SRY on identifying MSK, we used the following formula:

YSK*TPR/(YSK*TPR + AXK*FPR)

Where YSK represents the specific *k*-mer number of T2T-Y chromosome, AXK represents the *k*-mer number of the human T2T autosomes and X chromosome, and TPR represents the true positive rate and FPR represents the false positive rate of identifying MSK, respectively.

We further simulated 50X Nanopore, PacBio CLR and HiFi reads (25X for Y chromosome) based on the human T2T genomes using badread[21] package (v0.1.3) with the following commands, respectively:

badread simulate --reference human_autoX.fa --quantity 50X --error_model nanopore --start_adapter 0,0 --end_adapter 0,0 --junk_reads 0 --random_reads 0 --chimeras 0 (simulated Nanopore reads of autosomes and X chromosome)

badread simulate --reference human_Y.fa --quantity 25X --error_model nanopore --start_adapter 0,0 --end_adapter 0,0 --junk_reads 0 --random_reads 0 --chimeras 0 (simulated Nanopore reads of Y chromosome)

badread simulate --reference human_autoX.fa --quantity 50X --error_model pacbio --identity 85,95,3 --length 7500,7500 --start_adapter 0,0 --end_adapter 0,0 --junk_reads 0 --random_reads 0 --chimeras 0 (simulated PacBio CLR reads of autosomes and X chromosome)
badread simulate --reference human_Y.fa --quantity 25X --error_model pacbio --identity 85,95,3 --length 7500,7500 --start_adapter 0,0 --end_adapter 0,0 --junk_reads 0 --random_reads 0 --chimeras 0 (simulated PacBio CLR reads of Y chromosome)
badread simulate --reference human_autoX.fa --quantity 50x --error_model pacbio --qscore_model pacbio --identity 99,100,3 --length 12000,12000 --start_adapter 0,0 --end_adapter 0,0 --junk_reads 0 --random_reads 0 --chimeras 0 (simulated PacBio HiFi reads of autosomes and X chromosome)
badread simulate --reference human_Y.fa --quantity 25x --error_model pacbio --qscore_model pacbio --identity 99,100,3 --length 12000,12000 --start_adapter 0,0 --end_adapter 0,0 --junk_reads 0 --random_reads 0 --chimeras 0 (simulated PacBio HiFi reads of Y chromosome)
SRY was assessed for its ability to sort these simulated long reads by considering different TPR values of MSK.
Even the precision of MSK decreases to 70% (including ~7,000,000 MSKs and ~3,000,000 non-MSKs), the density of these non-MSKs on autosomes and X chromosomes was only 1 per kb, which was significantly lower than the threshold set by SRY (7/kb). Therefore, we took the theoretical precision of SRY on sorting long reads as 1. For simplicity, the process of calculating the TPR of SRY on sorting long reads ignored the length distribution of the reads and used the window with 10kb length to calculate the specific *k*-mer distribution of the T2T-Y chromosome.

Y chromosome assembly, identification and evaluation

We collected ~60X ultra-long ONT data and ~35X HiFi data[15], and used SRY for the long-read sorting of the Y chromosome. Due to the abundance of repetitive sequences in the heterochromatic regions of the Y chromosome, the number of available Y-specific markers is limited. Therefore, we used two lengths of *k*-mers (*k*=21 and *k*=51) for the sorting of HiFi reads. Verkko (v1.0)[15] was used to assemble the selected data with parameters (-d Asm --hifi hifi.sorted.fq.gz --nano ul-ont.sorted.fq.gz --threads 128). We compared the assembly results of Verkko in trio mode (collected from ref.[15]) and SRY mode to T2T-CHM13 using the Quast (v5.0.2)[28] software. The file with the suffix name "coords.filtered" was used by DotPlotly (parameters: -slt -m 100 -q 100) (https://github.com/tpoorten/dotPlotly) to generate the alignment plot.

We collected the dataset of a trio family including short reads from father (HG01107, ~113X) and mother (HG01108, ~79X) and Nanopore reads from child (HG01109, ~72X)[29]. SRY separated 1.3G (~25X) long reads of HG01109 using MSK markers identified from HX1. Then, we used wtdbg2.5[26] with parameters “-L 0 -p 0 -k 21 -s 0.25 -S 2 --rescue-low-cov-edges” to assembly those long reads. The remaining long reads were assembled by wtdbg2.5 with parameters “-x ont -g 3g” and polished with the program wtpoa-cns in wtdbg2.5. All of the assembled contigs were further polished with wtpoa-cns using short reads. Sorting of long reads and genome assemblies for other nine individuals were performed the same way. Trio binning phased HG01109 long reads using with command “canu -stopAfter=haplotype genomeSize=3g -haplotypeMale HG01107.fastq.gz -haplotypeFemale HG01108.fastq.gz -nanopore-raw HG01109.fasta.tar.bz2”. Wtdbg2 with the parameters (-g 3.1G -x ont) was applied to assembly phasing reads from trio binning and perform whole genome assembly for WGS. SRY was then

used to partition candidate contigs of Y chromosome for trio binning and WGS. We utilized quast (v5.0.2)[28] with default parameters to evaluate the assembled genome quality.

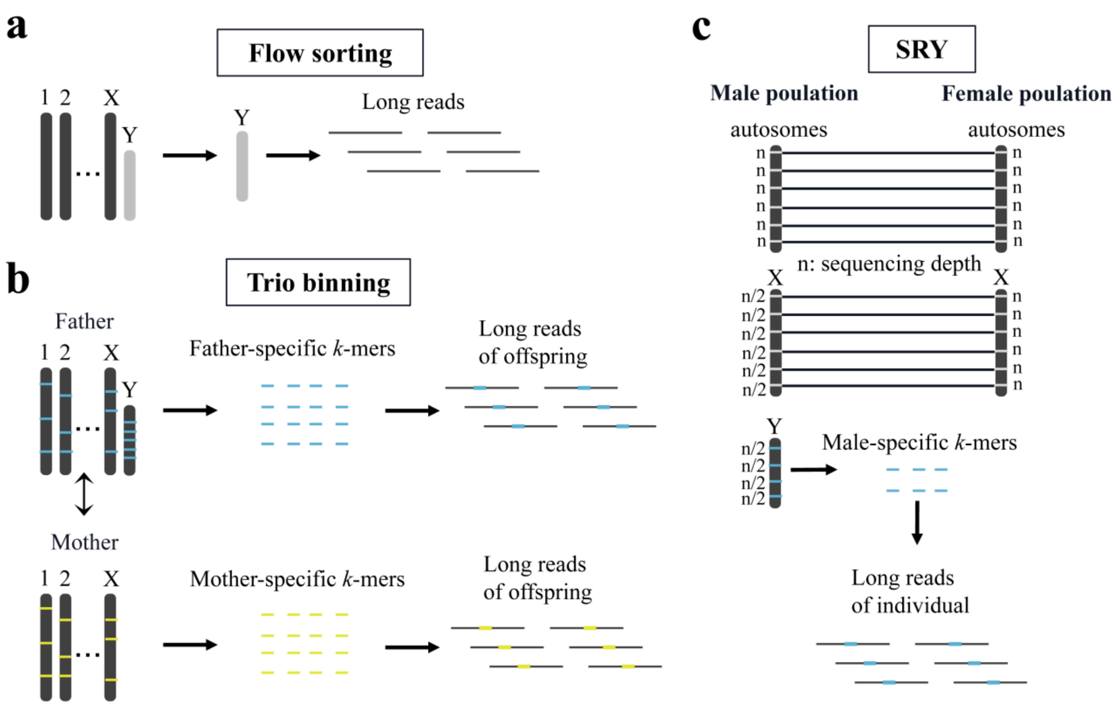

Fig. 1: Overview of three methods for sorting long reads. a Flow-sorting is an experimental method for separating Y chromosome. **b** Trio binning compares *k*-mers from short reads of parent genomes and identifies father-specific and mother-specific *k*-mers, respectively. These specific *k*-mers are used to bite long reads for each parent. **c** SRY rules out *k*-mers presenting in both male and female populations and retains *k*-mers only occurred in male population with half sequencing depth. SRY utilizes these male-specific *k*-mers to separate long reads of Y chromosome.

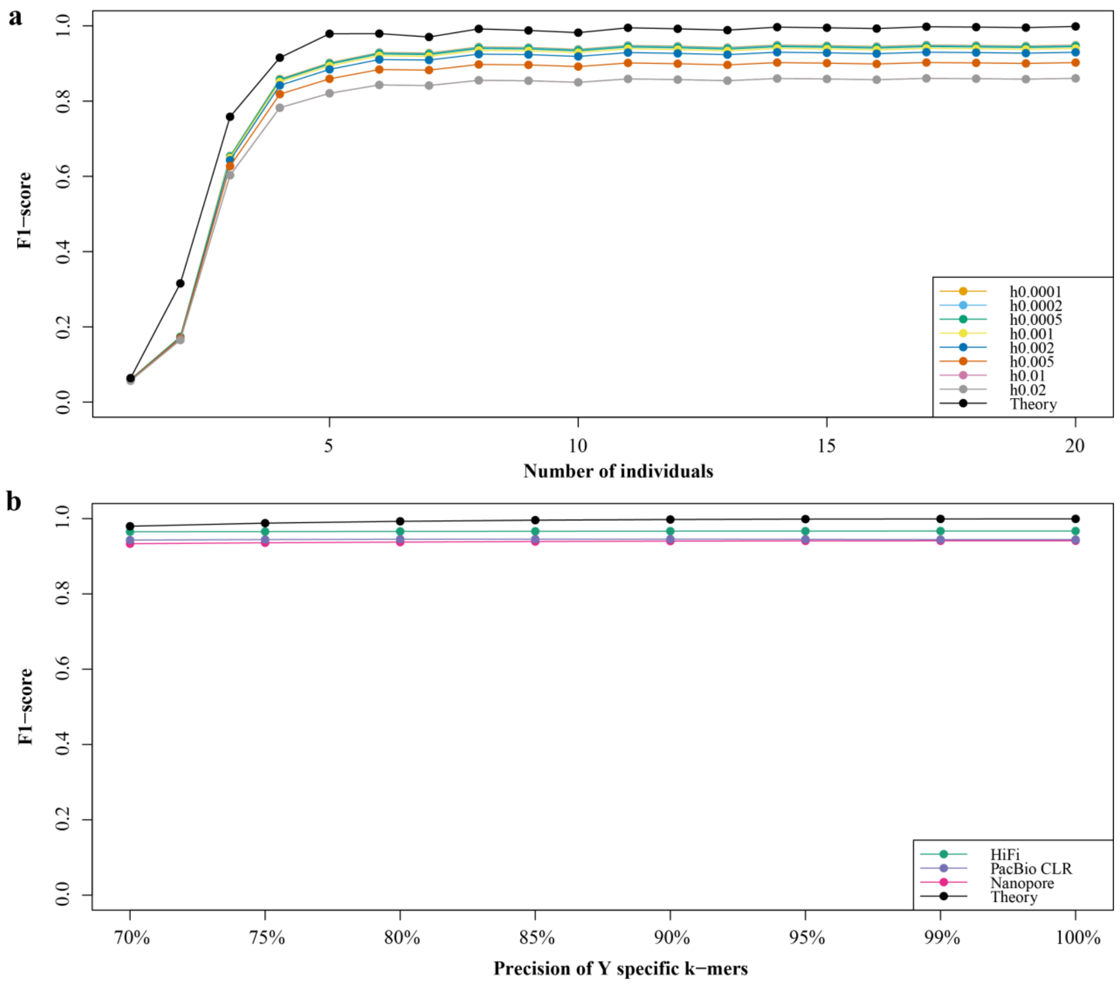

Fig. 2: Theoretical model and performance of SRY on simulated data. a The F1-score of SRY on identifying male-specific *k*-mers with different individual number (per sex) and population heterogeneity, respectively. For simplicity, we did not consider the similarity distribution between the Y chromosome and other chromosomes in the calculation of the theoretical value. **b** The F1-score of SRY on sorting HiFi, PacBio CLR and Nanopore long reads. The combination of length distribution and specific *k*-mers distribution can cause TPR differences between theoretical and simulated data as well as TPR differences within simulated data.

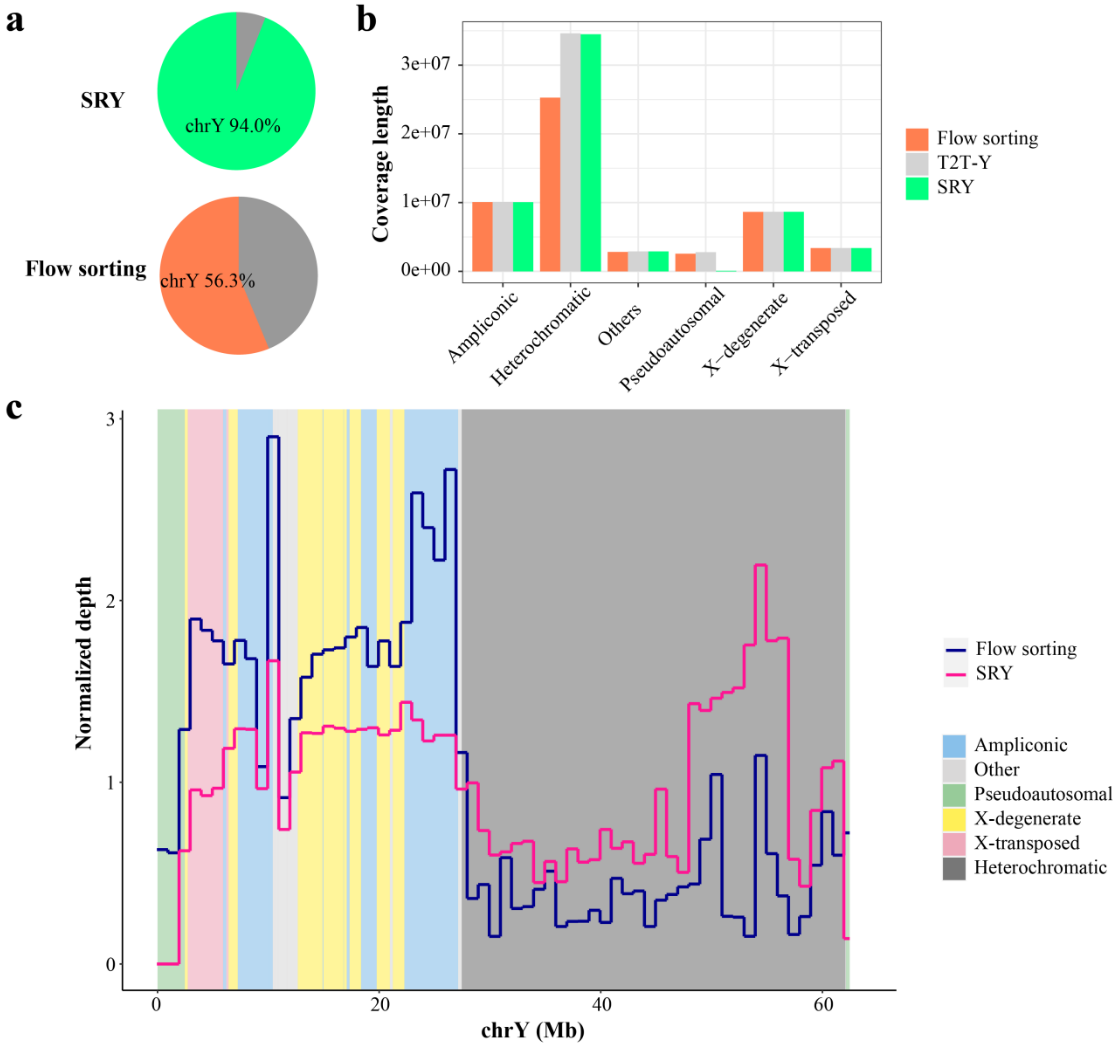

Fig. 3: Comparison of sorting results between SRY and flow sorting. a Alignment distribution of sorted reads for SRY and flow sorting. The alignment on autosomes and X chromosome is colored in gray. **b** The coverage of sorting reads by the two methods on discrete regions of Y chromosome. SRY aims to separate male-specific long reads, so the coverage is lower on pseudoautosomal region where recombination events occur frequently between X and Y chromosome. **c** The normalized depth of long reads separated by the two methods. The colored rectangles represent discrete regions on the T2T-Y chromosome.

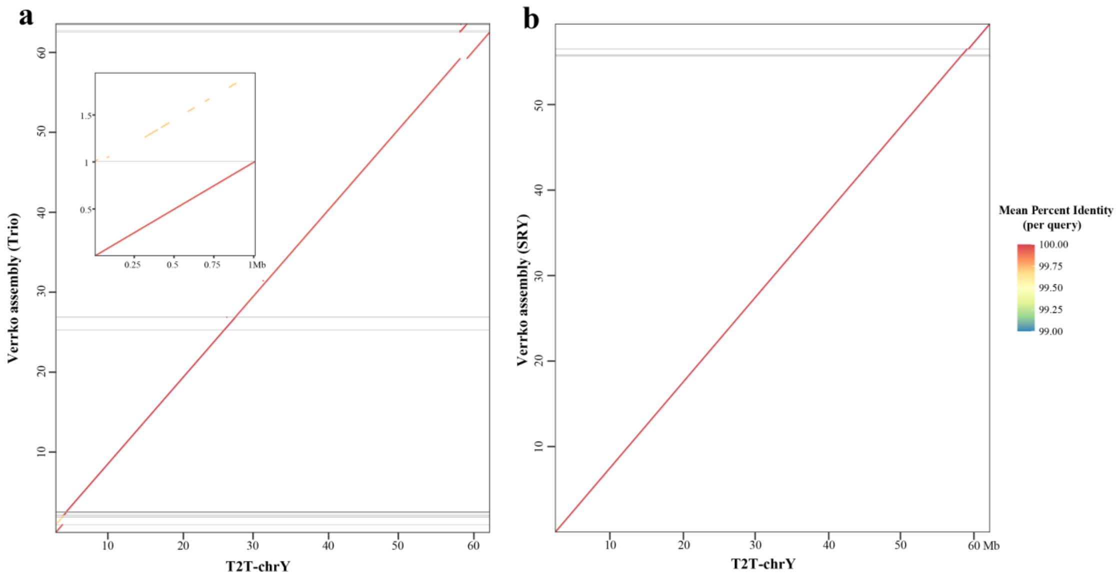

Fig. 4: Verkko assembly using Trio or SRY. The x axis represents the complete Y chromosome assembled by T2T consortium, and y axes represent assemblies by verkko with Trio (**a**) and SRY (**b**), respectively. Inset in (a) represents an enlarged view of the first 1 Mb region of the T2T-chrY. The diagonal colored with identity shows the alignment result. (Note: T2T-Y palindrome P5 (18483410-19235627) is expected to be inverted, and this error was not resolved by the assembly using verkko SRY mode.)

Reference

1. Tomaszkiewicz, M., Medvedev, P. & Makova, K.D. Y and W Chromosome Assemblies: Approaches and Discoveries. *Trends. Genet.* **33**, 266-282 (2017).
2. Bellott, D.W. et al. Avian W and mammalian Y chromosomes convergently retained dosage-sensitive regulators. *Nat. Genet.* **49**, 387-394 (2017).
3. Skaletsky, H. et al. The male-specific region of the human Y chromosome is a mosaic of discrete sequence classes. *Nature* **423**, 825 (2003).
4. Hughes, J.F. et al. Chimpanzee and human Y chromosomes are remarkably divergent in structure and gene content. *Nature* **463**, 536-539 (2010).
5. Hughes, J.F. et al. Strict evolutionary conservation followed rapid gene loss on human and rhesus Y chromosomes. *Nature* **483**, 82-86 (2012).
6. Soh, Y.Q.S. et al. Sequencing the Mouse Y Chromosome Reveals Convergent Gene Acquisition and Amplification on Both Sex Chromosomes. *Cell* **159**, 800-813 (2014).
7. Dolezel, J. et al. Chromosomes in the flow to simplify genome analysis. *Funct. Integr. Genomics* **12**, 397-416 (2012).
8. Kuderna, L.F.K. et al. Selective single molecule sequencing and assembly of a human Y chromosome of African origin. *Nat. Commun.* **10**, 4 (2019).
9. Akagi, T., Henry, I.M., Tao, R. & Comai, L. A Y-chromosome–encoded small RNA acts as a sex determinant in persimmons. *Science* **346**, 646-650 (2014).
10. Carvalho, A.B. & Clark, A.G. Efficient identification of Y chromosome sequences in the human andDrosophilagenomes. *Genome Res.* **23**, 1894-1907 (2013).
11. Muyle, A. et al. SEX-DETector: a probabilistic approach to study sex chromosomes in non-model organisms. *Genome Bio. Evol.* **8**, 2530-2543 (2016).
12. Koren, S. et al. De novo assembly of haplotype-resolved genomes with trio binning. *Nat. Biotechnol.* **36**, 1174-1182 (2018).
13. Rhie A, Nurk S, Cechova M, et al. The complete sequence of a human Y chromosome. *Nature*, 2023, 621(7978): 344-354.
14. Cheng H, Concepcion G T, Feng X, et al. Haplotype-resolved de novo assembly using phased assembly graphs with hifiasm. *Nat Methods*, 2021, 18(2): 170-175.
15. Rautiainen, M. et al. Telomere-to-telomere assembly of diploid chromosomes with Verkko. *Na. Biotechnol*. https://doi.org/10.1038/s41587-023-01662-6 (2023).
16. Serra Mari R, Schrinner S, Finkers R, et al. Haplotype-resolved assembly of a tetraploid potato genome using long reads and low-depth offspring data. bioRxiv, 2022.
17. Rangavittal, S. et al. DiscoverY: a classifier for identifying Y chromosome sequences in male assemblies. *BMC genomics* **20**, 1-11 (2019).
18. Rangavittal, S. et al. RecoverY: k-mer-based read classification for Y-chromosome-specific sequencing and assembly. *Bioinformatics* **34**, 1125-1131 (2018).
19. Hall, A. B. et al. Six novel Y chromosome genes in Anopheles mosquitoes discovered by independently sequencing males and females. *BMC genomics* **14**, 1-13 (2013).
20. Holtgrewe M. Mason–a read simulator for second generation sequencing data. *Technical Report FU Berlin*, 2010.
21. Wick R R. Badread: simulation of error-prone long reads. *JOSS.* 2019; 4(36): 1316.
22. Shi, L. et al. Long-read sequencing and de novo assembly of a Chinese genome. *Nat Commun.* **7** (2016).

23. Liu, Q. et al. Detection of DNA base modifications by deep recurrent neural network on Oxford Nanopore sequencing data. *Nat. Commun.* **10**, 2449 (2019).
24. Lan, T. et al. Deep whole-genome sequencing of 90 Han Chinese genomes. *GigaScience* **6** (2017).
25. Li, H. Minimap2: pairwise alignment for nucleotide sequences. *Bioinformatics* **34**, 3094-3100 (2018).
26. Ruan, J. & Li, H. Fast and accurate long-read assembly with wtdbg2. *Nat. Methods* **17**,155-158 (2020).
27. Gong, G. et al. Origin and chromatin remodeling of young X/Y sex chromosomes in catfish with sexual plasticity. *Natl Sci Rev.* 10, nwac239 (2023).
28. Gurevich, A., Saveliev, V., Vyahhi, N. & Tesler, G. QUAST: quality assessment tool for genome assemblies. *Bioinformatics* **29**, 1072-1075 (2013).
29. Shafin, K. et al. Nanopore sequencing and the Shasta toolkit enable efficient de novo assembly of eleven human genomes. *Nat Biotechnol.* (2020).

Data availability

We downloaded all Nanopore, PacBio and Illumina datasets under NCBI project number PRJNA301527 for HX1. The SRA numbers of Han Chinese population were listed at supplementary table 1 and 2. The trio family (HG01107, HG01108 and HG01109), HG005, HG006, HG01243, HG02055, HG03098 and HG03492 reads are available at https://s3-us-west-2.amazonaws.com/human-pangenomics/index.html. Short reads as well as PacBio and/or Nanopore long reads of HG002 and HG003 are available at https://ftp-trace.ncbi.nlm.nih.gov/ReferenceSamples/giab/data/AshkenazimTrio/. We also downloaded ERR3241824 for HG01107 and ERR3241825 for HG01108 to improve the performance of trio binning. All the assembling results have been submitted to figshare (https://doi.org/10.6084/m9.figshare.14564484).

Code availability

The SRY source code is hosted by GitHub at: https://github.com/caaswxb/SRY.

Acknowledgments

This work was supported by National Natural Science Foundation of China (Grant No. 91731304 to J.R.), National Key Research and Development Program of China (Grant No. 2019YFA0707003 to J.R.) and National Natural Science Foundation of China (Grant No. 31860638 to Q. L.). We thank S. Wu from CAAS for his suggestions on genome assembly. We thank High-performance Computing Center of Agricultural Genomics Institute at Shenzhen, China Academy of Agricultural Sciences.

Author contributions

J.R. and Q. L. designed the project, and J.R. managed the project. X.W., J.R. and A.L. developed the SRY method. T.Z. constructed the theory model. X.W. and H.L. collected genomic data, performed analysis and wrote the paper. J.R., H.Z. and Y.Z. revised the manuscript.

Competing interests

The authors declare no competing interests.

Figure3

Click here to access/download;Figure;figure3.pdf

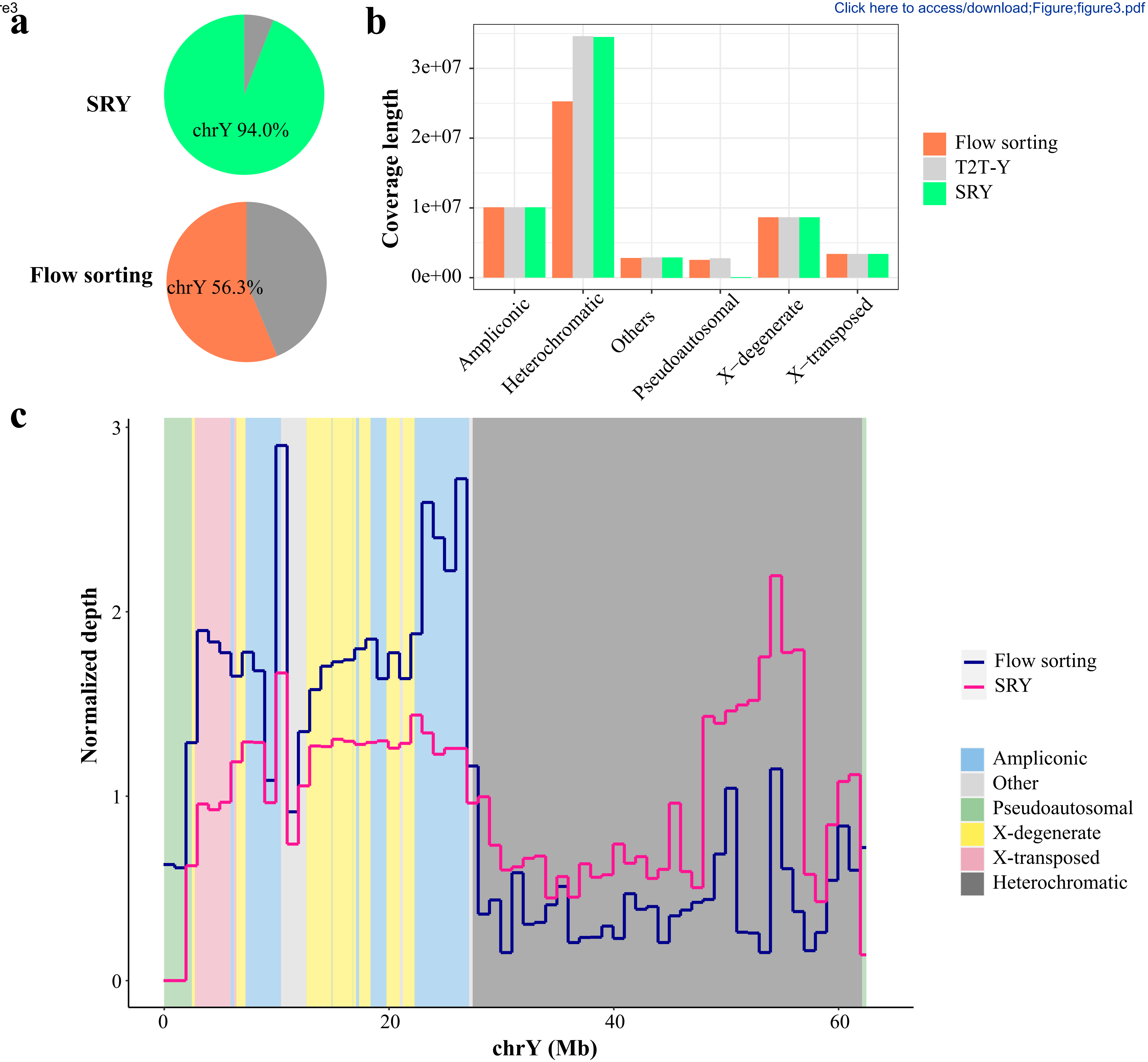

Figure2

Click here to access/download;Figure;figure2.pdf

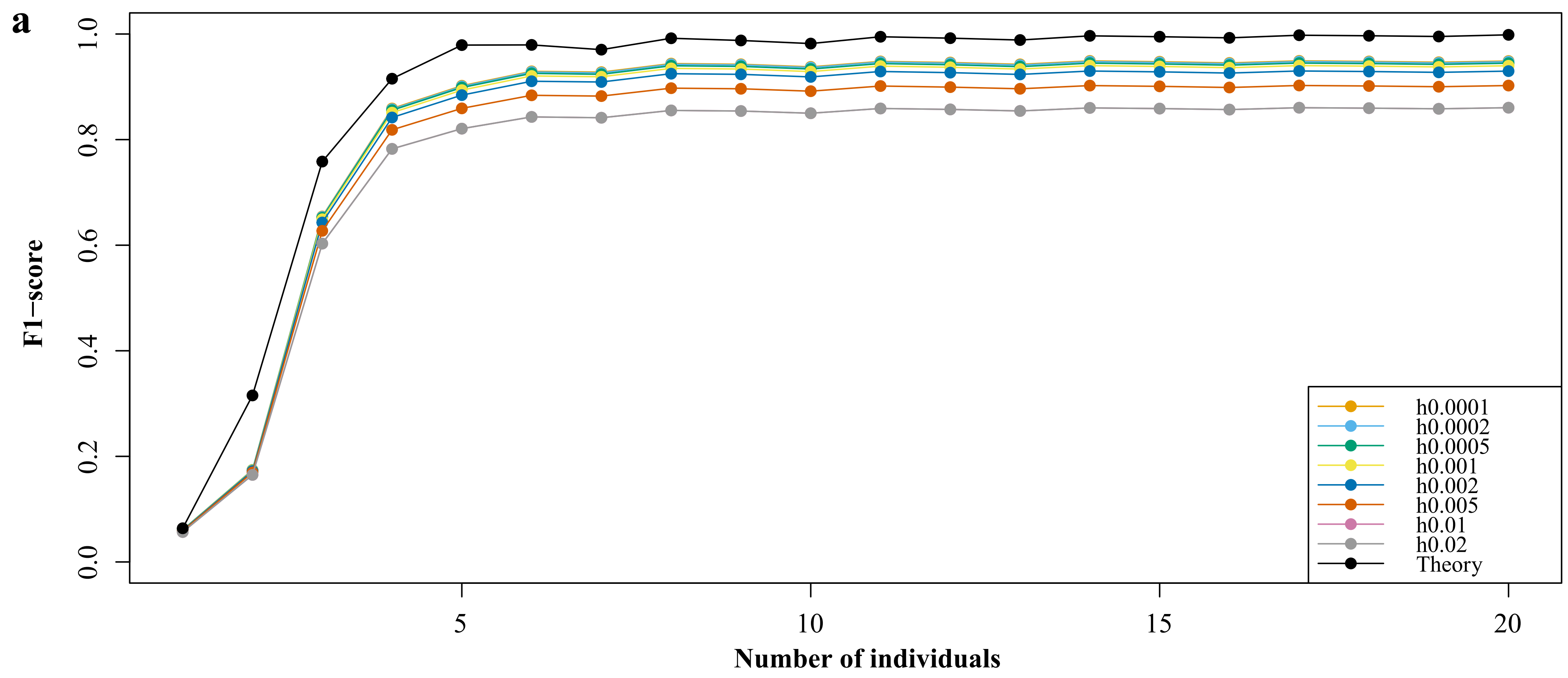

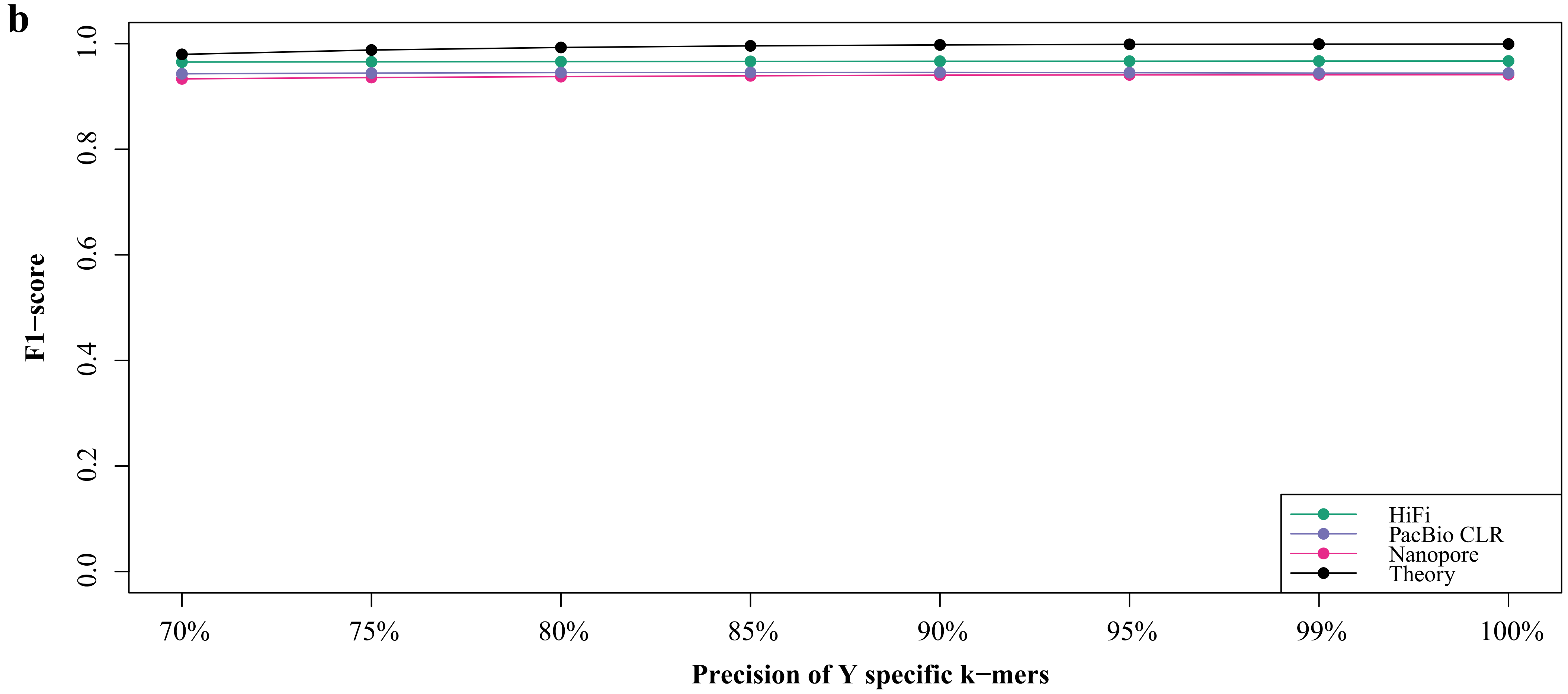

Click here to access/download;Figure;figure1.pdf

Figure1

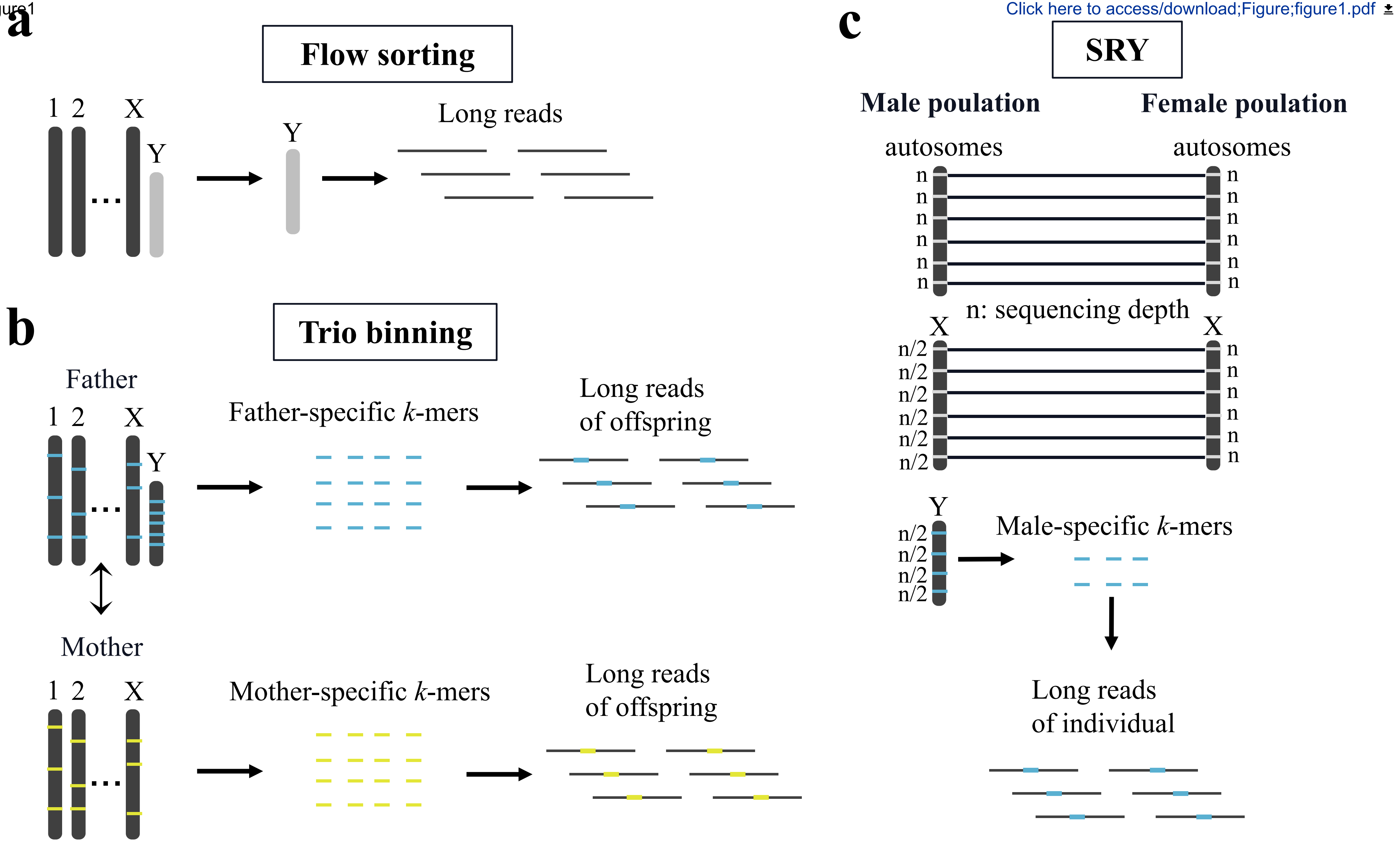

Figure4

Click here to access/download;Figure;figure4.pdf

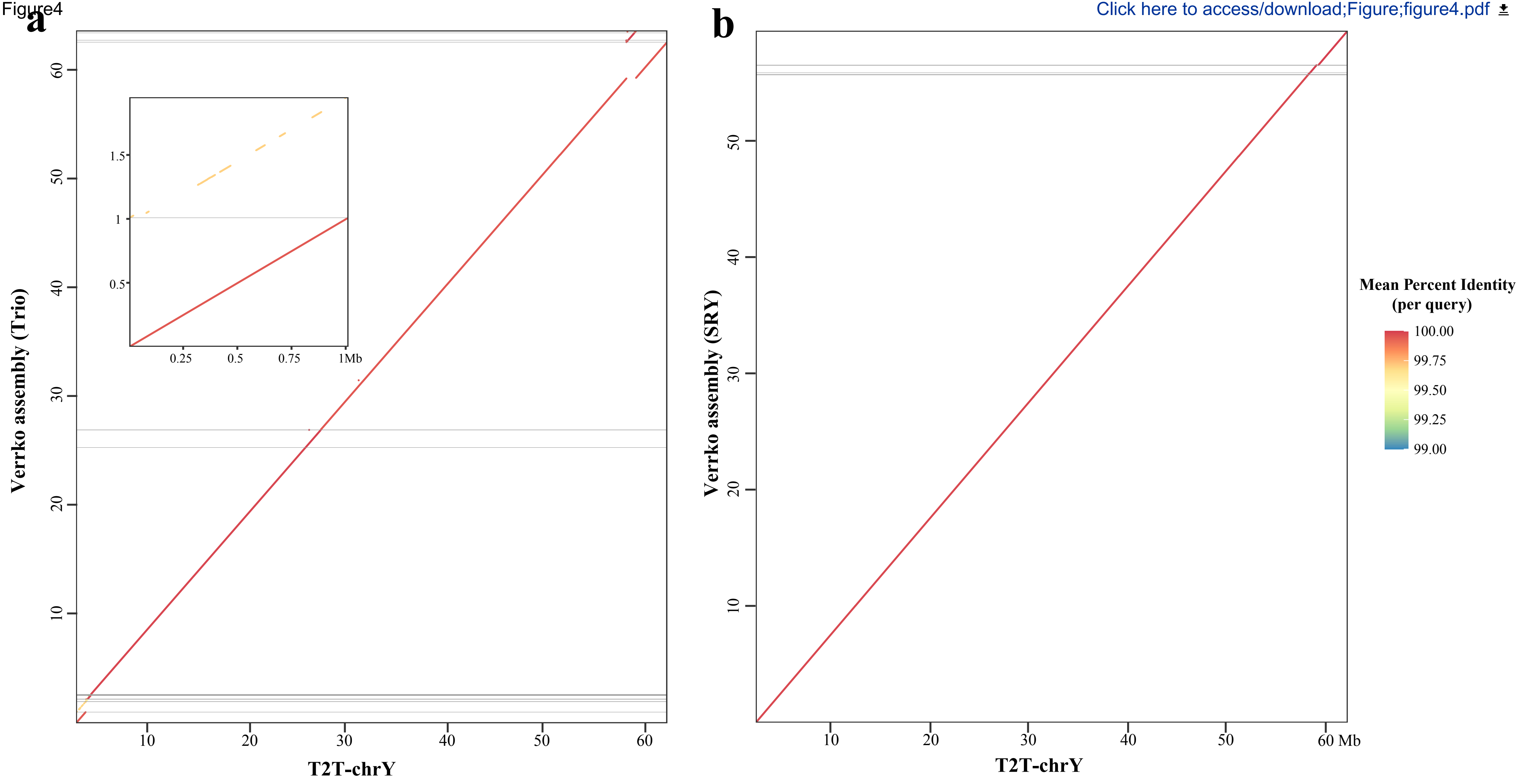

Supplemental

Click here to access/download
Supplementary Material
Supplemental-r.docx

Cover letter

Click here to access/download;Personal Cover;CV-20240117.docx

Dear Hans,

I am writing to submit a revised version of our manuscript entitled “An effective strategy for assembling the sex-limited chromosome”. We would like to thank the reviewers for their insightful comments and constructive feedback.

In response to the reviewers' comments, we have carefully revised the manuscript to address all their concerns. Specifically, we have focused on incorporating the suggestions from the reviewers and addressing the issues raised in Reviewer 3's comment #6. We have included a detailed explanation of our approach in the revised manuscript, which we hope will clarify any confusion that may have arisen.

We believe that these changes have significantly improved the quality of our manuscript, and we hope that you will find it suitable for publication in *GigaScience*.

Thank you for considering our revised manuscript.

Sincerely,
Xiaobo Wang

Response to reviewers

Reviewer 1:

The authors have introduced a novel bioinformatic approach for sex chromosome assembly, addressing a persistently challenging problem in genomics. This method harnesses the full potential of whole-genome resequencing data without necessitating supplementary experimental procedures, rendering it applicable to a wide array of non-model species. Notably, the method exhibits robustness when applied to human data, surpassing established techniques such as flow-sorting and trio-binning. While the manuscript exhibits promise, several key aspects warrant refinement and elucidation to bolster its consideration for publication in GigaScience.
Response: Thank you for your insightful comments on our manuscript. We appreciate your recognition of the novelty of our bioinformatic approach for sex chromosome assembly and its potential impact on genomics research. We have provided a detailed response to each of your suggestions below.

1. Language Polishing: A degree of language refinement is advisable to enhance the overall clarity and professionalism of the manuscript.
Response: Thank you for your feedback. We have carefully revised the manuscript based on your suggestions as well as those of other reviewers. The modified sections are indicated in blue font.

2. Y Chromosome Assembly Discrepancy: The authors should acknowledge and provide an explanation for the substantial difference between the length of the latest Y chromosome assembly from T2T (~62Mb) and the assembly from SRY with Verkko (~23Mb), as detailed in Table 1.

Response: Thank you for your comment. The Flow sorting method was used to measure only Nanopore data. In order to make a fair comparison with the Flow sorting method, we selected only Nanopore or PacBio data from 10 individuals, which resulted in an assembly of only around 23Mb. We have already mentioned this limitation in the Discussion section of the manuscript.

In order to obtain a complete Y chromosome assembly and to compare with the trio mode of the verkko software, we used long-read Nanopore and HiFi data, which ultimately resulted in a ~60Mb assembly (Figure 4, SRY with Verkko) .

Therefore, the difference in assembly size is mainly due to data sources.

3. Y Chromosome Completeness: In cases where the Y chromosome assembly is incomplete, the inclusion of a figure or table delineating the proportion that SRY can recover in distinct regions of the Y chromosome would be beneficial. This could facilitate a comparative analysis of the method's efficacy across different regions.

Response: Thank you for the clarification. I apologize for any confusion caused. As mentioned in the previous response, we were able to obtain an almost complete Y chromosome assembly using long-read Nanopore and HiFi data. However, for the purpose of a fair comparison with the Flow sorting method, we obtained an incomplete assembly and displayed the assembly statistics for different regions in Table 1. To focus our discussion on the complete Y chromosome assembly, we have made the decision to present Table 1 as a supplementary material.

4. Figure 4 Clarity: It is imperative to label the coordinates on both the X and Y axes in Figure 4 to enhance clarity. While Figure 4 suggests that the assembly from SRY is complete compared to T2T-CHM13, the total length of the SRY assembly (approximately 23Mb) should be clearly reconciled with this observation.

Response: Thank you for your feedback. We have updated the figure to include clear coordinate labels on both axes to enhance clarity (below).

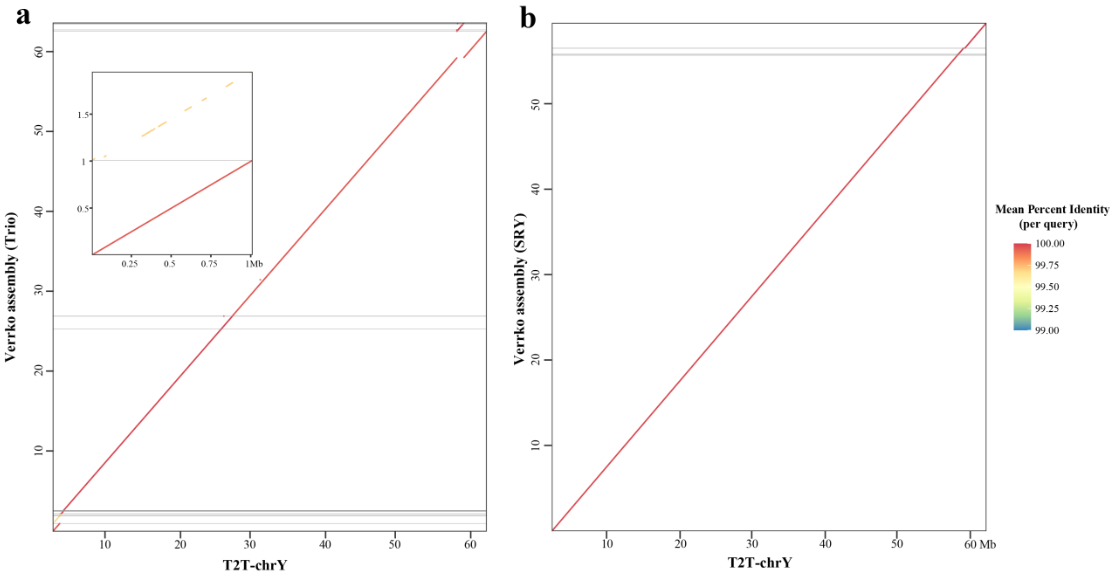

Fig. 4: Verkko assembly using Trio or SRY

5. Table 1 Organization: The organization of Table 1 should be improved to enhance readability and comprehensibility.

Response: Thank you for your suggestion. We have made improvements to enhance the readability and comprehensibility of Table 1. We have split it into two separate tables, labeled as Supplementary Table 4 and Supplementary Table 5, which are included as supplementary material. Furthermore, we have removed some less relevant information from the tables to ensure clarity and focus on the key details.

6. MSK-Based Read Filtering: Authors should explicitly address the potential exclusion of reads from Y regions with lower than average MSK, especially in species with both young and old parts on Y chromosomes. If possible, provide recommendations or strategies for rescuing such reads.
Response: Regarding the potential exclusion of reads from Y regions with lower than average MSK (Male Specific K-mer), our intention in using average MSK as a threshold is to reduce false positives caused by sequencing errors or other factors.

In the case of humans, the differentiation time between the Y and X chromosomes is estimated to be around 300 million years ago (old Y). In our human simulated dataset (~25X coverage for the Y chromosome), we successfully captured approximately 24X (95.6%) of the Y chromosome using HiFi data and approximately 23X (91.5%) using Nanopore data.

For yellow catfish (detailed in Response to Q7), which contains a young Y chromosome with a similarity of over 99% to the X chromosome, we simulated 50X coverage of HiFi data and 50X coverage of ultra-long Nanopore data. Using SRY, we captured approximately 46X (92.2%) of the Y chromosome with HiFi data and approximately 37X (73.2%) with ultra-long Nanopore data.

Therefore, for Y chromosomes that contain both old and young parts, we recommend prioritizing HiFi data as the loss of data will have minimal impact on assembly. If feasible, we also suggest including ultra-long Nanopore data to increase the completeness of the assembly results.

7. Simulation for species with young sex chromosomes: It is essential to conduct additional simulations for testing the efficiency of isolating Y reads for species with young sex chromosomes. This analysis should consider the variation between X and Y chromosomes, aiding researchers in evaluating the method's suitability for their specific study organisms.
Response: We have conducted simulations to test the efficiency of isolating Y reads for species with young sex chromosomes, using the yellow catfish as a model organism [1]. This species contains a young Y chromosome with a size of approximately 43.2 Mb, which shares greater than 99% similarity with the X chromosome. In our simulations, we generated 100X HiFi and Nanopore data (50X for the Y chromosome) and obtained 46X of HiFi and 37X of ultra-long Nanopore data after sorting for the Y chromosome. Using the verkko software, we assembled the Y chromosome into two contigs with a total size of 42.7 Mb. These results demonstrate that our method is effective for assembling the Y chromosome of species with young sex chromosomes.We have incorporated these results into our manuscript.

[1] Gong G, Xiong Y, Xiao S, et al. Origin and chromatin remodeling of young X/Y sex chromosomes in catfish with sexual plasticity. *National Science Review*, 2023, 10(2): nwac239.

Addressing these points will further strengthen the manuscript's scientific rigor and its suitability for publication in GigaScience.

Response: Thank you for your valuable feedback.

Reviewer 2:

The SRY method, developed and evaluated for sorting long reads of sex-limited chromosomes, has shown promise in effectively identifying and sorting sequences based on sex-specific markers, particularly the Y chromosome. These sorted long reads are then utilized for genome assembly. Additionally, the SRY method can be used to select Y chromosome contigs from a male individual's whole genome assembly.Overall, the success of SRY in sorting and assembling long reads of sex-limited chromosomes highlights its potential as an alternative to experimental methods for studying sex-specific genomic regions.

Response: Thank you for your positive comments.

Here are some comments for further improvement of manuscript:

1) The authors may want to consider to presenting a table for standard evaluation metrics (k-mer or alignment-based). See Garg 2021 (https://genomebiology.biomedcentral.com/articles/10.1186/s13059-021-02328-9).

Response: Thank you for your suggestion. Table 1 presents the standard evaluation metrics for genome assembly using quast, an alignment-based software that is widely used for assessing genome assembly results. However, we have made some changes based on the feedback from other reviewers. The updated Table 1 has been moved to supplementary materials and is now labeled as Supplementary Table 4 and 5. We believe that these optimizations enhance the presentation of our results.

2) Adding a few important genes that are medically relevant and assembled properly may further add value to the work.

Response: In our study, we found that the assembly of gene sequences is generally not complex and can be relatively straightforward. Using the Verkko assembly method, both the direct assembly and the assembly after SRY sorting yielded comparatively complete genes on the Y chromosome. However, we observed that the assembly after sorting improved sequence contiguity, which is beneficial for analyzing regulatory sequences near the genes. We acknowledge that further investigation is needed to explore this aspect in future research. We appreciate your feedback.

Reviewer 3:

1.In the introduction, add recent marker based graph phasing algorithms in long-reads, such as hifiasm trio and verkko trio mode after the T2T-Y. They are different from trio-binning, which tries to phase the reads upfront. Graph based phasing is using markers to determine haplotype specific paths to traverse.

Response: Thank you for your feedback. In the introduction, we include recent marker-based graph phasing algorithms in long-reads, such as hifiasm trio and verkko trio mode, after discussing T2T-Y.

a. T2T-Y chromosome should be referencing Rhie et al., Nature 2023. Verkko is a successor of the manual efforts taken in T2T-Y, which should be also noted in the introduction.

Response: We have made the necessary updates to reference Rhie et al., Nature 2023 as the source for the T2T-Y chromosome. Additionally, we have included a note in the introduction to acknowledge that

Verkko is a successor of the manual efforts undertaken in T2T-Y.

b. Reference for sexPhase program is still missing. Also, some rephrasing of the sentence is needed, as the way it is currently written is easily misleading to be understood as sexPhase was part of the methods used in the assembly of the T2T-Y.

Response: Thank you for pointing that out. The reference for the sexPhase program is a article titled "Genomes of the Banyan Tree and Pollinator Wasp Provide Insights into Fig-Wasp Coevolution," published in *Cell*, 2020. This software was specifically used for the assembly of the fig tree sex chromosomes mentioned in the article and does not have general applicability. Therefore, it is not suitable to be included as the latest development after T2T-Y. We have removed this content and added a description of marker-based graph phasing algorithms instead.

2. There are other approaches for phasing genomes taken in plants, for example the poly ploid potato phasing using many siblings of the child by Mari et al. bioRxiv 2022.

Response: Thank you for your suggestion. We have incorporated the information into the Introduction section of our paper.

3. "But only one male and one female could suffer from sampling error" - this part is unclear. Please clarify.

Response: We apologize for the unclear wording in the previous version. We have since made revisions to the manuscript and have updated this section to read: "By incorporating population data into our analysis, we anticipate a reduction in the impact of sequencing coverage and allelic genotype variations when identifying Y chromosome-specific markers in a comparative analysis involving only two individuals of different genders." We hope that this revised statement is clearer and more accurately reflects our intended meaning.

4. Reference for the mason_simulator, badread software is missing.

Response: Corrected.

5. Provide the accession (HG02982) for the "African human Y" in the main text.

Response: Accession (HG02982) for "African human Y" has been added now.

6. I appreciate that the authors compared assemblies to T2T-Y as I requested before. However, fundamentally, mapping to T2T-Y and comparing length of each sequence classes is comparing apples to oranges, particularly in the heterochromatic region and ampliconic region of the Y. It is known to have variable copy numbers and size differences between two individuals. Frequent inversions have been reported in the ampliconic regions across different Y haplogroup. The number, size, and distribution of the repeat arrays composing the heterochromatic region has been shown to vary among different Y haplogroups in Hallast et al., Nature 2023. This can be also seen in Fig. 3c; the overall depth of the flow sorting in the heterochromatic region is below 1 - indicating the Yqh is shorter than T2T-Y, as it is in Fig. 3b. To make the benchmark legit, the authors should compare SRY and the flow sorting method using samples from the same individual. HG02982 and HX1 are presumably having very different sequence compositions given the diverged population history (African vs. Asian). Comparing total length of the assembled region against a 3rd different Y haplogroup (HG002Y) makes

things more complicated, especially on regions that are known to vary a lot. If the authors think flow sorting based method needs to be compared, it should be benchmarked on the same individual to make an apple-to-apple comparison. I do agree results from read sorting (i.e. portion of reads sequenced from non-Y chromosomes in SRY vs. flow-sorting) is an important finding. However, I'd still argue comparing assemblies from the two different Y haplogroups is a stretch. The authors could have performed the same assembly length comparison on the T2T-Y using results from their SRY sorted reads with Verkko of HG002 vs. Verkko assembly using trio-binned markers.

Response: We completely agree with your points. As you mentioned, the comparison results from read sorting are indeed important findings. We acknowledge that using a different Y haplogroup for assembly length comparison may not be ideal. In our study, we did not have access to publicly available HG02982 whole genome long-read data, so we could only compare the SRY sorting results from another sample (HX1) to the flow sorting results. As a methodological study, we currently do not have the capability to collect HG02982 samples and perform third-generation sequencing. Even if we were able to obtain HG02982 samples, there could be inconsistencies in sequencing data length and accuracy due to updates in sequencing platforms, which would compromise the fairness of the comparison of the sorting results. However, we have taken your suggestion and compared and analyzed the sorting results of HG002 in the subsequent results section. In addition to your valuable feedback, we have also taken into consideration other suggestions from the reviewers. As a result, we have decided to move the Table 1 to the supplementary materials (Supplementary Table 4-6) to shift the focus on the assembly results of the complete Y chromosome. Thank you for your constructive comments, and we hope that the revised manuscript will meet your expectations.

7. In the section where assemblies are compared, the authors point to Table 1, which contains results from HG01109. HG01109 has never been mentioned before. I thought the authors were comparing assemblies from SRY sorted reads of HX1? I am not sure why the authors suddenly added a 3rd PUR genome with no context. Was this a mistake? Add results from HX1 to Table 1.

Response: We apologize for any confusion caused. The population data we collected for our study is representative of the Chinese population, and the long-read sorting data mentioned, HX1, is an individual of Chinese. In order to compare the assembly results with the trio binning method, we included the sample HG01109, which is part of a trio dataset consisting of the parental HG01107 and HG01108, along with their offspring HG01109. To clarify this in the manuscript, we have added the following explanation: "We collected a trio dataset consisting of the parental genomes HG01107 and HG01108, as well as the offspring genome HG01109, in order to perform trio binning and compare the resulting assemblies of HG01109." We have now included the results from HX1 in Supplementary Table 6 for a more clear presentation of the comparisons.

8. Please add divider lines in Table 1 between All / Ampliconic / X-degenerate / X-transposed / PAR / Het / Others. It is hard to see which rows belong to which category.

Response: Corrected in Supplementary Table 4-6.

9. The last result section where authors compare results from Verkko, it is unclear how the verkko assembly was run. The authors say "default option", and later "in trio mode" in the methods. Did the authors collect parental reads from HG002 (HG003 and HG004)? How was "trio mode" performed? Did the authors used trio binning to sort the reads, then run Verkko? Or used the homopolymer

compressed parental kmers and used that in the Rukki step of Verkko (and this should be benchmarked)? Was the HG002 trio assembly taken from Rautiainen et al. paper? Please clarify and add the missing parts to the main text and methods.

Response: We apologize for the lack of clarity in our manuscript regarding the Verkko assembly. To clarify, we ran the Verkko assembly with the following parameters: "-d Asm --hifi hifi.sorted.fq.gz --nano ul-ont.sorted.fq.gz --threads 128". The HG002 trio assembly used in our study was obtained from the Rautiainen et al. paper. We have updated the methods to include this information.

10. Related to the above section, it is hard to see in Fig. 4a the "two approximately 1 Mb contigs aligning to the same region of the Y chromosome". An enlarged inset of the dotplot may be helpful. Also, add legends and scale to the X and Y axis of the dotplots.

Response: We have updated Figure 4a to include an enlarged inset of the approximately 1 Mb region, and added legends and scale to both X and Y axes of the dotplot. Please see the Fig.4 below:

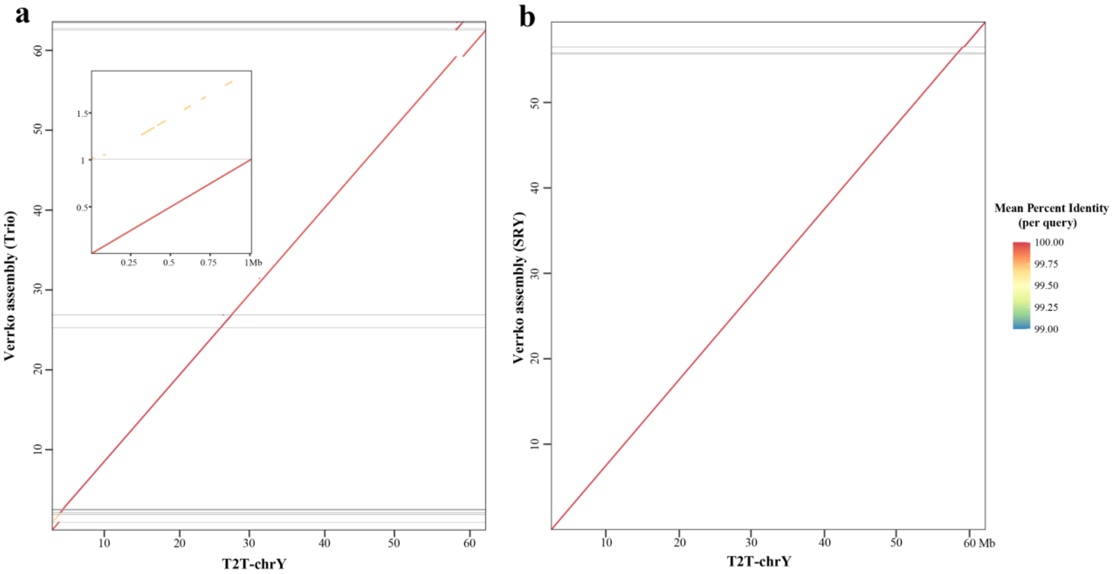

11. Note there is a mis-assembly reported on T2T-Y palindrome P5 (https://github.com/marbl/CHM13-issues/blob/main/v2.0_issues.bed), which the entire P5 should be inverted. I don't see this in the dotplots of Fig. 4.

Response: Thank you for bringing this to our attention. Upon reviewing the alignment of T2T-Y palindrome P5 (18483410-19235627), we have identified an approximately 3.5kb inversion (18857803-18861259) in the assembly generated by the verkko sry mode. Due to its relatively short alignment length, this inversion may not be clearly visible in the dotplots of Figure 4. Additionally, we have performed assembly using hifiasm in the P5 region and did not find any inversions. This indicates that the current approach (SRY) of read sorting may not effectively resolve the situation with P5. We have made a note of this in Figure 4 to provide clarification.

12. In the discussion, the authors are mentioning results from the 10 trios that have been removed from the previous results. Please add the 10 trio results to the main text if it was a mistake, or remove the irrelevant results from the Discussions and Supp. Tables.

Response: We apologize for any confusion caused. In our study, we collected data from 10 individuals, out of which only HG01109 had trio data. The previous removal referred to the pan-genome analysis of

these 10 individuals. The assembly results of the 10 individuals based on SRY sorting were mainly used for comparison with the flow sorting method (Supplementary Table 4 and 6). We have deleted the sentence “we limited our dataset collection to Nanopore or PacBio CLR data for the 10 individuals” in the Discussion section.

13. The authors discuss the suboptimal performance of SRY in the PAR is contributed by the restricted data types. I thought it was contributed by the lower density of the markers? The PAR parental marker density was very similar to that of autosomes, with stretches of runs of homozygosity, presumably to maintain enough homology for recombination. What was the marker density in the PAR? Was it below their 7 kmer / 1kb?

Response: Thank you for your question. You are correct that the suboptimal performance of SRY in the PAR is due to the lower density of markers. We apologize for any confusion caused by our statement. We have re-examined the data and found that there are no MSK in the PAR region. Considering the coherence of the context, we have removed the sentence in question.

14. The authors mentioned there are no ZW genomes available to test SRY. There is a Zebra finch trio (ZW, female, bTaeGut2) and a male sample (ZZ, male, bTaeGut1) available with HiFi of the child (bTaeGut2) and Illumina of all the genomes from the Vertebrate Genomes Project (Rhie et al., Nature, 2021). Perhaps the authors could apply SRY on this individual, and compare the W chromosome results to what has been released on https://www.genomeark.org/vgp-all/Taeniopygia_guttata.html.

Response: Thank you for providing the information about the availability of ZW genomes for testing SRY. We firstly performed sex determination on seven individuals using the chrW sequence from the reference genome. Our analysis revealed that four individuals were female while three were male. Utilizing SRY, we obtained 19,128,829 specific kmers and sorted approximately 275.8 Mb of HiFi sequences. We assembled the sorted HiFi sequences using hifiasm and verkko softwares. Comparing the two assemblers, we found that hifiasm showed slightly better results (hifiasm total length: 19.7 Mb, N50: 516.3 Kb; verkko total length: 18.1 Mb, N50: 478.3 Kb). The assembly size from hifiasm closely approximated the size of the reference genome (~20.0 Mb when exclude N) and shows good collinearity (figure below). However, our assembly results did not cover the PAR region, which is a limitation of our method.

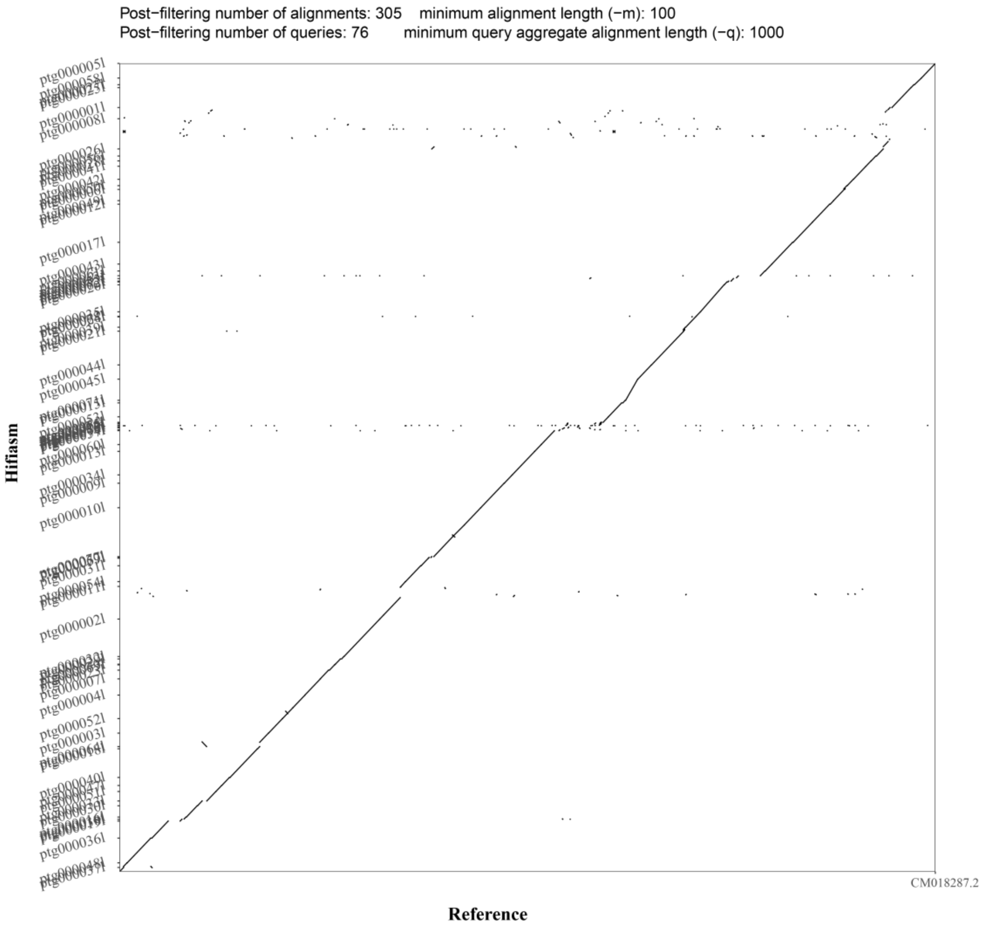
Post−filtering number of alignments: 305   minimum alignment length (−m): 100
Post−filtering number of queries: 76   minimum query aggregate alignment length (−q): 1000
Hifiasm
Reference
CM018287.2
ptg000005l
ptg000008l
ptg000017l
ptg000044l
ptg000045l
ptg000010l
ptg000002l
ptg000004l
ptg000052l
ptg000036l
