# Supplementary material for: An effective strategy for assembling the sex-limited chromosome
Source: Gigascience. 2024 Apr 16;13:giae015. doi: 10.1093/gigascience/giae015 (PMC11020242; doi:10.1093/gigascience/giae015)
Supplement: giae015_GIGA-D-23-00223_Revision_2 [file giae015_giga-d-23-00223_revision_2.pdf]

GigaScience

An effective strategy for assembling the sex-limited chromosome

--Manuscript Draft--

**Manuscript Number:**	GIGA-D-23-00223R2
**Full Title:**	An effective strategy for assembling the sex-limited chromosome
**Article Type:**	Technical Note
**Funding Information:**	National Natural Science Foundation of China (91731304) — Prof Jue Ruan<br>National Key Research and Development Program of China (2019YFA0707003) — Prof Jue Ruan<br>National Natural Science Foundation of China (31860638) — Prof Qing-You Liu
**Abstract:**	Background<br>Most currently available reference genomes lack the sequence map of sex-limited (such as Y and W) chromosomes, which results in incomplete assemblies that hinder further research on sex chromosomes. Recent advancements in long reads sequencing and population sequencing have provided the opportunity to assemble sex-limited chromosomes without the traditional complicated experimental efforts.<br>Findings<br>We introduce the first computational method, Sorting long Reads of Y or other sex-limited chromosome (SRY), which achieves improved assembly results compared to flow sorting. Specifically, SRY outperforms in the heterochromatic region and demonstrates comparable performance in other regions. Furthermore, SRY enhances the capabilities of the hybrid assembly software, resulting in improved continuity and accuracy.<br>Conclusions<br>Our method enables true complete genome assembly and facilitates downstream research of sex-limited chromosomes.
**Corresponding Author:**	xiaobo wang<br>Chinese Academy of Agricultural Sciences Agricultural Genomes Institute at Shenzhen<br>Shenzhen, CHINA
**Corresponding Author Secondary Information:**	
**Corresponding Author's Institution:**	Chinese Academy of Agricultural Sciences Agricultural Genomes Institute at Shenzhen
**Corresponding Author's Secondary Institution:**	
**First Author:**	xiaobo wang
**First Author Secondary Information:**	
**Order of Authors:**	xiaobo wang<br>Hong-Wei Lu<br>Qing-You Liu<br>A-Lun Li<br>Hong-Ling Zhou<br>Yong Zhang<br>Tian-Qi Zhu<br>Jue Ruan
**Order of Authors Secondary Information:**	

Powered by Editorial Manager® and ProduXion Manager® from Aries Systems Corporation

**Response to Reviewers:**	Reviewer #1: After reading the revised article, the questions I had previously posed were answered. I am very interested in this SRY method and believe it is also an important part of sex chromosome research. From my personal point of view, it is not easy to collect Trio data for most species except a few, but it is relatively easy to collect HIC data. It would be helpful if the authors could also compare the results of SRY HIFI with those of Hifiasm (HIC phased) to help people choose the right tool for sex chromosome assembly. However, this is not necessary, because SRY has achieved a very good result in humans. Overall, the data and results are convincing.<br>Response: Thank you for your thoughtful feedback and interest in our SRY method for sex chromosome research. We will consider incorporating Hi-C data in future versions of SRY and compare with those of Hifiasm or Verkko (HiC phased).<br><br>Reviewer #3: The authors have addressed most of my concerns. The revised manuscript reads much better than before.<br><br>Regarding my last comment and response from the authors about the W chromosome, I was hoping to see comparable coverage of the W chromosome to the reference, as a proof of principle that SRY could be applied to non-human, highly diverged genomes. The assembly looks very fragmented though. Was it only the similarity to the Z chromosome that caused the fragmentation? Are there no other factors contributing to the discontinuity of the W chromosome?<br>Response: In response to your question about the fragmentation of the W chromosome in our study, we would like to provide some clarifications. We sorted the W chromosome at a coverage of 14.0X, which is slightly lower than the reference genome's coverage of 18.3X. In highly diverged genomes, the similarity between the W and Z chromosomes primarily affects the assembly of the pseudoautosomal region (PAR).<br>One contributing factor to the fragmentation of the W chromosome assembly is the non-uniform distribution of specific markers. HiFi sequencing data that is predominantly located in regions with lower marker density is difficult to separate, leading to some degree of data loss.<br>The PacBio CLR data from the 2021 Nature article was at 47.5X for the W chromosome (assembling contig size: 20.0Mb, contig N50: 1.5Mb), while the HiFi sequencing data was less than half of that (assembling contig size: 19.7Mb, contig N50: 0.5Mb). The difference in sequencing data quantity may also have contributed to the fragmented assembly observed.<br><br>A few minor comments below to the revised version:<br>1.Please indicate which genome was compared in the legend of Supp. Table 5.<br>Response: The genome compared in the legend of Supplementary Table 5 is HG01109. We have made the modification to indicate this.<br><br>2.When using \`et al\` notations, please use the last name. Mari et al should be Serra Mari et al., Mikko et al should be Rautiainen et al. Also, Serra Mari et al is now published in Genome Biology: https://doi.org/10.1186/s13059-023-03160-z. Please update the reference.<br>Response: Thank you for the clarification. We have now made the adjustments to use the last names when using et al notations. Additionally, we have updated the reference for Serra Mari et al.<br><br>3.There are a few grammar corrections to make.<br>Response: We have made the grammar corrections in the text and highlighted them in blue.
**Additional Information:**	
**Question**	**Response**
Are you submitting this manuscript to a special series or article collection?	No
**Experimental design and statistics**	Yes

Powered by Editorial Manager® and ProduXion Manager® from Aries Systems Corporation

Full details of the experimental design and statistical methods used should be given in the Methods section, as detailed in our Minimum Standards Reporting Checklist. Information essential to interpreting the data presented should be made available in the figure legends.<br><br>Have you included all the information requested in your manuscript?	
**Resources**<br><br>A description of all resources used, including antibodies, cell lines, animals and software tools, with enough information to allow them to be uniquely identified, should be included in the Methods section. Authors are strongly encouraged to cite Research Resource Identifiers (RRIDs) for antibodies, model organisms and tools, where possible.<br><br>Have you included the information requested as detailed in our Minimum Standards Reporting Checklist?	Yes
**Availability of data and materials**<br><br>All datasets and code on which the conclusions of the paper rely must be either included in your submission or deposited in publicly available repositories (where available and ethically appropriate), referencing such data using a unique identifier in the references and in the "Availability of Data and Materials" section of your manuscript.<br><br>Have you have met the above requirement as detailed in our Minimum Standards Reporting Checklist?	Yes

Manuscript
Click here to access/download;Manuscript;assembly_strategy_for_productio

An effective strategy for assembling the sex-limited chromosome

Xiao-Bo Wang[1#], Hong-Wei Lu[1#], Qing-You Liu[2#], A-Lun Li[1], Hong-Ling Zhou[1], Yong Zhang[5], Tian-Qi Zhu[3,4*], Jue Ruan[1*]

1. Shenzhen Branch, Guangdong Laboratory for Lingnan Modern Agriculture, Genome Analysis Laboratory of the Ministry of Agriculture and Rural Affairs, Agricultural Genomics Institute at Shenzhen, Chinese Academy of Agricultural Sciences, Shenzhen, Guangdong 518120, China
2. Guangdong Provincial Key Laboratory of Animal Molecular Design and Precise Breeding, School of Life Science and Engineering, Foshan University, Foshan 528225, China
3. National Center for Mathematics and Interdisciplinary Sciences, Academy of Mathematics and Systems Science, Chinese Academy of Sciences, Beijing 100190, China
4. Key Laboratory of Random Complex Structures and Data Science, Academy of Mathematics and Systems Science, Chinese Academy of Sciences, Beijing 100190, China
5. Key Laboratory of Zoological Systematics and Evolution & State Key Laboratory of Integrated Management of Pest Insects and Rodents, Institute of Zoology, Chinese Academy of Sciences, Beijing 100101, China
[#]These authors contributed equally to this work.
[*]Corresponding author: E-mail: ruanjue@caas.cn; zhutq@amss.ac.cn

Xiaobo Wang: 0000-0001-6754-7404
Qingyou Liu: 0000-0003-3265-540X
Hongling Zhou: 0000-0002-7295-8176
Jue Ruan: 0000-0003-3713-3192

Abstract

Background

Most currently available reference genomes lack the sequence map of sex-limited (such as Y and W) chromosomes, which results in incomplete assemblies that hinder further research on sex chromosomes. Recent advancements in long reads sequencing and population sequencing have provided the opportunity to assemble sex-limited chromosomes without the traditional complicated experimental efforts.

Findings

We introduce the first computational method, Sorting long Reads of Y or other sex-limited chromosome (SRY), which achieves improved assembly results compared to flow sorting. Specifically, SRY outperforms in the heterochromatic region and demonstrates comparable performance in other regions. Furthermore, SRY enhances the capabilities of the hybrid assembly software, resulting in improved continuity and accuracy.

Conclusions

Our method enables true complete genome assembly and facilitates downstream research of sex-limited chromosomes.

Introduction

Traditionally, genomes of homogametic individuals (XX females or ZZ males) have been preferred for genome sequencing projects, because the haploid nature of both sex chromosomes (X and Y, or Z and W) in heterogametic species reduces sequencing depth which can lead to decreased assembly contiguity and length[1]. Although XY or ZW chromosomes have significantly diverged from their ancestral autosomes[2, 3], they still exhibit homology, which can pose challenges for genome assembly. Homologous regions, such as the pseudoautosomal regions (PAR), can result in fragmented contigs similar to large repeats. Plenty of repetitive sequences in sex-limited (Y or W) chromosome further increase the assembly difficulties.

Currently, there are two main experimental approaches aimed at solving the problem. The first approach, known as the BAC-based method, has been applied in deciphering the Y chromosomes of several mammals including human[3], chimpanzee[4], rhesus macaque[5], and mouse[6]. However, it is time-consuming, labor-intensive and expensive. The second approach is chromosome flow-sorting, which relies on chromosome size and GC content (**Fig. 1a**) and offers high automation and throughput[1]. However, it requires cells to be in metaphase, where chromosomes are in a condensed state that can be easily physically separated[7]. Additionally, it may mistakenly sort other chromosomes or debris with similar sizes or GC contents to the sex-specific chromosome, which can introduce bias during the amplification stage[1, 8].

Takashi *et al*. used the F_1 population data of the persimmon to identify male-specific markers[9], and utilized these markers to partition and assemble short reads. However, they did not provide software for the algorithm or consider the effect of population heterogeneity on the identification of male-specific markers. YGS[10] compares the male assembly results with length k subsequences (*k*-mers) of female short reads to obtain Y contigs. The Sex-detector[11] uses pedigree data to identify sex-specific genes in RNA-seq assemblies. However, all three methods lack the ability to sort long reads to reduce the assembling difficulty.

Thanks to the longer read lengths and higher sequencing accuracy, long reads have a higher potential to be identified to their original chromosome by pure computing methods. Recently, trio

binning was developed to sort long reads *in silico*[12] using specific markers (**Fig. 1b**). It compares *k*-mers of short reads from parental genomes and identifies *k*-mers that are unique to each parent. These *k*-mers are then used to separate long reads of the offspring and conducts haplotype assemblies, separately. Theoretically, Y- (or W-) specific markers[13] can be selected and used for sorting long reads from the sex-limited chromosome. Compared to whole genome shotgun (WGS) assembly, trio binning assembly covers more genomic regions of the Y chromosome with better contiguity (**Supplementary Table 4**). It indicates that the computational method is promising, though trio binning cannot efficiently address the problem of assembling sex-limited chromosome based on its scheme to select specific markers. The Telomere-to-Telomere (T2T) consortium has used various third-generation sequencing technologies to complete the assembly of the Y chromosome[13], but the process requires substantial manual adjustments. It is worth noting that recent marker-based graph phasing algorithms in long-reads, such as hifiasm[14] trio and verkko[15] trio mode, have emerged as alternative approaches to enhance the accuracy and efficiency of phasing. Specially, verkko is a successor of the manual efforts taken in T2T-Y. Additionally, there are alternative approaches for phasing genomes in plants. For instance, Serra Mari et al.[16] proposed a polyploid potato phasing method that utilizes many siblings of the child for genotyping, and achieved significant results.

To further improve the quality of sex-specific chromosome assembly and reduce the need for manual curation, we try to find new solutions from population datasets. In whole genome sequencing, the sequencing depth between sex chromosomes and autosomes is different. For example, in a XY male, the sequencing depth of the X or Y chromosome is half that of autosomes. Therefore, X/Y-specific markers can be separated by different sequencing depth. Moreover, the X-specific markers also exist in females, so they can be removed from X/Y-specific markers to obtain Y-specific markers. By incorporating population data into our analysis, we anticipate a reduction in the impact of sequencing coverage and allelic genotype variations when identifying Y chromosome-specific markers in a comparative analysis involving only two individuals of different genders.

Results

Overview of SRY

To reach the goal of sorting long reads of sex-limited chromosome, we developed an *in silico* sorting method called SRY (**Fig. 1c**). The process of SRY involves identifying sex-specific markers by comparing male and female populations, and subsequently sorting long sequences according to the specific markers. SRY firstly selects *k*-mers with half of the sequencing depth in male populations. Subsequently, SRY filters out X-linked *k*-mers and *k*-mers originating from heterozygous sites found in female populations, thereby enabling the identification of male-specific *k*-mers (MSK). Due to the impact of population structure and sequencing errors, the operation of SRY is in fact a sampling process, which unavoidably involves *k*-mers from X chromosome and autosomes. Therefore, SRY calculates the MSK density of long reads and excludes those with lower marker density. These separated long reads are subsequently delivered to the assemblers to perform genome assembly. Moreover, MSK can be used to select Y chromosome contigs from a whole genome assembly from a male individual[17-19].

Evaluating SRY with theoretical models and simulated data

There are two primary factors contributing to false positives in the identification of specific *k*-mers by SRY: coverage and population heterogeneity. Accordingly, we constructed theoretical models of the false positive and true positive to assess MSK identified by SRY (see the Methods section for details). In addition, we used the mason_simulator[20] software to simulate the data under different heterogeneity and different number of individuals (5X for each individual) for the evaluation of SRY. Consistent with the theoretical results, the results based on simulated data show that an increase in population heterogeneity decreases the F1-score of SRY, while an increase in the number of individuals augments the F1-score of SRY (**Fig. 2a**). Notably, when the number of individuals in both male and female populations is less than 7, the increase in the number of individuals has a significant effect on improving the F1-score of SRY (**Fig. 2a**). However, once the number exceeds 7, the increase in the number of individuals has little effect (**Fig. 2a**).

Furthermore, we provide a theoretical model for the process of sorting long reads of Y chromosome (see Methods section for details). The central issue addressed by this model is determining the probability that at least M markers are retained in the corresponding error-prone long reads within a genomic region consisting of N specific markers. In addition to theoretical values, we used badread[21] software to simulate the human T2T genome with 50X each of HiFi, Nanopore and PacBio CLR reads, and set a range of MSK precision level to assess the performance of SRY on sorting long reads. Remarkably, even when the precision of MSK decreases to 70%, we found that the F1-score of SRY remains above 90% (**Fig. 2b**). This can be attributed to the fact that the genome size of autosomes and X chromosome is approximately 3Gb, resulting in a low density of non-specific k-mers (non-MSK) derived from these chromosomes (1 k-mer/kb). The filter criterion of SRY is ~7 *k*-mers per kilobase, making it easy to exclude these non-Y chromosome sequences.

Comparison with the experimental method on real data

We collected datasets including short and long reads of a Chinese individual HX1[22,23], and re-sequencing short reads of a Han Chinese population[24] to identify MSK (**Supplementary Table 1 and Table 2**). SRY obtained about 10 million MSK as well as sorted 3.7G (~46X) PacBio CLR and (~13X) ONT long reads of Y chromosome (**Supplementary Table 3**). We further collected Nanopore long reads (~2.3G, number of reads is 305,284) of an African human Y (HG02982) separated by flow sorting[8] and used minimap2[25] to align the sorted long reads from the two methods to the human T2T genome, separately. The results show that 94.0% of the sorted reads from SRY are mapped on T2T-Y chromosome, which is significantly higher than that of flow sorting (**Fig. 3a**). The human Y chromosome consists of several distinct regions (ampliconic, X-degenerate, X-transposed, pseudoautosomal, heterochromatic, others)[3]. We compared the performance of the two methods in these regions and found that SRY demonstrates comparable performance to flow sorting in the ampliconic, X-degenerate, X-transposed and others (**Fig. 3b**). However, SRY outperforms in terms of coverage and depth specifically in the heterochromatic region. (**Fig. 3b, c and Supplementary Table 4**). Notably, the event of X-Y recombination is frequent on pseudoautosomal region (PAR, PAR1:1-2.8Mb, PAR2:56.9-57.2Mb). SRY aims to obtain Y-specific markers, hence the low coverage and shorter assembled result on PAR of SRY is expected (**Fig. 3b and Supplementary Table 4**).

We further compared the resulting assemblies between the experimental and computational

methods. We collected a trio dataset consisting of the parental genomes HG01107 and HG01108, as well as the offspring genome HG01109, in order to perform trio binning and compare the resulting assemblies of HG01109. SRY can achieve sort reads first and then assemble, or directly sort the assembled contigs based on MSK. The fast assembler wtdbg2[26] was used to assemble those sorted reads and flow sorting reads, and perform genome assemblies for trio binning and WGS (**Supplementary Table 3**). The total contig alignment length on T2T-Y chromosome from SRY is ~5.7Mb and ~9.6Mb longer than those from the sorted contigs of trio binning and WGS respectively. Moreover, the alignment lengths and the contiguity (NA50) on each discrete region of Y chromosome from SRY are all longer than the other two methods (**Supplementary Table 5**), indicating that it is better to sort the reads first and then assemble them. Additionally, the SRY assembly exhibits lower contamination from other chromosomes compared to flow sorting (**Supplementary Table 4**). Similarly to the result obtained from read sorting, the assembly result from SRY performed better in heterochromatic regions compared to flow sorting. However, in the PAR, the assembly result from SRY was inferior to those from flow sorting.

Towards complete genome assembly of Y chromosome

Rautiainen et al[15] developed an assembly software called Verkko, designed for HiFi and ultra-long Nanopore data, in order to achieve better automation of T2T-level chromosome assembly. Verkko demonstrated good result in the assembly of HG002. We sorted the Y chromosome data from HG002 and used Verkko for assembly. The results showed that, compared to Verkko with trio[15], the assembly of the sorted data (Verkko with SRY) reduced the number of contigs from 23 to 9 and corrected one assembly error (**Fig. 4**). Additionally, due to the high similarity of the X and Y chromosome PAR regions, Verkko trio's assembly result did not phasing this region well, resulting in two approximately 1Mb contigs aligning to the same region of the Y chromosome (**Fig. 4a**). Verkko SRY not only assembled this region completely, but also with higher accuracy (**Fig. 4b**). This indicates that SRY can further improve the performance of the assembly software with new sequencing technology.

We further selected the genome of the yellow catfish[27], which contains young Y chromosomes, for simulated evaluation. The similarity between its Y chromosome and X chromosome exceeds 99%. To facilitate evaluation, we removed the gaps in the genome. We simulated 100X HiFi and ultra-long Nanopore data (50X for the Y chromosome) respectively. As a result, we sorted ~45X HiFi data and ~37X ultra-long Nanopore data of the Y chromosome. The assembly size of the results using verkko software was 42.7Mb (98.8% of the Y chromosome), consisting of only 2 contigs. According to the quast results, both contigs aligned almost perfectly to the reference Y chromosome. This indicates that our software can achieve relatively good results in assembling young Y chromosomes as well.

Discussion

The deciphering of sex chromosomes is crucial for studying reproductive biology, sex determination, and other key molecular processes that contribute to the evolutionary trajectory of species. To enable more complete assembly of sex chromosome sequences, we have developed the SRY method, a software tool that efficiently sorts third-generation long reads of sex-limited chromosomes based on male-specific k-mer (MSK) markers.

The performance of SRY can be influenced by the number of male and female individuals,

whereas flow sorting only requires one male individual of interest and is not affected. However, SRY outperformed flow sorting in terms of read sorting accuracy and demonstrated comparable or better performance in most regions, except for the pseudoautosomal region (PAR) where SRY had lower coverage and assembly results. The effectiveness of SRY is also influenced by the lengths of the reads and the quality of the base pairs. Notably, the incorporation of HiFi and ultra-long Nanopore data significantly improved the assembly quality of SRY in the PAR region. The capability of SRY to effectively sort long reads of sex-limited chromosomes (regardless of their age), highlight its potential as a valuable alternative to experimental methods for studying sex-specific genomic regions.

With the further reduction in sequencing costs, there will be a greater availability of population-level second and third-generation sequencing data. After identifying MSK using second-generation data, SRY can be utilized for sorting and assembling third-generation long reads from all individuals within the population. The application and comparison of sex chromosomes within the population will contribute to our understanding of complex biological processes and genetic variations.

Methods

SRY process

Firstly, SRY used kmer_count program to acquire *k*-mer (k=21) [12] sets from short reads of targeted male species and populations. Next, the program filterx [28] was used to identify specific *k*-mers associated with the male population. We labeled the *k*-mer files of all male individuals as "group1" and all female individuals as "group2". By comparing these groups and identifying *k*-mers as specific *k*-mers that are present in at least 2/3 of the individuals from group1. Then, SRY selects long reads of targeted species that have male specific *k*-mers (MSK). Finally, SRY filters those long reads with lower MSK densities than average value of whole Y chromosome.

False positive for MSK

False positive is introduced if a subsequence of length k (k-mer) originated from autosomes or X chromosome is incorrectly identified as a MSK, with two possible sources: genomic coverage and population heterogeneity.

In the model, n males and n females are sequenced with the sequencing depth d. The length of a read is l and the sequencing error per site is r. C_n^i represents the number of ways to choose i elements from a set of n elements, also known as the binomial coefficient. In an individual, only the frequency of appearance of a k-mer that is more than once can we consider it to be present, and this event occurs with probability p_o. A k-mer is identified as an MSK if it is present in at least m males but not in any of the females. Particularly, we use m = 2/3n as the critical value by simulation study.

Let X be the frequency of appearance of a k-mer is present in an individual, then it follows a Possion distribution with rate $\lambda = d(1-r)^k(l-k+1)/l$. It is easier to calculate $q_o = 1 - p_o$, which is the probability that a k-mer is absent, and thus

$q_o = P(X = 0) + P(X = 1) = e^{-\lambda} + \lambda e^{-\lambda} = (1 + \lambda)e^{-\lambda}$

We then can calculate the false positive rate caused by genomic coverage (f_1) by

$$f_1 = q_o^n \sum_{i=\lceil \frac{2}{3}n \rceil}^{n} C_n^i p_o^i q_o^{n-i} \quad (0.1)$$

If we set r = 0.01, l = 150, d = 5, k = 21 and n = 5, then f_1 is roughly 4.4×10^{-5}. If the sample size n increases to 10, then f_1 decreases to 1.9×10^{-9}, indicating that the false positive introduced by genomic coverage can be ignored if the sample size n is not too small.

For simplicity, we only consider heterogeneity in autosomes, and ignore heterogeneity in X chromosome. Assume one heterozygous site leads to two kinds of k-mers (k-mer-1 and k-mer-2), and the heterozygous proportions for two k-mers are p_{h1} and p_{h2} (with $p_{h1} + p_{h2} = 1$). If k-mer-j (j=1,2) from autosomes is observed in many males while it is not observed in any females, then k-mer-j is mistakenly identified as an MSK. As the discussion for the case of genomic coverage, the frequency of appearance of k-mer-j, X_j, follows Poisson distributions with parameters $\lambda_j = p_{hj}\, \lambda = p_{hj}\, d\, (1- r)^k(l - k + 1)/l$. As before, if a k-mer appears less than twice in an individual, we consider the k-mer is absent, and the probability of this event q_{oj} can be calculate as follows
$q_{oj} = P(X_j = 0) + P(X_j = 1) = e^{-\lambda j} + \lambda_j\, e^{-\lambda j} = (1+\lambda_j)\, e^{-\lambda j}$.
Given the heteropoietic rate h = 0.001, the probability that a k-mer with length 21 contains more than one heterozygous site is only 2.1×10^{-4}, which can be neglected. We also ignore the probability that k-mer-1 is identified as k-mer-2 mistakenly with edit distance 1 due to sequencing error, as such events occur with probability 0.99^20*r/3 = 0.3%. Then the false positive rate due to population heterogeneity f_2 can be calculated as follows:

$$f_2 = q_{o1}^n \sum_{\lceil i=\frac{2}{3}n \rceil}^{n} C_n^i p_{o1}^i q_{o1}^{n-i} + q_{o2}^n \sum_{\lceil i=\frac{2}{3}n \rceil}^{n} C_n^i p_{o2}^i q_{o2}^{n-i}. \quad (0.2)$$

The total false positive rate f is a weighted average of the false positive rate from the two sources, that is

$$f = (1 - kh)\, f_1 + khf_2 \quad (1.3)$$

Note that f_2 is actually a function of p_{hj}, which is an unknown parameter in the model. We further used the biallelic SNV datasets from 1000 genome project [29] to estimate the empirical distribution of p_{h1}. We use a discrete distribution ranging from 0 to 0.5 to characterize the distribution of p_{h1}, which takes value of 0.05, 0.15, 0.25, 0.35 and 0.45 with probability 92.77%, 2.59%, 1.80%, 1.48% and 1.36%, respectively. Combining the uncertainty of heterozygotic rate, the false positive rate is:

$$f = (1 - kh)\, f_1 + kh \sum_x f_2(x) P(p_{h1} = x). \quad (0.3)$$

True positive rate (TPR) of identifying MSK

As discussed before, the probability of a k-mer present in more than two-thirds of male individuals is $\sum_{i=\frac{2}{3}n}^{n} C_n^i p_{oj}^i q_{oj}^{n-i}$. As the probability that a k-mer from autosomes is identified as a MSK due to sequencing error is too small, the probability that an MSK is present in none of the females is roughly 1. Then the TPR of identifying MSK is the product of the probability of the two events, that is

$$\text{TPR} = \sum_{i=\frac{2}{3}n}^{n} C_n^i p_{oj}^i q_{oj}^{n-i}$$

The probability of sorting long reads

For third-generation long reads, the sequencing errors are higher (PacBio CLR or Nanopore) and their lengths vary a lot. Assume a long read contains *N* specific markers, with *N* to be the function of the read length and the distribution of MSK, and the probability that a *k*-mer is correctly sequenced is $p = (1\text{-}r_3)^k$ (r_3 is the sequencing error of long reads). Then the probability that at least *M* MSKs are identified is

$$\sum_{i=M}^{N} C_N^i p^i (1-p)^{N-i}$$

In SRY software, the average number of MSKs (about 7/kb) across the Y chromosome is taken as the value of *M* to sort Y-chromosome long reads.

Assessment

In order to evaluate the MSK identification process of SRY, we firstly used kmer_count to obtain the *k*-mer of all chromosomes of the human T2T genome, and used filterx [28] to identify the specific *k*-mer of T2T-Y chromosome, which served as the standard for subsequent evaluations. Then, we used the mason_simulator (v2.0.9) program in the mason[20] package with the parameter (--illumina-prob-mismatch 0.009 --illumina-prob-insert 0.0005 --illumina-prob-deletion 0.0005 --illumina-read-length 150) to generate short-read data for male and female populations using the human T2T genome with or without T2T-Y chromosome as a reference, respectively. We used different seed values for all individuals to avoid the result that the simulated data for all individuals were same. Finally, SRY used these population data to identify MSKs, which were evaluated by comparison with T2T-Y chromosome-specific *k*-mers.

For the theoretical value of the precision of SRY on identifying MSK, we used the following formula:

YSK*TPR/(YSK*TPR + AXK*FPR)

Where YSK represents the specific *k*-mer number of T2T-Y chromosome, AXK represents the *k*-mer number of the human T2T autosomes and X chromosome, and TPR represents the true positive rate and FPR represents the false positive rate of identifying MSK, respectively.

We further simulated 50X Nanopore, PacBio CLR and HiFi reads (25X for Y chromosome) based on the human T2T genomes using badread[21] package (v0.1.3) with the following commands, respectively:

badread simulate --reference human_autoX.fa --quantity 50X --error_model nanopore --start_adapter 0,0 --end_adapter 0,0 --junk_reads 0 --random_reads 0 --chimeras 0 (simulated Nanopore reads of autosomes and X chromosome)

badread simulate --reference human_Y.fa --quantity 25X --error_model nanopore --start_adapter 0,0 --end_adapter 0,0 --junk_reads 0 --random_reads 0 --chimeras 0 (simulated Nanopore reads of Y chromosome)

badread simulate --reference human_autoX.fa --quantity 50X --error_model pacbio --identity 85,95,3 --length 7500,7500 --start_adapter 0,0 --end_adapter 0,0 --junk_reads 0 --random_reads

0 --chimeras 0 (simulated PacBio CLR reads of autosomes and X chromosome)
badread simulate --reference human_Y.fa --quantity 25X --error_model pacbio --identity 85,95,3 --length 7500,7500 --start_adapter 0,0 --end_adapter 0,0 --junk_reads 0 --random_reads 0 --chimeras 0 (simulated PacBio CLR reads of Y chromosome)
badread simulate --reference human_autoX.fa --quantity 50x --error_model pacbio --qscore_model pacbio --identity 99,100,3 --length 12000,12000 --start_adapter 0,0 --end_adapter 0,0 --junk_reads 0 --random_reads 0 --chimeras 0 (simulated PacBio HiFi reads of autosomes and X chromosome)
badread simulate --reference human_Y.fa --quantity 25x --error_model pacbio --qscore_model pacbio --identity 99,100,3 --length 12000,12000 --start_adapter 0,0 --end_adapter 0,0 --junk_reads 0 --random_reads 0 --chimeras 0 (simulated PacBio HiFi reads of Y chromosome)
SRY was assessed for its ability to sort these simulated long reads by considering different TPR values of MSK.
Even the precision of MSK decreases to 70% (including ~7,000,000 MSKs and ~3,000,000 non-MSKs), the density of these non-MSKs on autosomes and X chromosomes was only 1 per kb, which was significantly lower than the threshold set by SRY (7/kb). Therefore, we took the theoretical precision of SRY on sorting long reads as 1. For simplicity, the process of calculating the TPR of SRY on sorting long reads ignored the length distribution of the reads and used the window with 10kb length to calculate the specific *k*-mer distribution of the T2T-Y chromosome.

Y chromosome assembly, identification and evaluation

We collected ~60X ultra-long ONT data and ~35X HiFi data[15], and used SRY for the long-read sorting of the Y chromosome. Due to the abundance of repetitive sequences in the heterochromatic regions of the Y chromosome, the number of available Y-specific markers is limited. Therefore, we used two lengths of *k*-mers (*k*=21 and *k*=51) for the sorting of HiFi reads. Verkko (v1.0)[15] was used to assemble the selected data with parameters (-d Asm --hifi hifi.sorted.fq.gz --nano ul-ont.sorted.fq.gz --threads 128). We compared the assembly results of Verkko in trio mode (collected from ref.[15]) and SRY mode to T2T-CHM13 using Quast (v5.0.2) [30]. The file with the suffix name "coords.filtered" was used by DotPlotly [31] (parameters: -slt -m 100 -q 100) to generate the alignment plot.

We collected the dataset of a trio family including short reads from father (HG01107, ~113X) and mother (HG01108, ~79X) and Nanopore reads from child (HG01109, ~72X) [32]. SRY separated 1.3G (~25X) long reads of HG01109 using MSK markers identified from HX1. Then, we used wtdbg2.5[26] with parameters "-L 0 -p 0 -k 21 -s 0.25 -S 2 --rescue-low-cov-edges" to assembly those long reads. The remaining long reads were assembled by wtdbg2.5 with parameters "-x ont -g 3g" and polished with the program wtpoa-cns in wtdbg2.5. All of the assembled contigs were further polished with wtpoa-cns using short reads. Sorting of long reads and genome assemblies for other nine individuals were performed the same way. Trio binning phased HG01109 long reads using with command "canu -stopAfter=haplotype genomeSize=3g -haplotypeMale HG01107.fastq.gz -haplotypeFemale HG01108.fastq.gz -nanopore-raw HG01109.fasta.tar.bz2". Wtdbg2 with the parameters (-g 3.1G -x ont) was applied to assembly phasing reads from trio binning and perform whole genome assembly for WGS. SRY was then used to partition candidate contigs of Y chromosome for trio binning and WGS. We utilized quast (v5.0.2) [30] with default parameters to evaluate the assembled genome quality.

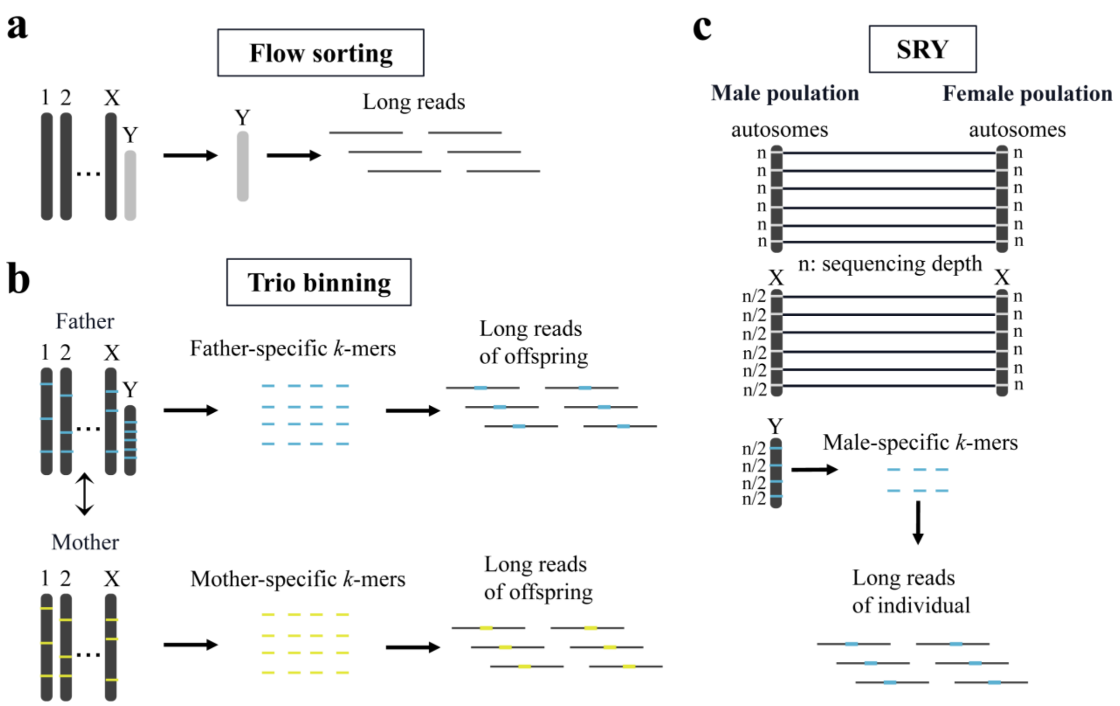

Figure 1: Overview of three methods for sorting long reads. (a) Flow-sorting is an experimental method for separating Y chromosome. **(b)** Trio binning compares *k*-mers from short reads of parent genomes and identifies father-specific and mother-specific *k*-mers, respectively. These specific *k*-mers are used to bite long reads for each parent. **(c)** SRY rules out *k*-mers presenting in both male and female populations and retains *k*-mers only occurred in male population with half sequencing depth. SRY utilizes these male-specific *k*-mers to separate long reads of Y chromosome.

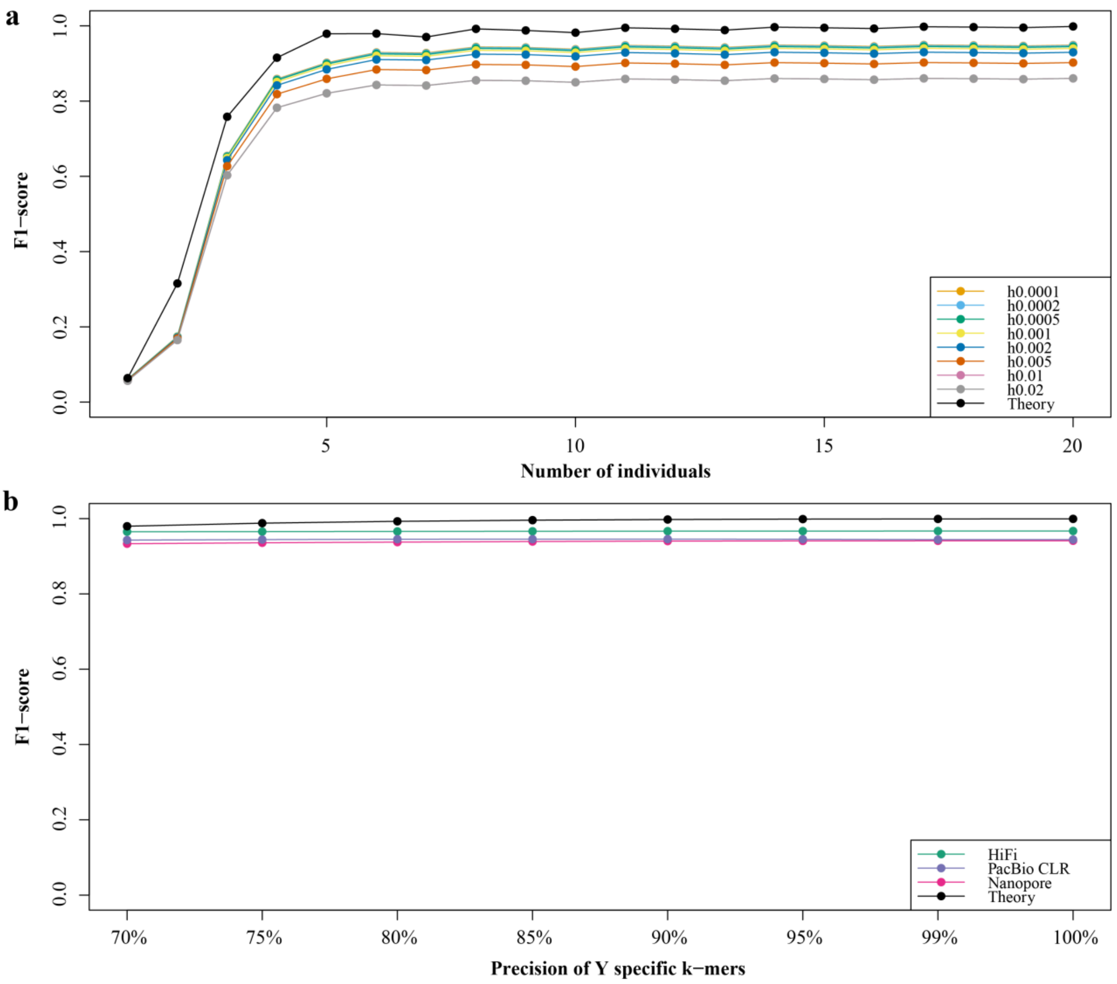

Figure 2: Theoretical model and performance of SRY on simulated data. (a) The F1-score of SRY on identifying male-specific *k*-mers with different individual number (per sex) and population heterogeneity, respectively. For simplicity, we did not consider the similarity distribution between the Y chromosome and other chromosomes in the calculation of the theoretical value. **(b)** The F1-score of SRY on sorting HiFi, PacBio CLR and Nanopore long reads. The combination of length distribution and specific *k*-mers distribution can cause TPR differences between theoretical and simulated data as well as TPR differences within simulated data.

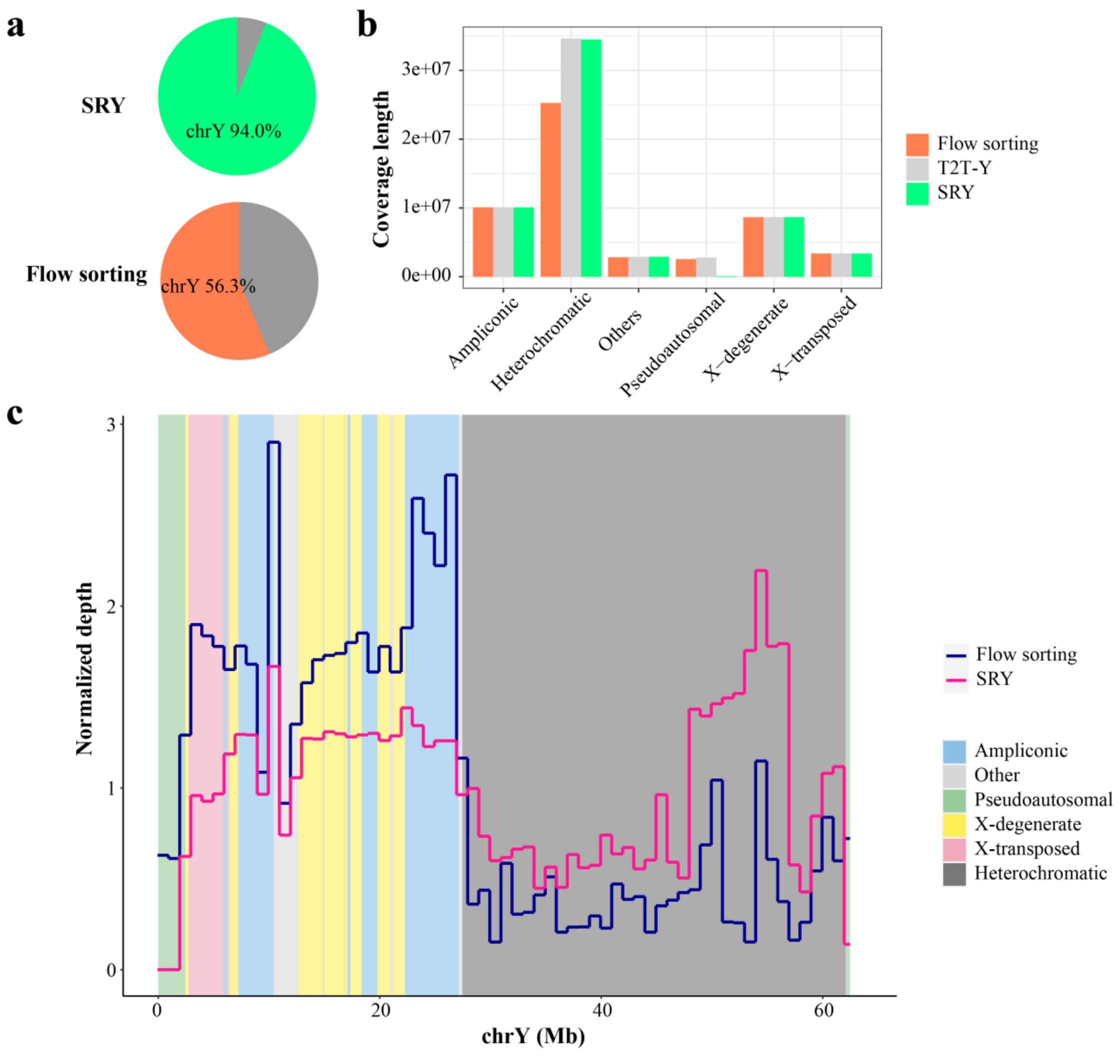

Figure 3: Comparison of sorting results between SRY and flow sorting. (a) Alignment distribution of sorted reads for SRY and flow sorting. The alignment on autosomes and X chromosome is colored in gray. **(b)** The coverage of sorting reads by the two methods on discrete regions of Y chromosome. SRY aims to separate male-specific long reads, so the coverage is lower on pseudoautosomal region where recombination events occur frequently between X and Y chromosome. **(c)** The normalized depth of long reads separated by the two methods. The colored rectangles represent discrete regions on the T2T-Y chromosome.

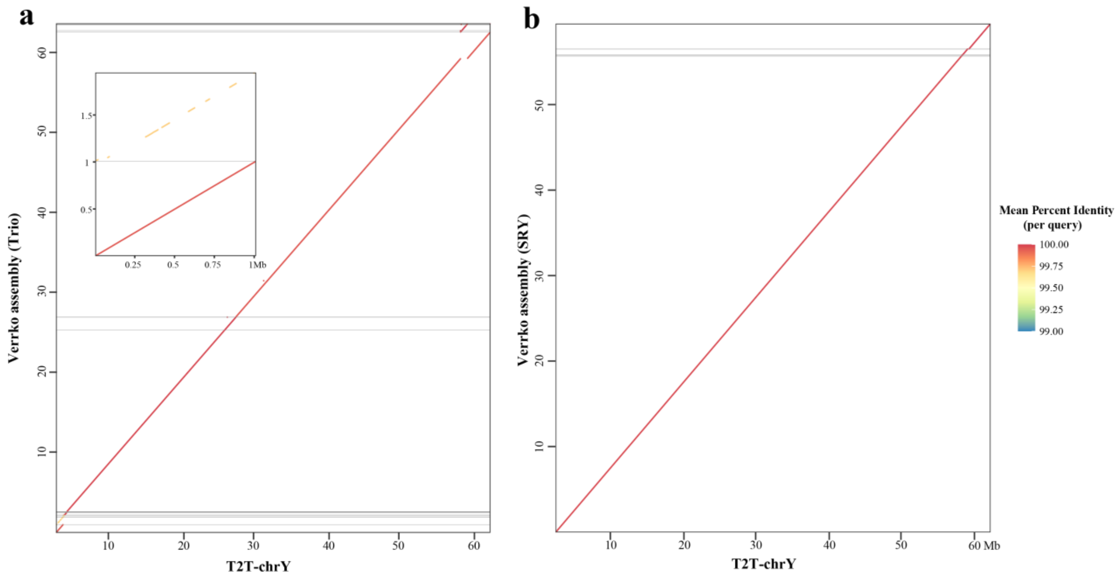

Figure 4: Verkko assembly using Trio or SRY. The x axis represents the complete Y chromosome assembled by T2T consortium, and y axes represent assemblies by verkko with Trio (**a**) and SRY (**b**), respectively. Inset in (**a**) represents an enlarged view of the first 1 Mb region of the T2T-chrY. The diagonal colored with identity shows the alignment result. (Note: T2T-Y palindrome P5 (18483410-19235627) is expected to be inverted, and this error was not resolved by the assembly using verkko SRY mode.)

Availability of supporting source code and requirements

Project name: SRY: Sorting long Reads of Y or other sex-limited chromosome
Project homepage: https://github.com/caaswxb/SRY
Operating system(s): Linux or Unix
Programming language: shell and perl
Other requirements: kmc, samtools, seqtk and parallel
License: MIT License
RRID: SCR_025036
Biotools ID: SRY

Data availability

We downloaded all Nanopore, PacBio and Illumina datasets of HX1 via NCBI, project number PRJNA301527[22,23]. The SRA numbers of Han Chinese population are listed in supplementary table 1 and 2. The trio family (HG01107, HG01108 and HG01109), HG005, HG006, HG01243, HG02055, HG03098 and HG03492 reads are available at Amason S3[33]. Short reads as well as PacBio and/or Nanopore long reads of HG002 and HG003 are available at The National Center for Biotechnology Information (NCBI)[34]. We also downloaded NCBI accession: ERR3241824 for HG01107 and NCBI accession: ERR3241825 for HG01108 to improve the performance of trio binning. All assembly results have been submitted to figshare[35].
An archival copy of the code and supporting data is also available via the *GigaScience* database GigaDB [36].

Additional Files

Supplementary Table 1. Summary of datasets for 46 males of Han Chinese population.
Supplementary Table 2. Summary of datasets for 44 females of Han Chinese population.
Supplementary Table 3. Statistics of PacBio and Nanopore reads identified by SRY from long-read sequencing datasets. P represents PacBio CLR reads, and N represents Nanopore reads. The average error rate is calculated based on the alignment results of the third-generation data on the human T2T genome.
Supplementary Table 4. Comparison of the assembled genomes for SRY and flow sorting. Human sample numbers are in parentheses, and the sample numbers and metrics of the other nine individuals are detailed in supplementary table 6.
Supplementary Table 5. Contig sorting for the HG01109 assembly results of Trio binning and WGS using SRY.
Supplementary Table 6. Statistics of nine human assemblies.

Abbreviations

SRY: Sorting long Reads of Y or other sex-limited chromosome; PAR: pseudoautosomal region; BAC: Bacterial artificial chromosome; WGS: whole genome shotgun; T2T: Telomere-to-Telomere; MSK: male-specific *k*-mers; TPR: true positive rate; NCBI: The National Center for Biotechnology Information.

Acknowledgments

This work was supported by National Natural Science Foundation of China (Grant No. 91731304 to J.R.), National Key Research and Development Program of China (Grant No. 2019YFA0707003 to J.R.) and National Natural Science Foundation of China (Grant No. 31860638 to Q. L.). We thank S. Wu from CAAS for his suggestions on genome assembly. We thank High-performance Computing Center of Agricultural Genomics Institute at Shenzhen, China Academy of Agricultural Sciences.

Author contributions

J.R. and Q. L. designed the project, and J.R. managed the project. X.W., J.R. and A.L. developed the SRY method. T.Z. constructed the theory model. X.W. and H.L. collected genomic data, performed analysis and wrote the paper. J.R., H.Z. and Y.Z. revised the manuscript.

Competing interests

The authors declare no competing interest.

Reference

1. Tomaszkiewicz M, Medvedev P, Makova KD. Y and W Chromosome Assemblies: Approaches and Discoveries. *Trends Genet.* 2017;33(4):266-282.
2. Bellott DW, Skaletsky H, Cho TJ, et al. Avian W and mammalian Y chromosomes convergently retained dosage-sensitive regulators. *Nat Genet.* 2017;49(3):387-394.
3. Skaletsky H, Kuroda-Kawaguchi T, Minx PJ, et al. The male-specific region of the human Y chromosome is a mosaic of discrete sequence classes. *Nature.* 2003;423(6942):825-837.
4. Hughes JF, Skaletsky H, Pyntikova T, et al. Chimpanzee and human Y chromosomes are remarkably divergent in structure and gene content. *Nature.* 2010;463(7280):536-539.
5. Hughes JF, Skaletsky H, Brown LG, et al. Strict evolutionary conservation followed rapid gene loss on human and rhesus Y chromosomes. *Nature.* 2012;483(7387):82-86.
6. Soh YQS, Alföldi J, Pyntikova T, et al. Sequencing the Mouse Y Chromosome Reveals Convergent Gene Acquisition and Amplification on Both Sex Chromosomes. *Cell.* 2014;159(4):800-813.
7. Doležel J, Vrána J, Šafář J, et al. Chromosomes in the flow to simplify genome analysis. *Funct Integr Genomics.* 2012;12:397-416.
8. Kuderna LFK, Lizano E, Julià E, et al. Selective single molecule sequencing and assembly of a human Y chromosome of African origin. *Nat Commun.* 2019;10(1):4.
9. Akagi T, Henry IM, Tao R, et al. A Y-chromosome–encoded small RNA acts as a sex determinant in persimmons. *Science.* 2014;346(6209):646-650.
10. Carvalho AB, Clark AG. Efficient identification of Y chromosome sequences in the human andDrosophilagenomes. *Genome Res.* 2013;23(11):1894-1907.
11. Muyle A, Käfer J, Zemp N, et al. SEX-DETector: a probabilistic approach to study sex chromosomes in non-model organisms. *Genome Bio Evol.* 2016;8(8):2530-2543.
12. Koren S, Rhie A, Walenz BP, et al. De novo assembly of haplotype-resolved genomes with trio binning. *Nat Biotechnol.* 2018;36(12):1174-1182.
13. Rhie A, Nurk S, Cechova M, et al. The complete sequence of a human Y chromosome. *Nature*. 2023;621(7978):344-354.
14. Cheng H, Concepcion G T, Feng X, et al. Haplotype-resolved de novo assembly using phased

assembly graphs with hifiasm. *Nat Methods*. 2021;18(2): 170-175.
15. Rautiainen M, Nurk S, Walenz BP, et al. Telomere-to-telomere assembly of diploid chromosomes with Verkko. *Nat Biotechnol*. 2023;41:1474-1482.
16. Serra Mari R, Schrinner S, Finkers R, et al. Haplotype-resolved assembly of a tetraploid potato genome using long reads and low-depth offspring data. *Genome Biol*. 2024;25(1):26.
17. Rangavittal S, Stopa N, Tomaszkiewicz M, et al. DiscoverY: a classifier for identifying Y chromosome sequences in male assemblies. *BMC genomics*. 2019;20(1):1-11.
18. Rangavittal S, Harris RS, Cechova M, et al. RecoverY: k-mer-based read classification for Y-chromosome-specific sequencing and assembly. *Bioinformatics*. 2018;34(7):1125-1131.
19. Hall AB, Qi Y, Timoshevskiy V, et al. Six novel Y chromosome genes in Anopheles mosquitoes discovered by independently sequencing males and females. *BMC genomics*. 2013;14(1):1-13.
20. Holtgrewe M. Mason–a read simulator for second generation sequencing data. *Technical Report FU Berlin*, 2010.
21. Wick RR. Badread: simulation of error-prone long reads. *Journal of Open Source Software*. 2019;4(36):1316.
22. Shi L, Guo Y, Dong C, et al. Long-read sequencing and de novo assembly of a Chinese genome. *Nat Commun*. 2016;7(1):12065.
23. Liu Q, Fang L, Yu G, et al. Detection of DNA base modifications by deep recurrent neural network on Oxford Nanopore sequencing data. *Nat. Commun*. 2019;10(1):2449.
24. Lan T, Lin H, Zhu W, et al. Deep whole-genome sequencing of 90 Han Chinese genomes. *GigaScience*. 2017;6(9): gix067.
25. Li H. Minimap2: pairwise alignment for nucleotide sequences. *Bioinformatics*. 2018;34(18): 3094-3100.
26. Ruan J, Li H. Fast and accurate long-read assembly with wtdbg2. *Nat Methods*. 2020;17(2):155-158.
27. Gong G, Xiong Y, Xiao S, et al. Origin and chromatin remodeling of young X/Y sex chromosomes in catfish with sexual plasticity. *Natl Sci Rev*. 2023;10(2): nwac239.
28 filterx software https://github.com/ruanjue/filterx Accessed 15 March 2024
29. biallelic SNV datasets from 1000 genomes project. FTP accesss: http://ftp.1000genomes.ebi.ac.uk/vol1/ftp/data_collections/1000_genomes_project/release/20190312_biallelic_SNV_and_INDEL/
30. Gurevich A, Saveliev V, Vyahhi N, et al. QUAST: quality assessment tool for genome assemblies. *Bioinformatics*. 2013;29(8):1072-1075.
31. dotPlotly software https://github.com/tpoorten/dotPlotly Accessed 15 March 2024
32. Shafin K, Pesout T, Lorig-Roach R, et al. Nanopore sequencing and the Shasta toolkit enable efficient de novo assembly of eleven human genomes. *Nat Biotechnol*. 2020;38(9):1044-1053.
33. Human pangenomics stored in Amazon Simple Storage Service (Amazon S3). https://s3-us-west-2.amazonaws.com/human-pangenomics/index.html. Accessed 26 February 2024.
34. Ashkenazim trio data stored in The National Center for Biotechnology Information (NCBI). https://ftp-trace.ncbi.nlm.nih.gov/ReferenceSamples/giab/data/AshkenazimTrio. Accessed 26 February 2024.
35. Wang X. Ten genome assemblies of the human Y chromsome. Figshare. 2024.

https://doi.org/10.6084/m9.figshare.14564484 .
36. Wang X, Lu H, Liu Q, Li A, Zhou H, Zhang Y et al. Supporting data for "An effective strategy for assembling the sex-limited chromosome" GigaScience Database. 2024. http://dx.doi.org/10.5524/102511

Figure1

Click here to access/download;Figure;figure1.pdf

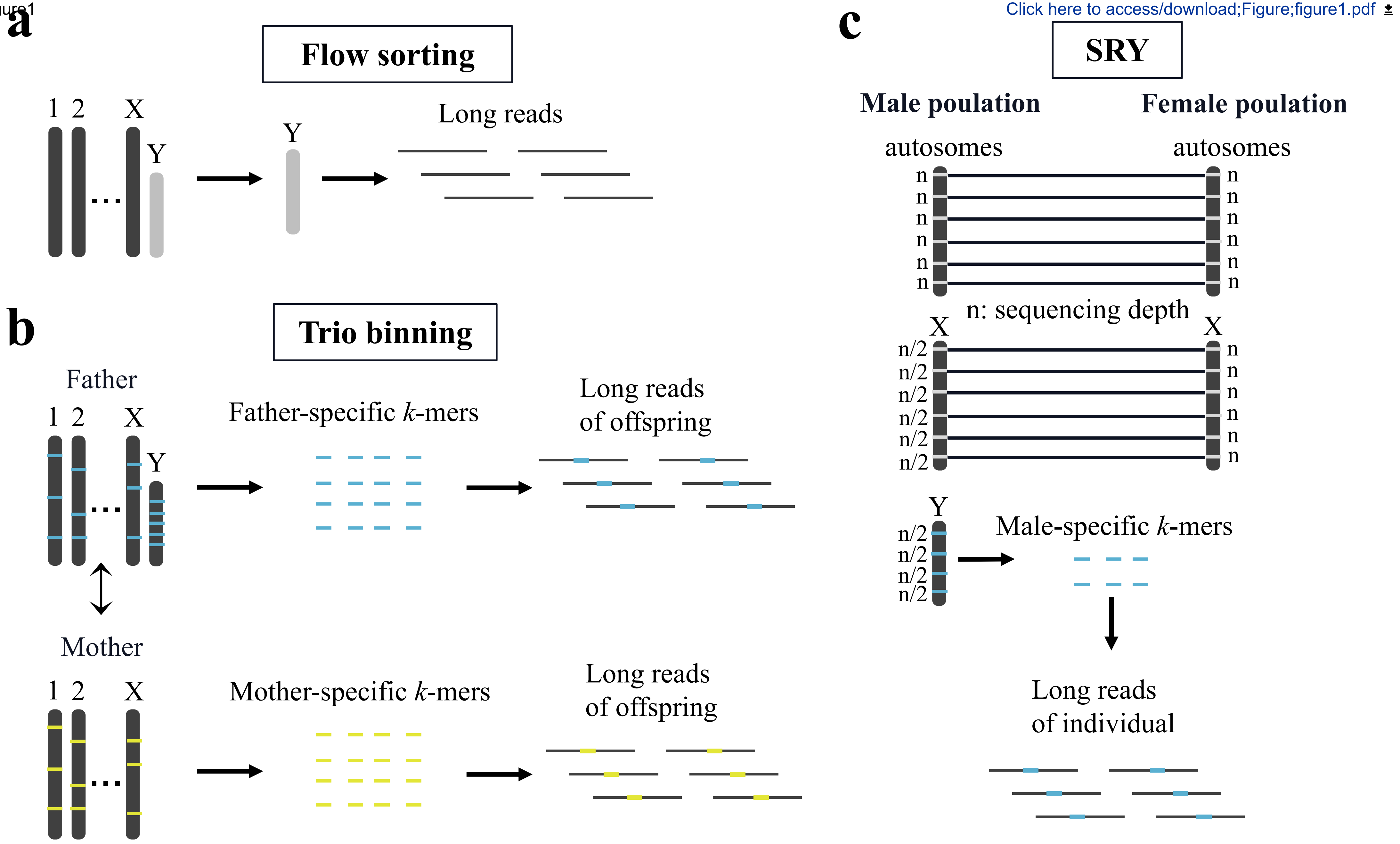

Figure2

Click here to access/download;Figure;figure2.pdf

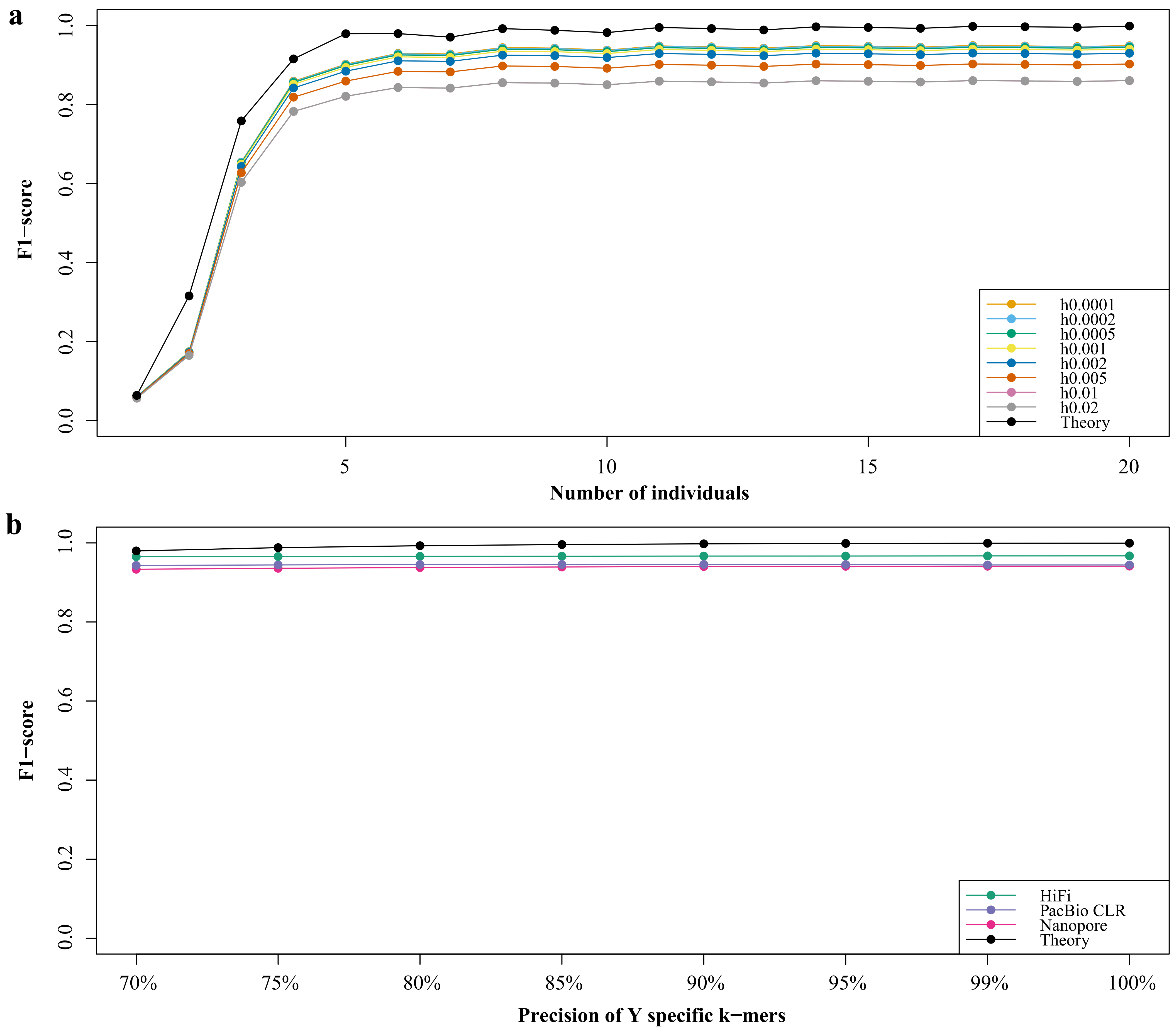

Figure3

Click here to access/download;Figure;figure3.pdf

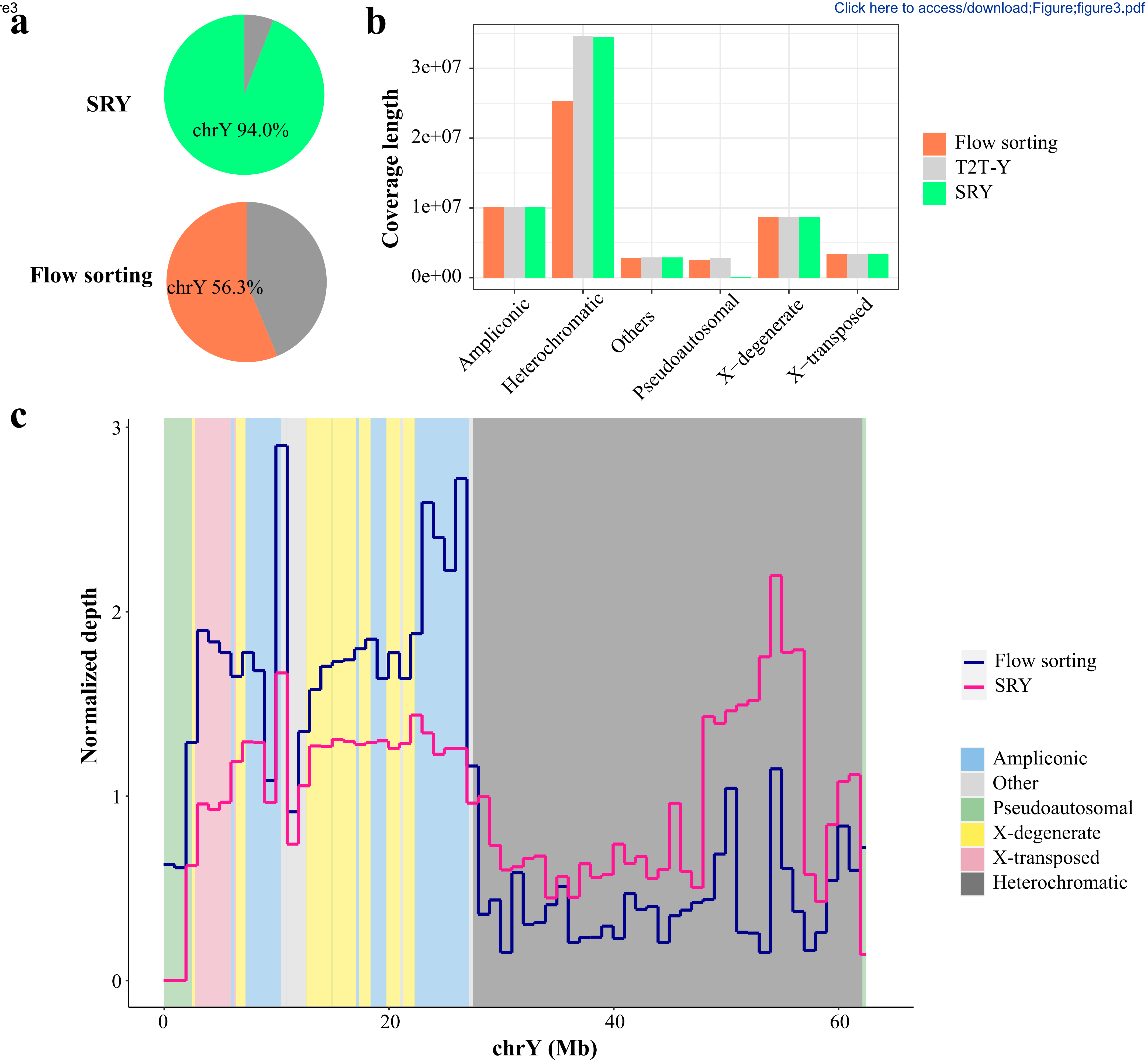

Figure4

Click here to access/download;Figure;figure4.pdf

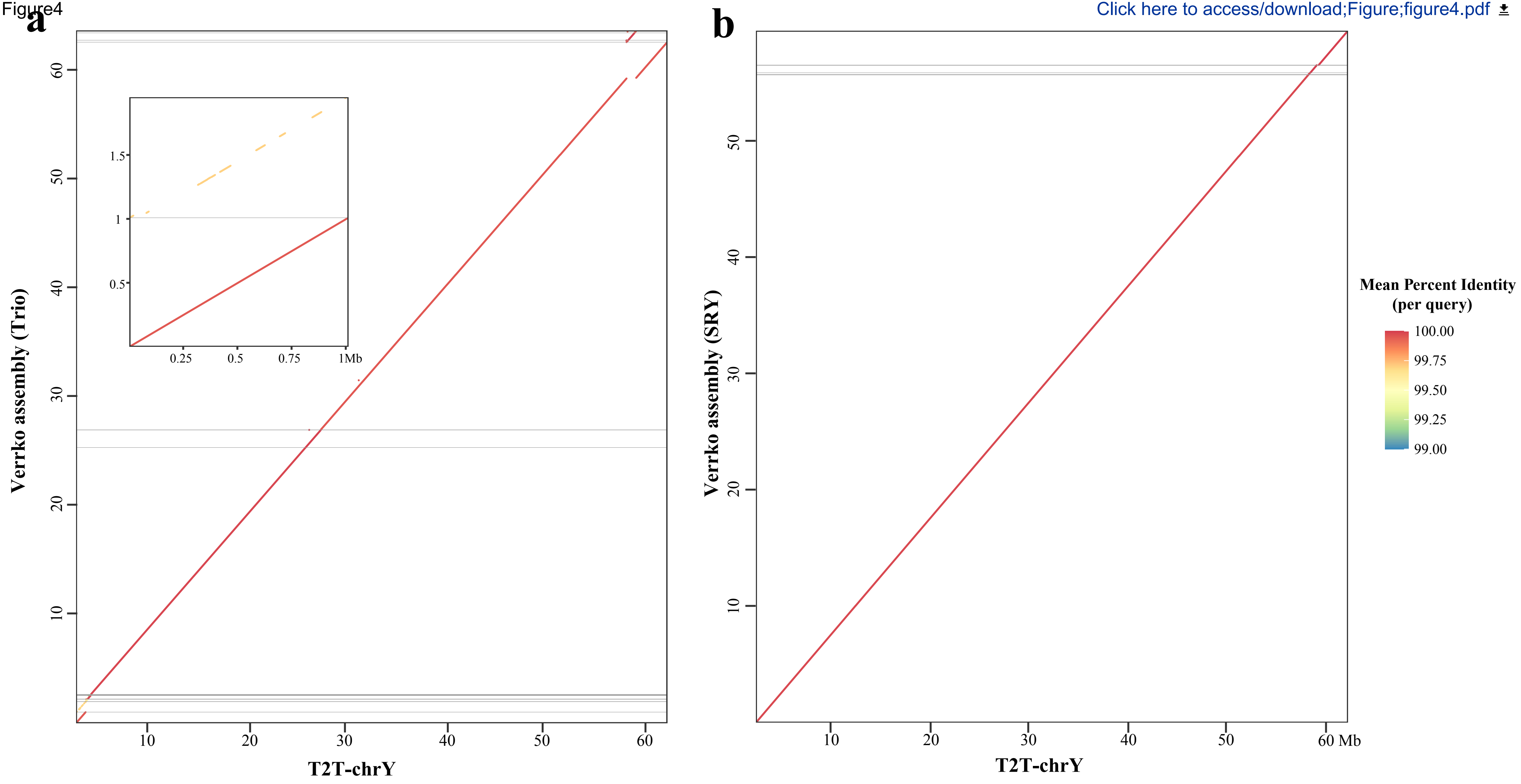

Click here to access/download
Supplementary Material
Supplemental-r1.docx

Personal Cover Click here to access/download;Personal Cover;CV-20240227.docx

Dear Hans,

We have carefully incorporated the suggestions provided by both yourself and the reviewers, ensuring that the revised content aligns with the standards and objectives of the journal. We appreciate the time and effort invested by you and the reviewers in evaluating our work, and we are grateful for the constructive feedback provided. Thank you for your continued support!

Sincerely,
Xiaobo

Response to Reviewers

Reviewer #1: After reading the revised article, the questions I had previously posed were answered. I am very interested in this SRY method and believe it is also an important part of sex chromosome research. From my personal point of view, it is not easy to collect Trio data for most species except a few, but it is relatively easy to collect HIC data. It would be helpful if the authors could also compare the results of SRY HIFI with those of Hifiasm (HIC phased) to help people choose the right tool for sex chromosome assembly. However, this is not necessary, because SRY has achieved a very good result in humans. Overall, the data and results are convincing.

Response: Thank you for your thoughtful feedback and interest in our SRY method for sex chromosome research. We will consider incorporating Hi-C data in future versions of SRY and compare with those of Hifiasm or Verkko (HiC phased).

Reviewer #3: The authors have addressed most of my concerns. The revised manuscript reads much better than before.

Regarding my last comment and response from the authors about the W chromosome, I was hoping to see comparable coverage of the W chromosome to the reference, as a proof of principle that SRY could be applied to non-human, highly diverged genomes. The assembly looks very fragmented though. Was it only the similarity to the Z chromosome that caused the fragmentation? Are there no other factors contributing to the discontinuity of the W chromosome?

Response: In response to your question about the fragmentation of the W chromosome in our study, we would like to provide some clarifications. We sorted the W chromosome at a coverage of 14.0X, which is slightly lower than the reference genome's coverage of 18.3X. In highly diverged genomes, the similarity between the W and Z chromosomes primarily affects the assembly of the pseudoautosomal region (PAR).

One contributing factor to the fragmentation of the W chromosome assembly is the non-uniform distribution of specific markers. HiFi sequencing data that is predominantly located in regions with lower marker density is difficult to separate, leading to some degree of data loss.

The PacBio CLR data from the 2021 Nature article was at 47.5X for the W

chromosome (assembling contig size: 20.0Mb, contig N50: 1.5Mb), while the HiFi sequencing data was less than half of that (assembling contig size: 19.7Mb, contig N50: 0.5Mb). The difference in sequencing data quantity may also have contributed to the fragmented assembly observed.

A few minor comments below to the revised version:

1. Please indicate which genome was compared in the legend of Supp. Table 5.

Response: The genome compared in the legend of Supplementary Table 5 is HG01109. We have made the modification to indicate this.

2. When using `et al` notations, please use the last name. Mari et al should be Serra Mari et al., Mikko et al should be Rautiainen et al. Also, Serra Mari et al is now published in Genome Biology: https://doi.org/10.1186/s13059-023-03160-z. Please update the reference.

Response: Thank you for the clarification. We have now made the adjustments to use the last names when using et al notations. Additionally, we have updated the reference for Serra Mari et al.

3. There are a few grammar corrections to make.

Response: We have made the grammar corrections in the text and highlighted them in blue.
