# Supplementary material for: An effective strategy for assembling the sex-limited chromosome
Source: Gigascience. 2024 Apr 16;13:giae015. doi: 10.1093/gigascience/giae015 (PMC11020242; doi:10.1093/gigascience/giae015)
Supplement: giae015_Supplemental_File [file giae015_supplemental_file.docx]

| **Male sample** | **SRA number** | **Bases** | **Depth (X)** |
| --- | --- | --- | --- |
| NA18558 | ERR1044230 | 16,183,315,278 | 5.4 |
| NA18561 | ERR1044240 | 15,013,370,244 | 5.0 |
| NA18562 | ERR1044260 | 18,121,069,260 | 6.0 |
| NA18563 | ERR1044350 | 16,009,295,342 | 5.3 |
| NA18572 | ERR1044440 | 16,942,192,260 | 5.6 |
| NA18603 | ERR1044590 | 18,385,962,122 | 6.1 |
| NA18605 | ERR1044600 | 14,444,601,932 | 4.8 |
| NA18608 | ERR1044630 | 11,101,817,640 | 3.7 |
| NA18609 | ERR1044640 | 15,506,301,118 | 5.2 |
| NA18524 | ERR1045130 | 15,304,505,942 | 5.1 |
| NA18611 | ERR1055330 | 16,234,577,616 | 5.4 |
| NA18612 | ERR1055350 | 15,882,531,744 | 5.3 |
| NA18620 | ERR1055360 | 15,552,942,580 | 5.2 |
| NA18621 | ERR1055380 | 15,869,728,412 | 5.3 |
| NA18622 | ERR1055400 | 14,523,844,116 | 4.8 |
| NA18623 | ERR1055410 | 15,793,786,282 | 5.3 |
| NA18624 | ERR1055430 | 18,739,740,352 | 6.2 |
| NA18632 | ERR1055450 | 13,739,485,070 | 4.6 |
| NA18633 | ERR1055460 | 15,773,888,932 | 5.3 |
| NA18635 | ERR1055480 | 16,950,062,080 | 5.7 |
| NA18636 | ERR1055500 | 14,921,180,350 | 5.0 |
| NA18637 | ERR1055530 | 15,657,761,144 | 5.2 |
| HG00418 | ERR1044270 | 17,201,399,072 | 5.7 |
| HG00421 | ERR1044280 | 14,366,604,830 | 4.8 |
| HG00427 | ERR1044310 | 21,610,409,840 | 7.2 |
| HG00448 | ERR1044330 | 20,115,083,604 | 6.7 |
| HG00436 | ERR1044660 | 16,616,616,426 | 5.5 |
| HG00442 | ERR1044680 | 20,650,338,700 | 6.9 |
| HG00472 | ERR1044690 | 19,143,414,826 | 6.4 |
| HG00475 | ERR1044720 | 16,259,881,464 | 5.4 |
| HG00478 | ERR1044740 | 15,824,391,668 | 5.3 |
| HG00500 | ERR1044770 | 24,112,184,098 | 8.0 |
| HG00524 | ERR1044790 | 16,700,039,750 | 5.6 |
| HG00530 | ERR1044820 | 11,388,389,318 | 3.8 |
| HG00533 | ERR1044840 | 16,062,632,506 | 5.4 |
| HG00536 | ERR1044860 | 19,376,589,952 | 6.5 |
| HG00542 | ERR1044890 | 14,956,118,832 | 5.0 |
| HG00556 | ERR1044900 | 12,672,490,040 | 4.2 |
| HG00580 | ERR1044930 | 15,663,073,246 | 5.2 |
| HG00583 | ERR1044940 | 15,188,594,020 | 5.1 |
| HG00619 | ERR1044960 | 11,342,085,564 | 3.8 |
| HG00653 | ERR1044990 | 15,258,572,322 | 5.1 |
| HG00656 | ERR1045000 | 2,658,418,048 | 0.9 |
| HG00671 | ERR1045040 | 8,329,426,632 | 2.8 |
| HG00683 | ERR1045070 | 10,073,120,170 | 3.4 |
| HG00698 | ERR1045100 | 22,076,505,468 | 7.4 |
| **Total** |  | **724,298,340,212** | **241.4** |

**Supplementary Table 1. S****ummary of datasets for 46 males of Han Chinese population.**

|  | **SRA number** | **Bases** | **Depth (X)** |
| --- | --- | --- | --- |
| NA18547 | ERR1044160 | 16,620,329,628 | 5.5 |
| NA18550 | ERR1044170 | 16,092,555,260 | 5.4 |
| NA18552 | ERR1044190 | 8,803,577,000 | 2.9 |
| NA18555 | ERR1044210 | 17,800,123,846 | 5.9 |
| NA18564 | ERR1044370 | 15,474,012,956 | 5.2 |
| NA18566 | ERR1044390 | 14,740,529,084 | 4.9 |
| NA18570 | ERR1044400 | 13,075,355,446 | 4.4 |
| NA18571 | ERR1044420 | 17,249,833,170 | 5.7 |
| NA18573 | ERR1044450 | 16,341,025,802 | 5.4 |
| NA18576 | ERR1044470 | 19,323,473,098 | 6.4 |
| NA18577 | ERR1044480  ERR1044500  ERR1044520  ERR1044530  ERR1044550  ERR1044570 | 14,874,069,510 | 5.0 |
| NA18579 | ERR1044500  ERR1044520  ERR1044530  ERR1044550  ERR1044570 | 18,302,755,678 | 6.1 |
| NA18582 | ERR1044520 | 16,168,604,708 | 5.4 |
| NA18592 | ERR1044530 | 14,863,097,166 | 5.0 |
| NA18593 | ERR1044550 | 12,690,249,342 | 4.2 |
| NA18594 | ERR1044570 | 18,327,381,930 | 6.1 |
| NA18526 | ERR1045150 | 17,030,015,572 | 5.7 |
| NA18529 | ERR1045160 | 12,384,112,060 | 4.1 |
| NA18532 | ERR1045180 | 18,189,286,824 | 6.1 |
| NA18537 | ERR1045190 | 15,361,339,434 | 5.1 |
| NA18540 | ERR1045210 | 15,333,497,190 | 5.1 |
| NA18542 | ERR1045230 | 15,679,915,202 | 5.2 |
| NA18545 | ERR1045240 | 11,301,517,136 | 3.8 |
| HG00422 | ERR1044300 | 19,746,486,008 | 6.6 |
| HG00443 | ERR1044320 | 22,068,330,434 | 7.4 |
| HG00428 | ERR1044650 | 15,488,970,832 | 5.2 |
| HG00437 | ERR1044670 | 17,726,963,840 | 5.9 |
| HG00473 | ERR1044710 | 16,835,674,104 | 5.6 |
| HG00476 | ERR1044730 | 21,576,005,818 | 7.2 |
| HG00479 | ERR1044750 | 13,225,405,954 | 4.4 |
| HG00513 | ERR1044780 | 14,680,930,762 | 4.9 |
| HG00525 | ERR1044800 | 17,847,243,022 | 5.9 |
| HG00531 | ERR1044830 | 19,210,626,610 | 6.4 |
| HG00534 | ERR1044850 | 21,754,095,050 | 7.3 |
| HG00537 | ERR1044880 | 12,567,250,436 | 4.2 |
| HG00557 | ERR1044910 | 12,441,783,544 | 4.1 |
| HG00584 | ERR1044950 | 19,005,002,818 | 6.3 |
| HG00620 | ERR1044980 | 20,458,327,948 | 6.8 |
| HG00657 | ERR1045020 | 14,947,538,542 | 5.0 |
| HG00663 | ERR1045030 | 22,490,781,150 | 7.5 |
| HG00672 | ERR1045050 | 20,373,837,034 | 6.8 |
| HG00684 | ERR1045080 | 20,319,812,540 | 6.8 |
| HG00690 | ERR1045090 | 18,285,235,556 | 6.1 |
| HG00699 | ERR1045110 | 16,960,983,944 | 5.7 |
| **Total** |  | **734,037,942,988** | **244.7** |

**Supplementary Table 2.** **Summary of datasets for 44 females of Han Chinese population.**

| **Sample** | **Long-read**  **Category** | **Sorted** length | **Average** Sorted length | **Sorted Count** | **Total length** | **Average length** | **Count** | **Average error rate** |
| --- | --- | --- | --- | --- | --- | --- | --- | --- |
| **HG002** | **P** | 1.5G | 8.1K | 190,858 | 234.7G | 7.9K | 29,809,956 | 14.3% |
|  | **N** | 1.8G | 16.8K | 104,146 | 184.2G | 13.6K | 13,586,420 | 10.4% |
| **HG00**3 | **P** | 0.7G | 7.7K | 93,368 | 111.9G | 7.5K | 14,851,293 | 14.5% |
|  | **N** | 2.0G | 11.9K | 167,110 | 272.6G | 11.4K | 23,954,632 | 14.5% |
| **HG00**5 | **P** | 1.5G | 10.4K | 142,173 | 201.3G | 9.2K | 21,933,756 | 14.3% |
|  | **N** | 1.5G | 28.1K | 51,927 | 181.3G | 26.5K | 6,839,693 | 10.7% |
| **HG006** | **P** | 0.6G | 11.4K | 55,647 | 86.7G | 9.4K | 9,186,765 | 14.7% |
|  | **N** | 1.3G | 28.0K | 47,306 | 162.5G | 26.4K | 6,162,748 | 10.0% |
| **HG01109** | **N** | 1.3Gb | 5.3K | 251,676 | 219.4G | 8.1K | 26,975,771 | 11.1% |
| **HG01243** | **N** | 1.0G | 7.5K | 132,190 | 187.1G | 11.5K | 16,293,849 | 10.9% |
| **HG02055** | **N** | 1.3G | 22.9K | 54,771 | 202.4G | 20.0K | 10,141,336 | 12.3% |
| **HG03098** | **N** | 1.1G | 10.5K | 109,862 | 177.0G | 11.1K | 15,898,550 | 12.9% |
| **HG03492** | **N** | 0.9G | 3K | 293,576 | 157.5G | 4.4K | 35,999,328 | 11.5% |
| **HX1** | **P** | 2.9G | 8.3K | 345,760 | 731.7G | 8.4K | 87,485,724 | 17.0% |
|  | **N** | 0.8G | 16.1K | 48,686 | 100.3G | 16.0K | 6,255,000 | 15.0% |

**Supplementary Table 3. Statistics of PacBio and Nanopore reads identified by SRY from long-read sequencing datasets.** P represents PacBio CLR reads, and N represents Nanopore reads. The average error rate is calculated based on the alignment results of the third-generation data on the human T2T genome.

|  | | **Flow sorting** | **SRY (HG01109)** | **SRY (nine individuals)** |
| --- | --- | --- | --- | --- |
| **All** | **Total length** | 22,000,118 | 22,866,849 | 17,705,259-22,866,849 |
|  | **N50** | 1,572,692 | 1,529,669 | 1,418,447-2,333,095 |
|  | **Autosome+X aligned length** | 1,846,274 (8.4%) | 497,792 (2.2%) | 246,997-578,565 (1.2%-2.9%) |
|  | **Y aligned length** | 18,577,804 | 19,713,509 | 16,737,249-20,388,526 |
|  | **NA50** | 710,714 | 711,640 | 606,225-1,138,334 |
|  | **NA75** | 104,485 | 42,458 | 42,458-260,602 |
|  | **Mismatches (/100kbp)** | 263.80 | 267.55 | 175.56-294.71 |
|  | **Indels (/100kbp)** | 189.54 | 194.58 | 109.41-221.67 |
| **Ampliconic** | **Aligned length** | 6,471,932 | 6,332,466 | 4,755,420-6,332,466 |
|  | **Mismatches (/100kbp)** | 358.03 | 497.48 | 372.61-546.87 |
|  | **Indels (/100kbp)** | 165.60 | 229.55 | 84.39-229.55 |
| **X-degenerate** | **Aligned length** | 8,884,024 | 8,810,648 | 8,283,581-8,883,474 |
|  | **Mismatches (/100kbp)** | 164.47 | 152.84 | 124.91-161.83 |
|  | **Indels (/100kbp)** | 78.46 | 87.7 | 41.79-132.41 |
| **X-transposed** | **Aligned length** | 3,880,237 | 3,862,338 | 3,675,209-3,862,338 |
|  | **Mismatches (/100kbp)** | 623.08 | 602.77 | 497.26-602.77 |
|  | **Indels (/100kbp)** | 112.86 | 166.23 | 56.35-184.35 |
| **Pseudoautosomal** | **Aligned length** | 2,711,039 | 640,462 | 494,367-640,462 |
|  | **Mismatches (/100kbp)** | 1,414.61 | 14,258.98 | 13,728.13-29,498.05 |
|  | **Indels (/100kbp)** | 259.42 | 850.6 | 850.6-1,800.38 |
| **Heterochromatic** | **Aligned length** | 70,190 | 1,210,886 | 243,487-2,607,300 |
|  | **Mismatches (/100kbp)** | 3,360.46 | 4,705.24 | 1,993.89-4,705.24 |
|  | **Indels (/100kbp)** | 830.67 | 1,465.1 | 562.99-1,961.29 |
| **Others** | **Aligned length** | 1,386,458 | 1,490,295 | 1,300,738-1,777,989 |
|  | **Mismatches (/100kbp)** | 2,104.37 | 2,154.25 | 1,891.85-2,529.37 |
|  | **Indels (/100kbp)** | 238.67 | 462.89 | 462.89-471.89 |

**Supplementary Table 4. Comparison of the assembled genomes for SRY and flow sorting.** Human sample numbers are in parentheses, and the sample numbers and metrics of the other nine individuals are detailed in supplementary table 6.

|  | | **SRY (with Trio binning)** | **SRY (with WGS)** |
| --- | --- | --- | --- |
| **All** | **Total length** | 15,161,111 | 14,591,512 |
|  | **N50** | 1,208,828 | 507,517 |
|  | **Autosome+X aligned length** | 288,974 (2.0%) | 2,737 (0.0%) |
|  | **Y aligned length** | 14,058,622 | 10,127,949 |
|  | **NA50** | 501,025 | 290,040 |
|  | **NA75** | 197,270 | 166,322 |
|  | **Mismatches (/100kbp)** | 333.66 | 160.19 |
|  | **Indels (/100kbp)** | 316.04 | 150.95 |
| **Ampliconic** | **Aligned length** | 3,747,647 | 3,986,246 |
|  | **Mismatches (/100kbp)** | 743.74 | 474.86 |
|  | **Indels (/100kbp)** | 314.39 | 215.17 |
| **X-degenerate** | **Aligned length** | 7,715,598 | 6,378,403 |
|  | **Mismatches (/100kbp)** | 327.39 | 149.71 |
|  | **Indels (/100kbp)** | 291.2 | 112.38 |
| **X-transposed** | **Aligned length** | 3,579,267 | 356,321 |
|  | **Mismatches (/100kbp)** | 807.24 | 24,596.98 |
|  | **Indels (/100kbp)** | 387.48 | 586.9 |
| **Pseudoautosomal** | **Aligned length** | 413,077 | 319,043 |
|  | **Mismatches (/100kbp)** | 28,196.73 | 44,693.71 |
|  | **Indels (/100kbp)** | 1,062.47 | 1,287.86 |
| **Heterochromatic** | **Aligned length** | 7,892 | 7,030 |
|  | **Mismatches (/100kbp)** | 11,031.86 | 11,479.59 |
|  | **Indels (/100kbp)** | 713.27 | 561.22 |
| **Others** | **Aligned length** | 396,649 | 335,380 |
|  | **Mismatches (/100kbp)** | 14,215.11 | 14,183.14 |
|  | **Indels (/100kbp)** | 773.11 | 591.64 |

**Supplementary Table 5. Contig sorting for the HG01109 assembly results of Trio binning and WGS using SRY**.

|  |  | **HG002** | **HG003** | **HG005** | **HG006** | **HG01243** | **HG02055** | **HG03098** | **HG03492** | **HX1** |
| --- | --- | --- | --- | --- | --- | --- | --- | --- | --- | --- |
| **All** | **Total length** | 20,335,142 | 22,081,712 | 21,380,466 | 20,729,667 | 19,642,433 | 20,157,635 | 19,032,641 | 17,705,259 | 19,522,068 |
|  | **Autosome+X aligned length** | 459,255 (2.3%) | 436,275 (2.0%) | 273,823 (1.3%) | 246,997 (1.2%) | 578,565 (2.9%) | 429,428 (2.1%) | 375,641 (2.0%) | 441,968 (2.5%) | 376,561 (1.9%) |
|  | **Y aligned length** | 19,013,516 | 20,388,526 | 19,897,468 | 19,152,430 | 17,915,111 | 18,201,566 | 17,903,852 | 16,737,249 | 18,472,360 |
|  | **NA50** | 1,031,159 | 1,138,334 | 1,024,720 | 861,966 | 658,593 | 654,698 | 881,985 | 606,225 | 852,144 |
|  | **NA75** | 158,201 | 129,967 | 200,600 | 157,602 | 172,045 | 141,155 | 173,710 | 260,602 | 180,413 |
|  | **Mismatches (/100kbp)** | 237.41 | 294.71 | 175.56 | 208.22 | 192.02 | 182.89 | 192.32 | 194.92 | 211.39 |
|  | **Indels (/100kbp)** | 124.30 | 186.66 | 109.41 | 131.17 | 160.82 | 151.72 | 153.90 | 169.43 | 221.67 |
| **Ampliconic** | **Aligned length** | 5,185,696 | 5,605,806 | 5,674,486 | 5,514,962 | 5,180,604 | 5,289,467 | 5,446,598 | 4,755,420 | 5,307,346 |
|  | **Mismatches (/100kbp)** | 546.87 | 545.06 | 372.61 | 398.47 | 412.64 | 417.62 | 419.46 | 508.73 | 421.65 |
|  | **Indels (/100kbp)** | 120.79 | 136.75 | 84.39 | 114.26 | 143.52 | 138.06 | 165.15 | 216.74 | 167.06 |
| **X-degenerate** | **Aligned length** | 8,842,866 | 8,833,482 | 8,883,474 | 8,771,384 | 8,758,444 | 8,713,075 | 8,768,041 | 8,283,581 | 8,717,719 |
|  | **Mismatches (/100kbp)** | 124.91 | 129.65 | 134.93 | 143.09 | 132.69 | 144.94 | 149.17 | 148.32 | 161.83 |
|  | **Indels (/100kbp)** | 41.79 | 54.74 | 48.51 | 69.64 | 96.74 | 83.17 | 88.02 | 93.85 | 132.41 |
| **X-transposed** | **Aligned length** | 3,831,417 | 3,801,010 | 3,846,454 | 3,764,083 | 3,727,055 | 3,747,557 | 3,780,800 | 3,675,209 | 3,747,765 |
|  | **Mismatches (/100kbp)** | 543.41 | 520.12 | 569.87 | 552.89 | 567.23 | 576.54 | 575.67 | 497.26 | 575.94 |
|  | **Indels (/100kbp)** | 56.35 | 65.98 | 61.74 | 110.76 | 142.40 | 144.74 | 152.42 | 151.42 | 184.35 |
| **Pseudoautosomal** | **Aligned length** | 592,370 | 597,696 | 605,290 | 588,606 | 570,957 | 606,243 | 561,276 | 494,367 | 517,705 |
|  | **Mismatches (/100kbp)** | 21,261.58 | 16,993.84 | 20,595.11 | 19,669.58 | 18,200.38 | 13,728.13 | 23,201.82 | 22,649.09 | 29,498.05 |
|  | **Indels (/100kbp)** | 1,116.35 | 1,205.36 | 1,017.24 | 946.59 | 1,128.17 | 1,218.03 | 1,644.79 | 1,551.51 | 1,800.38 |
| **Heterochromatic** | **Aligned length** | 1,487,386 | 2,607,300 | 1,260,370 | 1,006,909 | 567,725 | 626,560 | 402,560 | 243,487 | 884,498 |
|  | **Mismatches (/100kbp)** | 2,936.44 | 3,908.44 | 2,079.06 | 2,737.91 | 4,400.85 | 3,352.67 | 2,799.32 | 1,993.89 | 3,235.14 |
|  | **Indels (/100kbp)** | 1,147.93 | 1,767.34 | 562.99 | 793.50 | 1,109.37 | 909.56 | 1,012.84 | 778.17 | 1,961.29 |
| **Others** | **Aligned length** | 1,533,940 | 1,527,417 | 1,777,989 | 1,559,190 | 1,367,623 | 1,375,389 | 1,385,124 | 1,300,738 | 1,401,011 |
|  | **Mismatches (/100kbp)** | 2,129.03 | 2,529.37 | 1,891.85 | 2,094.76 | 2,433.10 | 2,210.43 | 2,412.92 | 2,178.21 | 2,078.12 |
|  | **Indels (/100kbp)** | 463.89 | 464.89 | 465.89 | 466.89 | 467.89 | 468.89 | 469.89 | 470.89 | 471.89 |

**Supplementary Table 6. Statistics of nine human assemblies.**
