# Supplementary material for: An effective strategy for assembling the sex-limited chromosome
Source: Gigascience. 2024 Apr 16;13:giae015. doi: 10.1093/gigascience/giae015 (PMC11020242; doi:10.1093/gigascience/giae015)
Supplement: giae015_Response_to_Reviewer_Comments_Original_Submission [file giae015_response_to_reviewer_comments_original_submission.pdf]

Author's Response To Reviewer Comments

Dear Hans,

I am writing to submit a revised version of our manuscript entitled “An effective strategy for assembling the sex-limited chromosome”. We would like to thank the reviewers for their insightful comments and constructive feedback.

In response to the reviewers' comments, we have carefully revised the manuscript to address all their concerns. Specifically, we have focused on incorporating the suggestions from the reviewers and addressing the issues raised in Reviewer 3's comment #6. We have included a detailed explanation of our approach in the revised manuscript, which we hope will clarify any confusion that may have arisen.

We believe that these changes have significantly improved the quality of our manuscript, and we hope that you will find it suitable for publication in GigaScience.

Thank you for considering our revised manuscript.

Sincerely,
Xiaobo Wang

Response to reviewers
Reviewer 1:

The authors have introduced a novel bioinformatic approach for sex chromosome assembly, addressing a persistently challenging problem in genomics. This method harnesses the full potential of whole-genome resequencing data without necessitating supplementary experimental procedures, rendering it applicable to a wide array of non-model species. Notably, the method exhibits robustness when applied to human data, surpassing established techniques such as flow-sorting and trio-binning. While the manuscript exhibits promise, several key aspects warrant refinement and elucidation to bolster its consideration for publication in GigaScience.
Response: Thank you for your insightful comments on our manuscript. We appreciate your recognition of the novelty of our bioinformatic approach for sex chromosome assembly and its potential impact on genomics research. We have provided a detailed response to each of your suggestions below.

1. Language Polishing: A degree of language refinement is advisable to enhance the overall clarity and professionalism of the manuscript.
Response: Thank you for your feedback. We have carefully revised the manuscript based on your suggestions as well as those of other reviewers. The modified sections are indicated in blue font.

2. Y Chromosome Assembly Discrepancy: The authors should acknowledge and provide an explanation for the substantial difference between the length of the latest Y chromosome assembly from T2T (~62Mb) and the assembly from SRY with Verkko (~23Mb), as detailed in Table 1.
Response: Thank you for your comment. The Flow sorting method was used to measure only Nanopore data. In order to make a fair comparison with the Flow sorting method, we selected only Nanopore or PacBio data from 10 individuals, which resulted in an assembly of only around 23Mb. We have already mentioned this limitation in the Discussion section of the manuscript.
In order to obtain a complete Y chromosome assembly and to compare with the trio mode of the verkko software, we used long-read Nanopore and HiFi data, which ultimately resulted in a ~60Mb assembly (Figure 4, SRY with Verkko) .
Therefore, the difference in assembly size is mainly due to data sources.

3. Y Chromosome Completeness: In cases where the Y chromosome assembly is incomplete, the inclusion of a figure or table delineating the proportion that SRY can recover in distinct regions of the Y chromosome would be beneficial. This could facilitate a comparative analysis of the method's efficacy across different

regions.
Response: Thank you for the clarification. I apologize for any confusion caused. As mentioned in the previous response, we were able to obtain an almost complete Y chromosome assembly using long-read Nanopore and HiFi data. However, for the purpose of a fair comparison with the Flow sorting method, we obtained an incomplete assembly and displayed the assembly statistics for different regions in Table 1. To focus our discussion on the complete Y chromosome assembly, we have made the decision to present Table 1 as a supplementary material.

4. Figure 4 Clarity: It is imperative to label the coordinates on both the X and Y axes in Figure 4 to enhance clarity. While Figure 4 suggests that the assembly from SRY is complete compared to T2T-CHM13, the total length of the SRY assembly (approximately 23Mb) should be clearly reconciled with this observation.
Response: Thank you for your feedback. We have updated the figure to include clear coordinate labels on both axes to enhance clarity (below).

Fig. 4: Verkko assembly using Trio or SRY

5. Table 1 Organization: The organization of Table 1 should be improved to enhance readability and comprehensibility.
Response: Thank you for your suggestion. We have made improvements to enhance the readability and comprehensibility of Table 1. We have split it into two separate tables, labeled as Supplementary Table 4 and Supplementary Table 5, which are included as supplementary material.
Furthermore, we have removed some less relevant information from the tables to ensure clarity and focus on the key details.

6. MSK-Based Read Filtering: Authors should explicitly address the potential exclusion of reads from Y regions with lower than average MSK, especially in species with both young and old parts on Y chromosomes. If possible, provide recommendations or strategies for rescuing such reads.
Response: Regarding the potential exclusion of reads from Y regions with lower than average MSK (Male Specific K-mer), our intention in using average MSK as a threshold is to reduce false positives caused by sequencing errors or other factors.
In the case of humans, the differentiation time between the Y and X chromosomes is estimated to be around 300 million years ago (old Y). In our human simulated dataset (~25X coverage for the Y chromosome), we successfully captured approximately 24X (95.6%) of the Y chromosome using HiFi data and approximately 23X (91.5%) using Nanopore data.
For yellow catfish (detailed in Response to Q7), which contains a young Y chromosome with a similarity of over 99% to the X chromosome, we simulated 50X coverage of HiFi data and 50X coverage of ultra-long Nanopore data. Using SRY, we captured approximately 46X (92.2%) of the Y chromosome with HiFi data and approximately 37X (73.2%) with ultra-long Nanopore data.
Therefore, for Y chromosomes that contain both old and young parts, we recommend prioritizing HiFi data as the loss of data will have minimal impact on assembly. If feasible, we also suggest including ultra-long Nanopore data to increase the completeness of the assembly results.

7. Simulation for species with young sex chromosomes: It is essential to conduct additional simulations for testing the efficiency of isolating Y reads for species with young sex chromosomes. This analysis should consider the variation between X and Y chromosomes, aiding researchers in evaluating the method's suitability for their specific study organisms.
Response: We have conducted simulations to test the efficiency of isolating Y reads for species with young sex chromosomes, using the yellow catfish as a model organism [1]. This species contains a young Y chromosome with a size of approximately 43.2 Mb, which shares greater than 99% similarity with the X chromosome. In our simulations, we generated 100X HiFi and Nanopore data (50X for the Y chromosome) and obtained 46X of HiFi and 37X of ultra-long Nanopore data after sorting for the Y chromosome. Using the verkko software, we assembled the Y chromosome into two contigs with a total size of 42.7 Mb. These results demonstrate that our method is effective for assembling the Y chromosome of species with young sex chromosomes.We have incorporated these results into our manuscript.

[1] Gong G, Xiong Y, Xiao S, et al. Origin and chromatin remodeling of young X/Y sex chromosomes in catfish with sexual plasticity. National Science Review, 2023, 10(2): nwac239.

Addressing these points will further strengthen the manuscript's scientific rigor and its suitability for publication in GigaScience.
Response: Thank you for your valuable feedback.

Reviewer 2:
The SRY method, developed and evaluated for sorting long reads of sex-limited chromosomes, has shown promise in effectively identifying and sorting sequences based on sex-specific markers, particularly the Y chromosome. These sorted long reads are then utilized for genome assembly. Additionally, the SRY method can be used to select Y chromosome contigs from a male individual's whole genome assembly.Overall, the success of SRY in sorting and assembling long reads of sex-limited chromosomes highlights its potential as an alternative to experimental methods for studying sex-specific genomic regions.
Response: Thank you for your positive comments.

Here are some comments for further improvement of manuscript:
1) The authors may want to consider to presenting a table for standard evaluation metrics (k-mer or alignment-based). See Garg 2021 (https://genomebiology.biomedcentral.com/articles/10.1186/s13059-021-02328-9).
Response: Thank you for your suggestion. Table 1 presents the standard evaluation metrics for genome assembly using quast, an alignment-based software that is widely used for assessing genome assembly results. However, we have made some changes based on the feedback from other reviewers. The updated Table 1 has been moved to supplementary materials and is now labeled as Supplementary Table 4 and 5. We believe that these optimizations enhance the presentation of our results.

2) Adding a few important genes that are medically relevant and assembled properly may further add value to the work.
Response: In our study, we found that the assembly of gene sequences is generally not complex and can be relatively straightforward. Using the Verkko assembly method, both the direct assembly and the assembly after SRY sorting yielded comparatively complete genes on the Y chromosome. However, we observed that the assembly after sorting improved sequence contiguity, which is beneficial for analyzing regulatory sequences near the genes. We acknowledge that further investigation is needed to explore this aspect in future research. We appreciate your feedback.

Reviewer 3:
1.In the introduction, add recent marker based graph phasing algorithms in long-reads, such as hifiasm trio and verkko trio mode after the T2T-Y. They are different from trio-binning, which tries to phase the reads upfront. Graph based phasing is using markers to determine haplotype specific paths to traverse.
Response: Thank you for your feedback. In the introduction, we include recent marker-based graph phasing algorithms in long-reads, such as hifiasm trio and verkko trio mode, after discussing T2T-Y.

a. T2T-Y chromosome should be referencing Rhie et al., Nature 2023. Verkko is a successor of the manual efforts taken in T2T-Y, which should be also noted in the introduction.
Response: We have made the necessary updates to reference Rhie et al., Nature 2023 as the source for the T2T-Y chromosome. Additionally, we have included a note in the introduction to acknowledge that Verkko is a successor of the manual efforts undertaken in T2T-Y.

b. Reference for sexPhase program is still missing. Also, some rephrasing of the sentence is needed, as the way it is currently written is easily misleading to be understood as sexPhase was part of the methods used in the assembly of the T2T-Y.
Response: Thank you for pointing that out. The reference for the sexPhase program is a article titled "Genomes of the Banyan Tree and Pollinator Wasp Provide Insights into Fig-Wasp Coevolution," published in Cell, 2020. This software was specifically used for the assembly of the fig tree sex chromosomes mentioned in the article and does not have general applicability. Therefore, it is not suitable to be included as the latest development after T2T-Y. We have removed this content and added a description of marker-based graph phasing algorithms instead.

2. There are other approaches for phasing genomes taken in plants, for example the poly ploid potato

phasing using many siblings of the child by Mari et al. bioRxiv 2022.
Response: Thank you for your suggestion. We have incorporated the information into the Introduction section of our paper.

3. "But only one male and one female could suffer from sampling error" - this part is unclear. Please clarify.
Response: We apologize for the unclear wording in the previous version. We have since made revisions to the manuscript and have updated this section to read: "By incorporating population data into our analysis, we anticipate a reduction in the impact of sequencing coverage and allelic genotype variations when identifying Y chromosome-specific markers in a comparative analysis involving only two individuals of different genders." We hope that this revised statement is clearer and more accurately reflects our intended meaning.

4. Reference for the mason_simulator, badread software is missing.
Response: Corrected.

5. Provide the accession (HG02982) for the "African human Y" in the main text.
Response: Accession (HG02982) for "African human Y" has been added now.

6. I appreciate that the authors compared assemblies to T2T-Y as I requested before. However, fundamentally, mapping to T2T-Y and comparing length of each sequence classes is comparing apples to oranges, particularly in the heterochromatic region and ampliconic region of the Y. It is known to have variable copy numbers and size differences between two individuals. Frequent inversions have been reported in the ampliconic regions across different Y haplogroup. The number, size, and distribution of the repeat arrays composing the heterochromatic region has been shown to vary among different Y haplogroups in Hallast et al., Nature 2023. This can be also seen in Fig. 3c; the overall depth of the flow sorting in the heterochromatic region is below 1 - indicating the Yqh is shorter than T2T-Y, as it is in Fig. 3b. To make the benchmark legit, the authors should compare SRY and the flow sorting method using samples from the same individual. HG02982 and HX1 are presumably having very different sequence compositions given the diverged population history (African vs. Asian). Comparing total length of the assembled region against a 3rd different Y haplogroup (HG002Y) makes things more complicated, especially on regions that are known to vary a lot. If the authors think flow sorting based method needs to be compared, it should be benchmarked on the same individual to make an apple-to-apple comparison. I do agree results from read sorting (i.e. portion of reads sequenced from non-Y chromosomes in SRY vs. flow-sorting) is an important finding. However, I'd still argue comparing assemblies from the two different Y haplogroups is a stretch. The authors could have performed the same assembly length comparison on the T2T-Y using results from their SRY sorted reads with Verkko of HG002 vs. Verkko assembly using trio-binned markers.
Response: We completely agree with your points. As you mentioned, the comparison results from read sorting are indeed important findings. We acknowledge that using a different Y haplogroup for assembly length comparison may not be ideal. In our study, we did not have access to publicly available HG02982 whole genome long-read data, so we could only compare the SRY sorting results from another sample (HX1) to the flow sorting results. As a methodological study, we currently do not have the capability to collect HG02982 samples and perform third-generation sequencing. Even if we were able to obtain HG02982 samples, there could be inconsistencies in sequencing data length and accuracy due to updates in sequencing platforms, which would compromise the fairness of the comparison of the sorting results. However, we have taken your suggestion and compared and analyzed the sorting results of HG002 in the subsequent results section. In addition to your valuable feedback, we have also taken into consideration other suggestions from the reviewers. As a result, we have decided to move the Table 1 to the supplementary materials (Supplementary Table 4-6) to shift the focus on the assembly results of the complete Y chromosome. Thank you for your constructive comments, and we hope that the revised manuscript will meet your expectations.

7. In the section where assemblies are compared, the authors point to Table 1, which contains results from HG01109. HG01109 has never been mentioned before. I thought the authors were comparing assemblies from SRY sorted reads of HX1? I am not sure why the authors suddenly added a 3rd PUR genome with no context. Was this a mistake? Add results from HX1 to Table 1.

Response: We apologize for any confusion caused. The population data we collected for our study is representative of the Chinese population, and the long-read sorting data mentioned, HX1, is an individual of Chinese. In order to compare the assembly results with the trio binning method, we included the sample HG01109, which is part of a trio dataset consisting of the parental HG01107 and HG01108, along with their offspring HG01109. To clarify this in the manuscript, we have added the following explanation: "We collected a trio dataset consisting of the parental genomes HG01107 and HG01108, as well as the offspring genome HG01109, in order to perform trio binning and compare the resulting assemblies of HG01109." We have now included the results from HX1 in Supplementary Table 6 for a more clear presentation of the comparisons.

8. Please add divider lines in Table 1 between All / Ampliconic / X-degenerate / X-transposed / PAR / Het / Others. It is hard to see which rows belong to which category.
Response: Corrected in Supplementary Table 4-6.

9. The last result section where authors compare results from Verkko, it is unclear how the verkko assembly was run. The authors say "default option", and later "in trio mode" in the methods. Did the authors collect parental reads from HG002 (HG003 and HG004)? How was "trio mode" performed? Did the authors used trio binning to sort the reads, then run Verkko? Or used the homopolymer compressed parental kmers and used that in the Rukki step of Verkko (and this should be benchmarked)? Was the HG002 trio assembly taken from Rautiainen et al. paper? Please clarify and add the missing parts to the main text and methods.
Response: We apologize for the lack of clarity in our manuscript regarding the Verkko assembly. To clarify, we ran the Verkko assembly with the following parameters: "-d Asm --hifi hifi.sorted.fq.gz --nano ul-ont.sorted.fq.gz --threads 128". The HG002 trio assembly used in our study was obtained from the Rautiainen et al. paper. We have updated the methods to include this information.

10. Related to the above section, it is hard to see in Fig. 4a the "two approximately 1 Mb contigs aligning to the same region of the Y chromosome". An enlarged inset of the dotplot may be helpful. Also, add legends and scale to the X and Y axis of the dotplots.
Response: We have updated Figure 4a to include an enlarged inset of the approximately 1 Mb region, and added legends and scale to both X and Y axes of the dotplot. Please see the Fig.4 below:

11. Note there is a mis-assembly reported on T2T-Y palindrome P5 (https://github.com/marbl/CHM13-issues/blob/main/v2.0_issues.bed), which the entire P5 should be inverted. I don't see this in the dotplots of Fig. 4.
Response: Thank you for bringing this to our attention. Upon reviewing the alignment of T2T-Y palindrome P5 (18483410-19235627), we have identified an approximately 3.5kb inversion (18857803-18861259) in the assembly generated by the verkko sry mode. Due to its relatively short alignment length, this inversion may not be clearly visible in the dotplots of Figure 4. Additionally, we have performed assembly using hifiasm in the P5 region and did not find any inversions. This indicates that the current approach (SRY) of read sorting may not effectively resolve the situation with P5. We have made a note of this in Figure 4 to provide clarification.

12. In the discussion, the authors are mentioning results from the 10 trios that have been removed from the previous results. Please add the 10 trio results to the main text if it was a mistake, or remove the irrelevant results from the Discussions and Supp. Tables.
Response: We apologize for any confusion caused. In our study, we collected data from 10 individuals, out of which only HG01109 had trio data. The previous removal referred to the pan-genome analysis of these 10 individuals. The assembly results of the 10 individuals based on SRY sorting were mainly used for comparison with the flow sorting method (Supplementary Table 4 and 6). We have deleted the sentence “we limited our dataset collection to Nanopore or PacBio CLR data for the 10 individuals” in the Discussion section.

13. The authors discuss the suboptimal performance of SRY in the PAR is contributed by the restricted data types. I thought it was contributed by the lower density of the markers? The PAR parental marker density was very similar to that of autosomes, with stretches of runs of homozygosity, presumably to maintain

enough homology for recombination. What was the marker density in the PAR? Was it below their 7 kmer / 1kb?
Response: Thank you for your question. You are correct that the suboptimal performance of SRY in the PAR is due to the lower density of markers. We apologize for any confusion caused by our statement. We have re-examined the data and found that there are no MSK in the PAR region. Considering the coherence of the context, we have removed the sentence in question.

14. The authors mentioned there are no ZW genomes available to test SRY. There is a Zebra finch trio (ZW, female, bTaeGut2) and a male sample (ZZ, male, bTaeGut1) available with HiFi of the child (bTaeGut2) and Illumina of all the genomes from the Vertebrate Genomes Project (Rhie et al., Nature, 2021). Perhaps the authors could apply SRY on this individual, and compare the W chromosome results to what has been released on https://www.genomeark.org/vgp-all/Taeniopygia_guttata.html.
Response: Thank you for providing the information about the availability of ZW genomes for testing SRY. We firstly performed sex determination on seven individuals using the chrW sequence from the reference genome. Our analysis revealed that four individuals were female while three were male. Utilizing SRY, we obtained 19,128,829 specific kmers and sorted approximately 275.8 Mb of HiFi sequences. We assembled the sorted HiFi sequences using hifiasm and verkko softwares. Comparing the two assemblers, we found that hifiasm showed slightly better results (hifiasm total length: 19.7 Mb, N50: 516.3 Kb; verkko total length: 18.1 Mb, N50: 478.3 Kb). The assembly size from hifiasm closely approximated the size of the reference genome (~20.0 Mb when exclude N) and shows good collinearity (figure below). However, our assembly results did not cover the PAR region, which is a limitation of our method.
