# Supplementary material for: An effective strategy for assembling the sex-limited chromosome
Source: Gigascience. 2024 Apr 16;13:giae015. doi: 10.1093/gigascience/giae015 (PMC11020242; doi:10.1093/gigascience/giae015)
Supplement: giae015_Response_to_Reviewer_Comments_Revision_1 [file giae015_response_to_reviewer_comments_revision_1.pdf]

Author's Response To Reviewer Comments

Reviewer #1: After reading the revised article, the questions I had previously posed were answered. I am very interested in this SRY method and believe it is also an important part of sex chromosome research. From my personal point of view, it is not easy to collect Trio data for most species except a few, but it is relatively easy to collect HIC data. It would be helpful if the authors could also compare the results of SRY HIFI with those of Hifiasm (HIC phased) to help people choose the right tool for sex chromosome assembly. However, this is not necessary, because SRY has achieved a very good result in humans. Overall, the data and results are convincing.
Response: Thank you for your thoughtful feedback and interest in our SRY method for sex chromosome research. We will consider incorporating Hi-C data in future versions of SRY and compare with those of Hifiasm or Verkko (HiC phased).

Reviewer #3: The authors have addressed most of my concerns. The revised manuscript reads much better than before.

Regarding my last comment and response from the authors about the W chromosome, I was hoping to see comparable coverage of the W chromosome to the reference, as a proof of principle that SRY could be applied to non-human, highly diverged genomes. The assembly looks very fragmented though. Was it only the similarity to the Z chromosome that caused the fragmentation? Are there no other factors contributing to the discontinuity of the W chromosome?
Response: In response to your question about the fragmentation of the W chromosome in our study, we would like to provide some clarifications. We sorted the W chromosome at a coverage of 14.0X, which is slightly lower than the reference genome's coverage of 18.3X. In highly diverged genomes, the similarity between the W and Z chromosomes primarily affects the assembly of the pseudoautosomal region (PAR). One contributing factor to the fragmentation of the W chromosome assembly is the non-uniform distribution of specific markers. HiFi sequencing data that is predominantly located in regions with lower marker density is difficult to separate, leading to some degree of data loss.
The PacBio CLR data from the 2021 Nature article was at 47.5X for the W chromosome (assembling contig size: 20.0Mb, contig N50: 1.5Mb), while the HiFi sequencing data was less than half of that (assembling contig size: 19.7Mb, contig N50: 0.5Mb). The difference in sequencing data quantity may also have contributed to the fragmented assembly observed.

A few minor comments below to the revised version:
1. Please indicate which genome was compared in the legend of Supp. Table 5.
Response: The genome compared in the legend of Supplementary Table 5 is HG01109. We have made the modification to indicate this.

2. When using `et al` notations, please use the last name. Mari et al should be Serra Mari et al., Mikko et al should be Rautiainen et al. Also, Serra Mari et al is now published in Genome Biology: https://doi.org/10.1186/s13059-023-03160-z. Please update the reference.
Response: Thank you for the clarification. We have now made the adjustments to use the last names when using et al notations. Additionally, we have updated the reference for Serra Mari et al.

3. There are a few grammar corrections to make.
Response: We have made the grammar corrections in the text and highlighted them in blue.
