# Supplementary material for: An effective strategy for assembling the sex-limited chromosome
Source: Gigascience. 2024 Apr 16;13:giae015. doi: 10.1093/gigascience/giae015 (PMC11020242; doi:10.1093/gigascience/giae015)
Supplement: giae015_Reviewer_1_Report_Original_Submission — Zuyao Liu, Ph.D -- 9/1/2023 Reviewed [file giae015_reviewer_1_report_original_submission.pdf]

Reviewer Report

Title: An effective strategy for assembling the sex-limited chromosome

Version: Original Submission **Date:** 9/1/2023

Reviewer name: Zuyao Liu, Ph.D

Reviewer Comments to Author:

The authors have introduced a novel bioinformatic approach for sex chromosome assembly, addressing a persistently challenging problem in genomics. This method harnesses the full potential of whole-genome resequencing data without necessitating supplementary experimental procedures, rendering it applicable to a wide array of non-model species. Notably, the method exhibits robustness when applied to human data, surpassing established techniques such as flow-sorting and trio-binning. While the manuscript exhibits promise, several key aspects warrant refinement and elucidation to bolster its consideration for publication in GigaScience.1. Language Polishing: A degree of language refinement is advisable to enhance the overall clarity and professionalism of the manuscript.2. Y Chromosome Assembly Discrepancy: The authors should acknowledge and provide an explanation for the substantial difference between the length of the latest Y chromosome assembly from T2T (~62Mb) and the assembly from SRY with Verkko (~23Mb), as detailed in Table 1.3. Y Chromosome Completeness: In cases where the Y chromosome assembly is incomplete, the inclusion of a figure or table delineating the proportion that SRY can recover in distinct regions of the Y chromosome would be beneficial. This could facilitate a comparative analysis of the method's efficacy across different regions.4. Figure 4 Clarity: It is imperative to label the coordinates on both the X and Y axes in Figure 4 to enhance clarity. While Figure 4 suggests that the assembly from SRY is complete compared to T2T-CHM13, the total length of the SRY assembly (approximately 23Mb) should be clearly reconciled with this observation.5. Table 1 Organization: The organization of Table 1 should be improved to enhance readability and comprehensibility.6. MSK-Based Read Filtering: Authors should explicitly address the potential exclusion of reads from Y regions with lower than average MSK, especially in species with both young and old parts on Y chromosomes. If possible, provide recommendations or strategies for rescuing such reads.7. Simulation for species with young sex chromosomes: It is essential to conduct additional simulations for testing the efficiency of isolating Y reads for species with young sex chromosomes. This analysis should consider the variation between X and Y chromosomes, aiding researchers in evaluating the method's suitability for their specific study organisms.Addressing these points will further strengthen the manuscript's scientific rigor and its suitability for publication in GigaScience.

Level of Interest

Please indicate how interesting you found the manuscript: Choose an item.

Quality of Written English

Please indicate the quality of language in the manuscript: Choose an item.

Declaration of Competing Interests

Please complete a declaration of competing interests, considering the following questions:

- Have you in the past five years received reimbursements, fees, funding, or salary from an organisation that may in any way gain or lose financially from the publication of this manuscript, either now or in the future?
- Do you hold any stocks or shares in an organisation that may in any way gain or lose financially from the publication of this manuscript, either now or in the future?
- Do you hold or are you currently applying for any patents relating to the content of the manuscript?
- Have you received reimbursements, fees, funding, or salary from an organization that holds or has applied for patents relating to the content of the manuscript?
- Do you have any other financial competing interests?
- Do you have any non-financial competing interests in relation to this paper?

If you can answer no to all of the above, write 'I declare that I have no competing interests' below. If your reply is yes to any, please give details below.

I declare that I have no competing interests' below.

I agree to the open peer review policy of the journal. I understand that my name will be included on my report to the authors and, if the manuscript is accepted for publication, my named report including any attachments I upload will be posted on the website along with the authors' responses. I agree for my report to be made available under an Open Access Creative Commons CC-BY license (http://creativecommons.org/licenses/by/4.0/). I understand that any comments which I do not wish to be included in my named report can be included as confidential comments to the editors, which will not be published.

Choose an item.

To further support our reviewers, we have joined with Publons, where you can gain additional credit to further highlight your hard work (see: https://publons.com/journal/530/gigascience). On publication of this paper, your review will be automatically added to Publons, you can then choose whether or not to claim your Publons credit. I understand this statement.

Yes Choose an item.
