# Supplementary material for: An effective strategy for assembling the sex-limited chromosome
Source: Gigascience. 2024 Apr 16;13:giae015. doi: 10.1093/gigascience/giae015 (PMC11020242; doi:10.1093/gigascience/giae015)
Supplement: giae015_Reviewer_2_Report_Revision_1 — Shilpa Garg -- 2/21/2024 Reviewed [file giae015_reviewer_2_report_revision_1.pdf]

Reviewer Report

Title: An effective strategy for assembling the sex-limited chromosome

Version: Revision 1 **Date:** 2/21/2024

Reviewer name: Arang Rhie

Reviewer Comments to Author:

The authors have addressed most of my concerns. The revised manuscript reads much better than before.Regarding my last comment and response from the authors about the W chromosome, I was hoping to see comparable coverage of the W chromosome to the reference, as a proof of principle that SRY could be applied to non-human, highly diverged genomes. The assembly looks very fragmented though. Was it only the similarity to the Z chromosome that caused the fragmentation? Are there no other factors contributing to the discontinuity of the W chromosome?A few minor comments below to the revised version:1. Please indicate which genome was compared in the legend of Supp. Table 5.2. When using `et al` notations, please use the last name. Mari et al should be Serra Mari et al., Mikko et al should be Rautiainen et al. Also, Serra Mari et al is now published in Genome Biology: https://doi.org/10.1186/s13059-023-03160-z. Please update the reference.3. There are a few grammar corrections to make.

Level of Interest

Please indicate how interesting you found the manuscript: Choose an item.

Quality of Written English

Please indicate the quality of language in the manuscript: Choose an item.

Declaration of Competing Interests

Please complete a declaration of competing interests, considering the following questions:

- Have you in the past five years received reimbursements, fees, funding, or salary from an organisation that may in any way gain or lose financially from the publication of this manuscript, either now or in the future?
- Do you hold any stocks or shares in an organisation that may in any way gain or lose financially from the publication of this manuscript, either now or in the future?
- Do you hold or are you currently applying for any patents relating to the content of the manuscript?
- Have you received reimbursements, fees, funding, or salary from an organization that holds or has applied for patents relating to the content of the manuscript?
- Do you have any other financial competing interests?

- Do you have any non-financial competing interests in relation to this paper?

If you can answer no to all of the above, write 'I declare that I have no competing interests' below. If your reply is yes to any, please give details below.

I declare that I have no competing interests

I agree to the open peer review policy of the journal. I understand that my name will be included on my report to the authors and, if the manuscript is accepted for publication, my named report including any attachments I upload will be posted on the website along with the authors' responses. I agree for my report to be made available under an Open Access Creative Commons CC-BY license (http://creativecommons.org/licenses/by/4.0/). I understand that any comments which I do not wish to be included in my named report can be included as confidential comments to the editors, which will not be published.

Choose an item.

To further support our reviewers, we have joined with Publons, where you can gain additional credit to further highlight your hard work (see: https://publons.com/journal/530/gigascience). On publication of this paper, your review will be automatically added to Publons, you can then choose whether or not to claim your Publons credit. I understand this statement.

Yes Choose an item.
