# Supplementary material for: An effective strategy for assembling the sex-limited chromosome
Source: Gigascience. 2024 Apr 16;13:giae015. doi: 10.1093/gigascience/giae015 (PMC11020242; doi:10.1093/gigascience/giae015)
Supplement: giae015_Reviewer_3_Report_Original_Submission — Arang Rhie -- 10/22/2023 Reviewed [file giae015_reviewer_3_report_original_submission.pdf]

Reviewer Report

Title: An effective strategy for assembling the sex-limited chromosome

Version: Original Submission **Date:** 10/22/2023

Reviewer name: Arang Rhie

Reviewer Comments to Author:

1. In the introduction, add recent marker based graph phasing algorithms in long-reads, such as hifiasm trio and verkko trio mode after the T2T-Y. They are different from trio-binning, which tries to phase the reads upfront. Graph based phasing is using markers to determine haplotype specific paths to traverse.a. T2T-Y chromosome should be referencing Rhie et al., Nature 2023. Verkko is a successor of the manual efforts taken in T2T-Y, which should be also noted in the introduction.b. Reference for sexPhase program is still missing. Also, some rephrasing of the sentence is needed, as the way it is currently written is easily misleading to be understood as sexPhase was part of the methods used in the assembly of the T2T-Y.2. There are other approaches for phasing genomes taken in plants, for example the poly ploid potato phasing using many siblings of the child by Mari et al. bioRxiv 2022.3. "But only one male and one female could suffer from sampling error" - this part is unclear. Please clarify.4. Reference for the mason_simulator, badread software is missing.5. Provide the accession (HG02982) for the "African human Y" in the main text.6. I appreciate that the authors compared assemblies to T2T-Y as I requested before. However, fundamentally, mapping to T2T-Y and comparing length of each sequence classes is comparing apples to oranges, particularly in the heterochromatic region and ampliconic region of the Y. It is known to have variable copy numbers and size differences between two individuals. Frequent inversions have been reported in the ampliconic regions across different Y haplogroup. The number, size, and distribution of the repeat arrays composing the heterochromatic region has been shown to vary among different Y haplogroups in Hallast et al., Nature 2023. This can be also seen in Fig. 3c; the overall depth of the flow sorting in the heterochromatic region is below 1 - indicating the Yqh is shorter than T2T-Y, as it is in Fig. 3b. To make the benchmark legit, the authors should compare SRY and the flow sorting method using samples from the same individual. HG02982 and HX1 are presumably having very different sequence compositions given the diverged population history (African vs. Asian). Comparing total length of the assembled region against a 3rd different Y haplogroup (HG002Y) makes things more complicated, especially on regions that are known to vary a lot. If the authors think flow sorting based method needs to be compared, it should be benchmarked on the same individual to make an apple-to-apple comparison. I do agree results from read sorting (i.e. portion of reads sequenced from non-Y chromosomes in SRY vs. flow-sorting) is an important finding. However, I'd still argue comparing assemblies from the two different Y haplogroups is a stretch. The authors could have performed the same assembly length comparison on the T2T-Y using results from their SRY sorted reads with Verkko of HG002 vs. Verkko assembly using trio-binned markers.7. In the section where assemblies are compared, the authors point to Table 1, which contains results from HG01109. HG01109 has never been mentioned before. I thought the authors were comparing assemblies from SRY sorted reads of HX1? I am not sure why the authors suddenly added a 3rd PUR genome with no context. Was this a mistake? Add results

from HX1 to Table 1.8. Please add divider lines in Table 1 between All / Ampliconic / X-degenerate / X-transposed / PAR / Het / Others. It is hard to see which rows belong to which category.9. The last result section where authors compare results from Verkko, it is unclear how the verkko assembly was run. The authors say "default option", and later "in trio mode" in the methods. Did the authors collect parental reads from HG002 (HG003 and HG004)? How was "trio mode" performed? Did the authors used trio binning to sort the reads, then run Verkko? Or used the homopolymer compressed parental kmers and used that in the Rukki step of Verkko (and this should be benchmarked)? Was the HG002 trio assembly taken from Rautiainen et al. paper? Please clarify and add the missing parts to the main text and methods.10. Related to the above section, it is hard to see in Fig. 4a the "two approximately 1 Mb contigs aligning to the same region of the Y chromosome". An enlarged inset of the dotplot may be helpful. Also, add legends and scale to the X and Y axis of the dotplots.11. Note there is a mis-assembly reported on T2T-Y palindrome P5 (https://github.com/marbl/CHM13-issues/blob/main/v2.0_issues.bed), which the entire P5 should be inverted. I don't see this in the dotplots of Fig. 4.12. In the discussion, the authors are mentioning results from the 10 trios that have been removed from the previous results. Please add the 10 trio results to the main text if it was a mistake, or remove the irrelevant results from the Discussions and Supp. Tables.13. The authors discuss the suboptimal performance of SRY in the PAR is contributed by the restricted data types. I thought it was contributed by the lower density of the markers? The PAR parental marker density was very similar to that of autosomes, with stretches of runs of homozygosity, presumably to maintain enough homology for recombination. What was the marker density in the PAR? Was it below their 7 kmer / 1kb?14. The authors mentioned there are no ZW genomes available to test SRY. There is a Zebra finch trio (ZW, female, bTaeGut2) and a male sample (ZZ, male, bTaeGut1) available with HiFi of the child (bTaeGut2) and Illumina of all the genomes from the Vertebrate Genomes Project (Rhie et al., Nature, 2021). Perhaps the authors could apply SRY on this individual, and compare the W chromosome results to what has been released on https://www.genomeark.org/vgp-all/Taeniopygia_guttata.html.

Level of Interest

Please indicate how interesting you found the manuscript: Choose an item.

Quality of Written English

Please indicate the quality of language in the manuscript: Choose an item.

Declaration of Competing Interests

Please complete a declaration of competing interests, considering the following questions:

- Have you in the past five years received reimbursements, fees, funding, or salary from an organisation that may in any way gain or lose financially from the publication of this manuscript, either now or in the future?

- Do you hold any stocks or shares in an organisation that may in any way gain or lose financially from the publication of this manuscript, either now or in the future?
- Do you hold or are you currently applying for any patents relating to the content of the manuscript?
- Have you received reimbursements, fees, funding, or salary from an organization that holds or has applied for patents relating to the content of the manuscript?
- Do you have any other financial competing interests?
- Do you have any non-financial competing interests in relation to this paper?

If you can answer no to all of the above, write 'I declare that I have no competing interests' below. If your reply is yes to any, please give details below.

I declare that I have no competing interests.

I agree to the open peer review policy of the journal. I understand that my name will be included on my report to the authors and, if the manuscript is accepted for publication, my named report including any attachments I upload will be posted on the website along with the authors' responses. I agree for my report to be made available under an Open Access Creative Commons CC-BY license (http://creativecommons.org/licenses/by/4.0/). I understand that any comments which I do not wish to be included in my named report can be included as confidential comments to the editors, which will not be published.

Choose an item.

To further support our reviewers, we have joined with Publons, where you can gain additional credit to further highlight your hard work (see: https://publons.com/journal/530/gigascience). On publication of this paper, your review will be automatically added to Publons, you can then choose whether or not to claim your Publons credit. I understand this statement.

Yes Choose an item.
